# Self-aligned and self-limiting van der Waals epitaxy of monolayer MoS_2_ for scalable 2D electronics

**DOI:** 10.1038/s41467-026-68320-8

**Published:** 2026-01-21

**Authors:** Yoshiki Sakuma, Keisuke Atsumi, Takanobu Hiroto, Jun Nara, Akihiro Ohtake, Yuki Ono, Takashi Matsumoto, Yukihiro Muta, Kai Takeda, Emi Kano, Toshiki Yasuno, Xu Yang, Nobuyuki Ikarashi, Asato Suzuki, Michio Ikezawa, Shuhong Li, Tomonori Nishimura, Kaito Kanahashi, Kosuke Nagashio

**Affiliations:** 1 Research Center for Electronic and Optical Materials, National Institute for Materials Science, Tsukuba, Japan; 2 Department of Materials Engineering, The University of Tokyo, Tokyo, Japan; 3 Research Network and Facility Services Division, National Institute for Materials Science, Tsukuba, Japan; 4 Research Center for Materials Nanoarchitectonics, National Institute for Materials Science, Tsukuba, Japan; 5 Tokyo Electron Technology Solutions Limited, Hosaka-cho, Nirasaki, Japan; 6 Institute of Materials and Systems for Sustainability, Nagoya University, Furo-cho, Chikusa-ku, Nagoya Japan; 7 Department of Physics, University of Tsukuba, Tsukuba, Japan

**Keywords:** Two-dimensional materials, Electrical and electronic engineering

## Abstract

Unidirectional nucleation followed by seamless stitching has emerged as a promising strategy for the scalable epitaxial growth of single-crystalline monolayer transition metal dichalcogenides on sapphire substrates, which holds potential for post-silicon electronics. In contrast, here we present a different growth mechanism for single-crystalline MoS_2_ on c-plane sapphire via metal-organic chemical vapor deposition (MOCVD). We show that the initial nucleation generates not only 0° and antiparallel 60° domains but also low-angle twisted domains, consistent with the coincidence site lattice framework. However, these rotationally misoriented domains are observed to deterministically self-align and merge into energetically preferred 0° domain during coalescence, yielding a continuous, unidirectional single-crystal. Additionally, by employing MoO_2_Cl_2_ as a molybdenum precursor, we demonstrate that the growth of MoS_2_ occurs in a self-limiting manner. This epitaxial strategy is substantiated by a carrier mobility of 66 cm^2^/Vs at room temperature and 749 cm^2^/Vs at low temperatures. Our approach offers a practical and reproducible scheme for MOCVD-based van der Waals epitaxy for 2D electronics.

## Abstract (web summary)

Previous studies have shown the importance of unidirectionally aligned domain nucleation for the growth of 2D semiconductor single crystals. Here, the authors report the observation of a self-alignment process of misoriented domains during the metal-organic chemical vapour deposition growth of 2D MoS_2_ on sapphire, leading to single-crystalline films with improved carrier mobility.

## Introduction

To fully exploit two-dimensional (2D) transition metal dichalcogenides (TMDCs) for future logic semiconductors at sub-1 nm nodes^1^, owing to their inherent immunity against short-channel effects, scalable epitaxial growth techniques for single-crystalline monolayer TMDC films are in critical demand^2,3^. Scientifically, a thorough and in-depth understanding of pure van der Waals (vdW) epitaxy between 2D materials is indispensable to explore new frontiers in the 2D research field. From an industrial perspective, however, realizing 2D materials on a wafer scale requires prioritized development of quasi-vdW epitaxy on large 3D crystalline substrates such as sapphire (α-Al_2_O_3_), which exhibits superior crystalline quality, exceptional chemical, thermal stability, as well as proven scalability to 300 mm in diameter^4^.

From a purely symmetry-based perspective, heteroepitaxy of a threefold-symmetric monolayer MoS_2_ (D_3h_) on a threefold-symmetric c-plane sapphire (C_3v_) should, in principle, yield a single crystal under equilibrium conditions^5^. In practice, however, the growth occurs under nonequilibrium conditions, and early studies reported the formation of antiparallel domains^6^. Consequently, the control of antiparallel domains has become a critical technical challenge to be addressed in realizing single crystallinity. To overcome these challenges, two major strategies known as step-edge-guided and symmetry-guided epitaxy have been proposed. Specifically, in the step-edge-guided mode^7^, the step edges help to lower the surface symmetry to C_1_ and break the degeneracy of nucleation energy between twined islands, guiding their in-plane orientation along a single preferred direction. In contrast, the crucial role of atomic symmetry at the terrace surface is highlighted in the symmetry-guided epitaxy. A single-type O-Al atomic slab structure with even number of surface steps on c-plane sapphire, which was exposed by deliberately engineered miscut angles, strongly facilitates unidirectional growth of MoS_2_^8^. Consequently, some studies have successfully demonstrated the unidirectionally aligned TMDC films on sapphire substrates by employing techniques such as powder-source chemical vapor deposition (CVD)^7–13^, metal-organic CVD (MOCVD)^14,15^ and other techniques^16^.

However, although both mechanisms are conceptually plausible, their underlying atomistic processes remain elusive. This lack of clarity arises from unresolved questions regarding the surface reconstructions^14,17^ and chemical adsorbates on sapphire in growth environment, the atomic configurations of sapphire step edges^7,8^, and the edge terminations of TMDC grains^18,19^. Moreover, it has been reported that the sulfur to metal precursor supply ratio^13,20–22^, along with the introduction of H_2_^23^, influences the in-plane alignment of TMDC nuclei on sapphire. In fact, these growth conditions can be utilized to underscore and control the favored growth mode separately. Conversely, this implies that both mechanisms inherently coexist in the growth on the same vicinal sapphire surface.

**Fig. 1:**
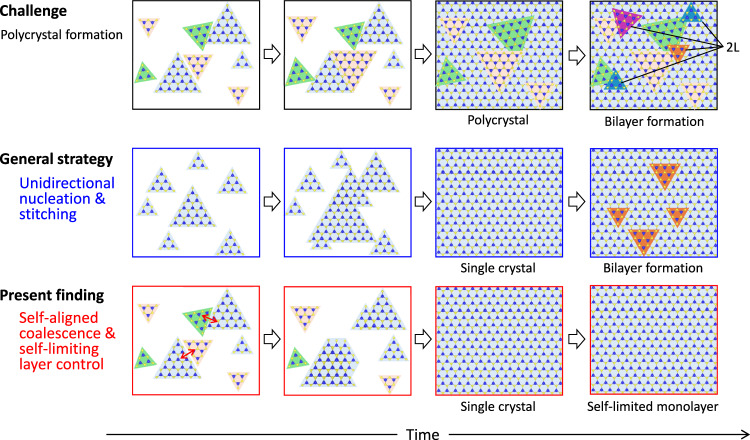
Strategies for wafer-scale epitaxial growth of transition metal dichalcogenides (TMDCs) on sapphire substrates. Schematic illustration showing the challenge for single-crystallization of TMDCs and two kinds of growth strategies for single crystal formation.

Moreover, the formation of various interfacial layers on sapphire surfaces^15,24,25^ has also been identified as playing a critical role in determining the in-plane crystallographic orientations of MoS_2_. Notably, the underlying mechanism responsible for the emergence of MoS_2_ with two distinct orthogonal configurations on c-plane sapphire (0°/60° and 30°/90°) under different experimental conditions remains to be fully elucidated. These complexities impede a comprehensive and unified understanding of quasi-vdW growth mechanism at nucleation stage, particularly pronounced in MOCVD-based research due to limited investigations. Nonetheless, it should be emphasized that all the above-mentioned studies are based on a common approach, in which aligning all the nuclei in the same in-plane orientation and subsequent seamless stitching are regarded as a guiding principle^26^, as depicted in Fig. 1.

In this study, unlike the conventional strategy, we report a fundamentally distinct self-aligned coalescence mechanism for wafer-scale single-crystalline MoS_2_ films in an MOCVD-type reactor using MoO_2_Cl_2_ and H_2_S as precursors, as shown in Fig. 1. Besides antiparallel nuclei, isolated MoS_2_ domains initially nucleated with well-defined in-plane twisted angles elucidated by coincidence site lattice (or supercell) model between MoS_2_ and sapphire. Subsequently, upon domain impingement, both the low-angle twisted and antiparallel 60° domains are self-aligned and merged into the most energetically favorable 0° domain during coalescence process. Consequently, only the 0° domain survives, culminating in a deterministic formation of continuous, unidirectionally aligned single-crystalline MoS_2_ film. Another noticeable feature of MoS_2_ growth using MoO_2_Cl_2_ precursor is a self-limiting manner over a wide range of growth conditions. As a proof of high-quality MoS_2_ films with negligible grain boundaries, carrier transport exhibited typical power law behavior, yielding high electron mobilities of 66 cm^2^/Vs at room temperature and approximately 749 cm^2^/Vs at low temperatures.

## Results

### Epitaxial registry and self-aligned coalescence

**Fig. 2:**
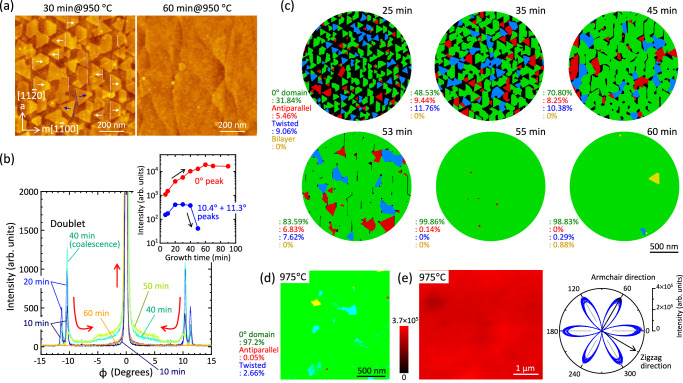
Annihilation of antiparallel & low-angle twisted domains during coalescence of MoS_2_ domains on sapphire substrates. **a** Atomic force microscope (AFM) images of MoS_2_ grown on the sapphire substrate at 30 and 60 min at 950 °C. **b** in-plane X-ray diffraction (XRD) intensity around the 0° peak for the ϕ scan of MoS_2_ acquired at different growth durations at 975 °C. The inset shows the intensities of XRD peaks at 0° and ~ 11° as a function of growth durations. The sum of the intensity of doublet subpeaks at 10.42° and 11.32° was plotted as ~11°. **c** False-color maps obtained by time-resolved differential dark-field transmission electron microscopy (DF-TEM) analysis of MoS_2_ grown at 975 °C. The area ratio of each domain was calculated based on the absolute coverage. **d** four-dimensional-scanning transmission electron microscopy (4D-STEM) data of MoS_2_ grown at 975 °C. **e** Second harmonic generation (SHG) intensity map and polarization dependence of SHG at different 25 locations (see Supplementary Fig. 13) for MoS_2_ grown at 975 °C.

An industrially compatible c-plane sapphire substrate with an orientation flat plane of ($\bar{1}\bar{1}20$) and a miscut angle of 0.15°–0.2° toward m-axis of [$1\bar{1}00$] direction (notated as c/m: −0.15° or −0.2°), manufactured by Orbray Co. Ltd., was utilized as a standard substrate in this study. Prior to growth, the substrate was annealed in air for 1 hour at 1150 °C to form parallel monolayer atomic steps along a-axis of [$11\bar{2}0$] with a terrace width of approximately 60–80 nm^27^. Monolayer MoS_2_ films were grown via MOCVD reactor, utilizing MoO_2_Cl_2_ and H_2_S as precursors in N_2_ carrier gas. Growth temperatures ranged from 800 °C to 1050 °C, with a maximum growth duration of 2 hours (see Methods). The detailed description of the MOCVD process steps can be seen in Supplementary Fig. 1. Notably, MoO_2_Cl_2_ precursor not only exhibits adequate volatility comparable to that of conventional Mo(CO)_6_ around room temperature, but also the advantage of eliminating carbon contamination^28^. At a growth temperature of 950 °C and a duration of 30 min, isolated MoS_2_ domains were clearly observed, as shown in Fig. 2a. These domains comprised antiparallel triangular domains of 0° and 60°, approximately 150 nm in size, oriented along [$1\bar{1}00$] and [$\bar{1}100$], respectively. In terms of the quantity ratio of their domains, approximately 55% exhibited 0°, with the remainder at 60°. Upon closer inspection, however, ~11° and ~49° domains were also discerned, as shown by blue dotted lines. Extending the growth duration to 60 min resulted in a fully continuous monolayer MoS_2_ film. It is noteworthy that the terrace width of the sapphire surface increased after the growth significantly^14^ probably due to the highly reactive nature of H_2_S gas at elevated temperatures exceeding 950 °C (Supplementary Fig. 2). For the fully-covered sample, the epitaxial relationship of [$11\bar{2}0$]MoS_2_//[$11\bar{2}0$]α-Al_2_O_3_ was confirmed through in-plane X-ray diffraction (XRD) ϕ scans (Supplementary Fig. 3), which is consistent with the previous literature for MoS_2_ growth on sapphire substrates by MOCVD^17,29^ and powder-source CVD^6,21,30^.

We performed a detailed analysis of the orientational relationships between the MoS_2_ and sapphire substrate for isolated domains grown at 975 °C for 20 min. As depicted in Supplementary Fig. 4, high-resolution in-plane XRD measurement revealed the presence of additional subpeaks at azimuthal angles of approximately 10.87° (average of doublet), 19°, 30°, 41°, and 49.13° between the principal peaks at 0° and 60°, which are equivalent to 0° ± 10.87°, 30°±10.87°. Although some of these subpeaks were also reported previously^14,31,32^, earlier works do not have quantitatively captured either the presence or the true areal fraction of twisted domains due to the limited resolution and scan rates, compared with the present precise measurement at step of 0.02°, scan speed of 0.3°/min (total time for 180° scan is 10 hours). Therefore, their origin and implications have yet to be apparently elucidated. The observed azimuthal angles exhibit excellent agreement with those derived from supercells between the commensurate MoS_2_ and c-plane sapphire substrate, as detailed in the table of Supplementary Fig. 4^6^. Moreover, the subpeak with 10.87° twisted angle was resolved into a doublet at 10.42° and 11.32° (average: 10.87°), although the underlying mechanism remains unclear. Notably, the prominent peaks at 0° and 10.87° with large XRD intensities correspond to the two smallest supercell sizes in the table, suggesting that the epitaxial registry of tiny nuclei at the early stage of MoS_2_ growth is likely governed by coincidence site lattice model. The presence of isolated twisted domains expected from this in-plane XRD appears to be in good agreement with the AFM images of the early-stage sample after 4 min of growth, as shown in Supplementary Fig. 5.

The time evolution of MoS₂ growth at 975 °C was systematically examined to elucidate the pathway to full surface coverage, as depicted in Supplementary Fig. 6. Figure 2b overlays the data corresponding to the 0° domain at different growth durations, highlighting the correlation between primary 0° peak and doublet 10.42°/11.32° subpeaks. Intriguingly, the intensity of these doublet peaks initially exhibited a monotonic increase with the growth duration, followed by a rapid decrease around 50 min, ultimately vanishing altogether. Concurrently, a broad tail structure at the foot of the 0° peak emerged at approximately 40 min, and disappeared by 60 min. Throughout this process, the area intensity of the primary 0° peak exhibited a continuous increase, as shown in the inset of Fig. 2b. To attain the real-space observation of this coalescence processes, the technique using differential dark-field transmission electron microscopy (DF-TEM) analysis was developed here. While in-plane XRD lacks the sensitivity to unambiguously resolve antiparallel domains of 0° and 60°, electron diffraction techniques are capable of their clear differentiation^33^. Although Friedel’s law describes that the intensities of *hkl* and *-h-k-l* reflections are equivalent even for polar crystals, this equivalence is violated in monolayer TMDCs owing to the broken in-plane inversion symmetry and multiple scattering effects, resulting in anomalous diffraction intensity contrasts^34^. As shown in Supplementary Fig. 7, therefore, by taking the difference between DF-TEM images obtained from a pair of diffraction spots related by 180° inversion, where Friedel’s law is violated, 0° and 60° antiparallel domains are unambiguously distinguished. Moreover, comparison with the corresponding bright-field (BF) TEM image allows for the discrimination of low-angle twisted domains in addition to the 0° and 60° antiparallel domains, thereby enabling the construction of comprehensive false-color maps for monolayer MoS_2_ grown at 975 °C for various growth durations, as shown in Fig. 2c. From 25 to 35 min, nearly all domains retain their triangular morphology and a substantial fraction of both 60° antiparallel and low-angle twisted domains clearly remains. Interestingly, from 45 to 53 min, 60° antiparallel domains as well as low-angle twisted domains, which was observed in in-plane XRD, are significantly reduced due to coalescence and almost annihilated by 55 min, resulting in the fully covered single-crystalline MoS_2_ film. It should be emphasized that such systematic time evolution is uniquely accessible through the highly reproducible nature of MOCVD growth, as demonstrated in Supplementary Fig. 8, whereas acquiring such detailed and consistent data is exceedingly difficult with conventional powder-source CVD.

These observations strongly indicate the following scenario. In the early stage of growth, small nuclei formed independently while maintaining their twisted angles elucidated by supercell model. Upon impingement of the domains in lateral at approximately 40 min, the low-angle twisted and the antiparallel 60° domains started to merge into the energetically favorable 0° domain to form a single crystal via the self-aligned seamless stitching. Finally, complete surface coverage was attained by 60 min. Crucially, differential DF-TEM images obtained at ten different locations across the 3-mmϕ TEM grid, as shown in Supplementary Fig. 9, clearly confirm the single-crystalline nature of the film with only minimal occurrences of antiparallel and twisted orientations. Here, it is important to note that, using the time-resolved in-plane XRD, this time evolution was also observed at a reduced temperature of 950 °C (Supplementary Fig. 10) and even with alternative precursors of Mo(CO)_6_ and H_2_S at 950 °C (Supplementary Fig. 11), suggesting that the formation of single crystals via merging and seamless stitching following the impingement is a generalizable phenomenon, not limited to the MoO_2_Cl_2_ precursor.

To further gain profound insights into the coalescence process of MoS_2_ grains in fully continuous films grown at different temperatures, four-dimensional-scanning transmission electron microscopy (4D-STEM)^29,35^ and polarization-resolved second harmonic generation (SHG)^36^ was utilized to visualize the orientation of grains at the multiple spatial resolutions from nanometer to micrometer, as illustrated in Fig. 2d, e and Supplementary Figs. 13. As the growth temperature increases, the low-angle twisted domains and antiparallel domains, initially incorporated into the dominant 0° domains at 900 °C, were significantly reduced, eventually disappearing almost entirely at 975 °C. These observations strongly suggest that a relatively high temperature of 975 °C is critical for achieving complete single-crystal formation via self-aligned coalescence. Although self-aligned single crystallization at low temperature is theoretically achievable, it has proven experimentally impractical, as shown in Figure 14. Moreover, SHG is highly sensitive to strain levels as low as 0.1%, which remain undetectable by Raman spectroscopy^37^. The polar plot of the parallel SHG component for MoS_2_ grown at 975 °C reveals a uniform six-fold symmetry with maximum intensity along the armchair direction, confirming the absence of substantial residual strain in the film.

### Deterministic epitaxial alignment of MoS_2_/sapphire

**Fig. 3:**
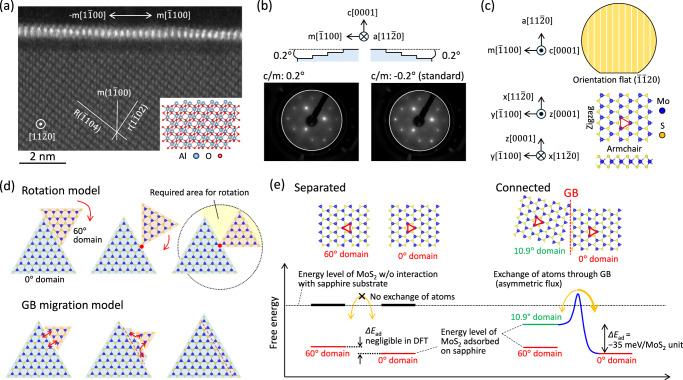
Determination of unique MoS_2_/sapphire orientation & growth mechanism. **a** Cross-sectional high-angle annular dark-field STEM (HAADF-STEM) image of monolayer MoS_2_ grown on the sapphire substrate at 975 °C. **b** Low-energy electron diffraction (LEED) patterns of MoS_2_ grown on sapphire substrates with opposing miscut angles. **c** Definition of orientation for the sapphire substate and monolayer MoS_2_. Note that the orientation of monolayer MoS_2_ is defined by the bottom layer of the unit cell, as specified in the standard crystallographic information (cif) file. **d** Illustration of two distinct growth mechanisms. **e** Schematic representation of the energy relationship between the 0°, 10.9° and 60° domains with adsorption energy differences calculated by density functional theory (DFT). GB: grain boundary, *ΔE*_ab_: adsorption energy difference. Yellow arrow indicates the direction of atomic diffusion.

The unique phenomenon of single-crystal formation via self-aligned coalescence, as observed in Fig. 2c, suggests the existence of a deterministic crystallographic orientation of MoS_2_ on the sapphire substrate. Figure 3a presents a cross-sectional high-angle annular dark field scanning TEM (HAADF-STEM) image of MoS_2_ grown on the sapphire substrate at 975 °C, observed from the [$11\bar{2}0$] direction. The r($1\bar{1}02$), R($\bar{1}104$), and m($1\bar{1}00$) faces of the sapphire substrate were clearly discerned, corroborating the crystallographic asymmetry along +m[$\bar{1}100$] and -m[$1\bar{1}00$], i.e., the threefold symmetry of sapphire, depicted in the inset. Previous studies have discussed the presence of a buffer layer on the sapphire substrate^13,15,22^. In our HAADF-STEM image, this buffer layer exhibited weaker contrast than in previous report, which likely facilitates the formation of an epitaxial relationship between MoS_2_ and sapphire. Although a vdW MoS_2_/sapphire distance was observed as ~0.7 nm for bottom S-top Al distance^7,8,13,25^, the precise in-plane atomic alignment of MoS_2_ on the sapphire substrate remained unclear.

To clarify this, low-energy electron diffraction (LEED) measurements were performed for monolayer MoS_2_ grown on the standard sapphire substrate (c/m = −0.2°) at 975 °C at specific voltage of 190 eV across eight positions, each spaced 1 mm apart. As shown in Fig. 3b and Supplementary Fig. 15, all exhibited a consistent threefold symmetric diffraction pattern of single-crystalline monolayer MoS_2_^7,13^, indicating the uniform alignment of MoS_2_ on a wafer scale. Furthermore, the atomic configuration of monolayer MoS_2_ on the sapphire substrate was uniquely determined by analyzing the LEED intensity-voltage (*I*-*V*) profiles from 10 and 01 LEED spots. It was revealed that the MoS_2_ structural model shown in Fig. 3c can be directly overlaid onto the sapphire substrate schematically drawn. Additionally, when monolayer MoS_2_ was grown on custom-ordered sapphire substrates with miscut angle of 0.2° along +m-direction (c/m = +0.2°) and a-direction (c/a = +0.15°), identical LEED patterns were obtained irrespective of the miscut direction, as shown in Supplementary Fig. 15. The confirmation of miscut angle of sapphire substrates can be found in Supplementary Fig. 16. Given the inherent threefold symmetry of the c-sapphire surface, variations in the miscut direction inevitably lead to differences in the atomic configurations at the step edges. Therefore, it can be concluded that the orientation of MoS_2_ during the MOCVD growth is governed not by the step edges but by its interaction with the surface structure of the sapphire substrate. This interpretation is further supported by the AFM images in Supplementary Fig. 5, which show that the initial growth nuclei on sapphire are not influenced by the surface steps. Furthermore, as shown in Supplementary Fig. 2, the terrace width increases dynamically during MoS_2_ growth. A recent study highlighted the role of step-edge reconstructions on sapphire surfaces in guiding epitaxial alignment during the nucleation stage^12^. In contrast, the present work emphasizes the subsequent coalescence stage, not the nucleation stage, which constitutes a critical distinction between the two studies. Consequently, the growth process reported here does not require fine control of the miscut direction to tailor the atomic configuration of steps and terraces, thereby ensuring both robustness and reproducibility.

Thus, the orientation of MoS_2_ on the sapphire substrate grown at 975 °C is uniquely determined, with the MoS_2_ [$11\bar{2}0$] and [$\bar{1}100$] directions aligning with the sapphire [$11\bar{2}0$] and [$\bar{1}100$] directions, respectively. MoS_2_ with this orientation is indeed the 0° domain depicted in Fig. 3c. It should be noted that this configuration matches that reported in previous growth studies (0°/60°)^6^, while differing from the orientation relationships observed in other reports (30°/90°)^7,8,13,38^, as illustrated in Supplementary Fig. 17. As mentioned in the *introduction*, this may be related to specific interfacial and buffer layers on sapphire surface caused by the precursor supply ratio (S/Mo)^12,21,22^.

Subsequently, we aim to explore the mechanism by which low-angle twisted and antiparallel domains are annihilated. As illustrated in Fig. 3d, two mechanisms can be considered: the rotation model and the grain boundary (GB) migration model. In general, the rotation of antiparallel grains in opposite orientation might be possible in vdW heteroepitaxy due to the weak interaction^39^. However, time-resolved differential DF-TEM images in Fig. 2c clearly indicate that there is inadequate space to accommodate the rotation process when coalescence initiates ~40 min after the commencement of growth. In contrast, GB migration model involves mass transfer between adjacent grains across the boundary via atomic diffusion within the solid, without requiring additional spatial allowance. When low-angle and antiparallel domains are in physical contact with 0° domains, the movement of Mo and S atoms, facilitated by vacancies and interstitials, will drive GB migration. Therefore, a diffusion-coupled GB migration model is the most plausible explanation.

It is also important to discuss the apparent onset time of coalescence, around 40 min, as indicated in Fig. 2c. Notably, residual low-angle and antiparallel domains are consistently accompanied by ungrown peripheral regions (black contrast). This correlation strongly suggests that GB migration is interrupted at ungrown regions. In other words, sustained GB migration toward a unidirectional single crystal proceeds only after individual misoriented domains become fully surrounded by 0° domain, that is, when the GB network becomes continuous and is no longer interrupted by ungrown regions.

This picture implies that GB migration also contains tangential motion along the GB, leading to progressive shrinkage and eventual disappearance of the enclosed misoriented domains. This process starts locally as the coverage of MoS_2_ on the sapphire substrate increases, and then propagates across the wafer. After 60 min, when MoS_2_ fully covers the substrate, the self-aligned coalescence is completed over the entire wafer, resulting in wafer-scale single crystallization. This evolution likely explains why full coverage and wafer-scale single crystallization occur nearly simultaneously at around 60 min. Therefore, under our growth conditions, the local surface coverage required to form fully enclosed regions is first achieved at approximately 40 min. This time point can thus be regarded as the apparent “threshold” for the onset of self-aligned coalescence in our experiments. Additional experiments supporting these growth mechanisms are presented in Supplementary Fig. 18.

The self-aligned growth observed here has not been reported in previous studies. Nevertheless, twisted domains are not unique to our samples; they have also been observed in MOCVD- and MBE-grown films from other groups^14,31,32^. A key distinction is that such films have seldom been examined using sufficiently high-sensitivity characterization. In our in-plane XRD measurements, for instance, we employ the conditions of slow scan speed so that the subpeaks from the rotational domains can be observed with a sufficiently good signal-to-noise ratio. Moreover, many powder-CVD reports primarily present AFM images of comparatively large domains (~10–100 μm), without resolving the early nucleation regime. To date, no study has systematically and quantitatively traced the full evolutionary pathway—from the emergence and coalescence of smaller, rotated early-stage nuclei to the ultimate seamless stitching into large single-crystal domains—through a combined analysis using in-plane XRD, low-magnification DF-TEM, and 4D-STEM. This gap stems in part from the experimental difficulty of conducting time-resolved evolution studies in powder-CVD. Consequently, the existing evidence remains inadequate to support a definitive conclusion.

The orientation-selective growth process is classically recognized as epitaxial grain growth (EGG) in polycrystalline thin films on single crystalline substrates^40^. EGG is driven by the minimization of crystallographically anisotropic free energies, such as surface and interface energies. At the monolayer limit of 2D materials, as discussed in Supplementary Fig. 19, MoS_2_/sapphire interface energy is expected to play a dominant role. Therefore, to elucidate the driving force, density functional theory (DFT) calculations were conducted to compute the adsorption energies between the MoS_2_ and sapphire substrate at various twisted angles, assuming commensurate infinite monolayer MoS_2_ slabs, as shown in Supplementary Fig. 20. Figure 3e summarizes the relative adsorption stabilities of the 0°, antiparallel, and low-angle twisted domains. In isolation from the substrate, all domains are energetically equivalent. However, during coalescence, the merging of 10.9° domains into 0° domains is driven by an adsorption energy difference of *ΔE*_ad_ = −35 meV/MoS_2_ unit via the GB migration. On the other hand, *ΔE*_ad_ between 0 and 60 antiparallel domains should exist but was negligibly small (~−0.1 meV/MoS_2_ unit), indicating that the grain boundary energy must be considered in further studies, as discussed in Supplementary Fig. 20. Nevertheless, it is important to emphasize that even such small energy differences can induce GB migration, as reported for adjacent graphene islands on Ir(111), where the driving force was on the order of 0.1 meV per C atom^39^. The present energy considerations are supported by the differential DF-TEM and 4D-STEM observations (Fig. 2c, d  and Supplementary Fig. 13).

### Wafer scale uniformity via self-limiting growth

**Fig. 4:**
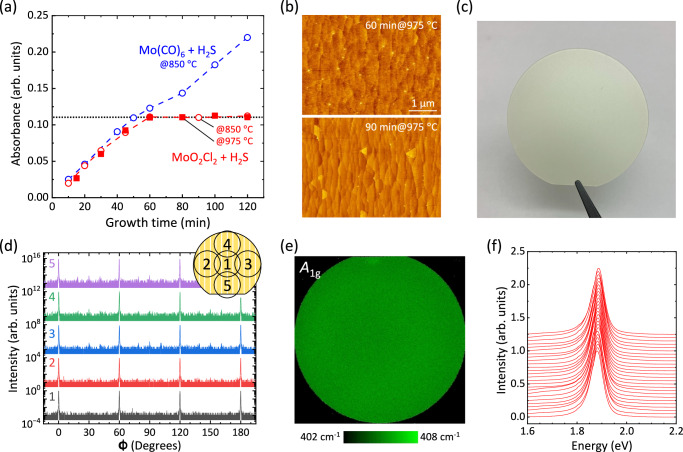
Wafer scale uniformity via self-limiting growth. **a** Absorbance of MoS_2_ grown on sapphire substrates at 850 °C and 975 °C as a function of growth durations using different precursors. In case of Mo(CO)_6_ precursor, only data at 850 °C is shown. The horizontal dashed line indicates the absorbance of fully-covered monolayer MoS_2_. **b** AFM images of MoS_2_ grown on sapphire substrates at 975 °C at various growth durations. **c** Photograph of a 2-inch monolayer MoS_2_/c-sapphire substrate. **d** In-plane XRD ϕ scans taken at five different locations, which is shown in the inset, for monolayer MoS_2_ grown on a 2-inch sapphire substrate at 950 °C. **e** Raman *A*_1g_ intensity map for monolayer MoS_2_ grown on a 2-inch sapphire substrate at 1025 °C. **f** Photoluminescence (PL) line scans along the wafer for monolayer MoS_2_ grown on a 2-inch sapphire substrate at 950 °C.

To guarantee the reliable performance of electronic devices whose properties are highly sensitive to the precise number of 2D material layers, self-limited layer-controlled synthesis provides great technical advantages. While atomic layer deposition (ALD) is among the most promising techniques owing to its surface adsorbed growth mechanism, substantial progress is still required to enhance the crystallinity of MoS_2_ films^41,42^. In the epitaxial growth of TMDC on the sapphire substrate via MOCVD, precise layer control remains a significant challenge, primarily due to the nonuniform gas flow pattern across wafer surface and the consumption of precursors along the gas flow direction in reactor^24,43,44^. Here, although the MoO_2_Cl_2_ and H_2_S precursors were supplied simultaneously, unlike ALD, a self-limited monolayer growth of MoS_2_ was demonstrated as shown in Fig. 4a and Supplementary Fig. 21. The absorbance at C exciton measured for MoS_2_ films, grown on double-side polished sapphire substrates at 850 °C, saturates beyond 60 min, in contrast to the continuous growth tendency observed using Mo(CO)_6_ and H_2_S precursors. Moreover, the self-limiting growth is rather profound at an elevated temperature of 975 °C under optimized growth conditions, as shown in Fig. 4a and Supplementary Fig. 22. This is further supported by AFM images in Fig. 4b and Supplementary Fig. 23, where no voids and very limited second layer growth of MoS_2_ are discernable even at the growth duration longer than 60 min. Moreover, the tail of main in-plane XRD peak at 0° in Fig. 2b and Supplementary Fig. 6 no longer broadens for MoO_2_Cl_2_, while broad tail behavior observed due to the mosaic spread caused by the second epitaxial layer of MoS_2_ for Mo(CO)_6_ in Supplementary Fig. 11. The mechanism of this self-limiting is attributed to site-selective adsorption, wherein the MoO_2_Cl_2_ precursor, unlike Mo(CO)_6_, exhibits a low affinity for adsorption on the MoS_2_ surface. The detailed discussion can be found in Supplementary Fig. 22.

This self-limited layer-controlled synthesis enables wafer-scale uniformity in MoS_2_ growth, as shown by a 2-inch monolayer MoS_2_/c-sapphire substrate in Fig. 4c. The quality and uniformity of the as-grown monolayer MoS_2_ films are illustrated via multi-scale characterizations. High-resolution in-plane XRD ϕ-scans, performed at five different points on the wafer (Fig. 4d), macroscopically demonstrate the formation of a uniform film without low-angle twisted domains throughout the wafer. The variation in Raman *A*_1g_ peaks of the monolayer MoS_2_ is only 2 cm^-1^ throughout the 2-inch wafer, as shown in Fig. 4e and Supplementary Fig. 24. Moreover, Fig. 4f shows photoluminescence (PL) line scans across a 2-inch MoS_2_ wafer with 2 mm step. No obvious variations in peak position and line width were observed. These results collectively demonstrate the excellent uniformity and high reproducibility of MOCVD-grown MoS_2_ films, spanning from the sub-micrometer to centimeter scale.

### Transport properties of monolayer MoS_2_

**Fig. 5:**
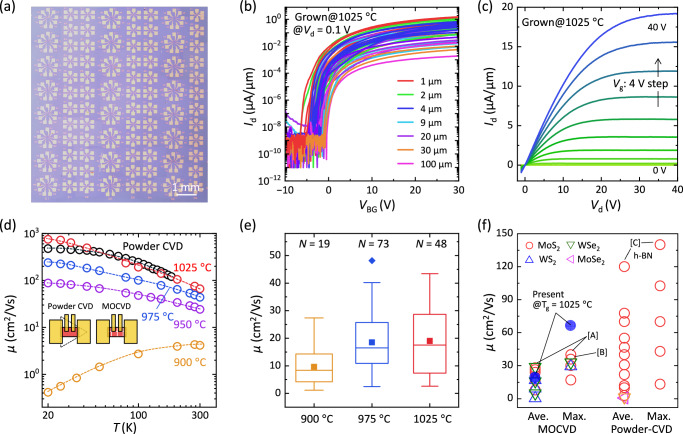
Transport properties of monolayer MoS_2_. **a** Photograph of monolayer MoS_2_ field-effect transistors (FETs) on a SiO_2_/Si substrate. **b** Transfer characteristics of 48 MoS_2_ FETs grown at 1025 °C. *V*_BG_: back-gate voltage, *I*_d_: drain current, *V*_d_: drain voltage. **c** Output characteristics of MoS_2_ FET grown at 1025 °C. **d** Temperature dependence of mobility for four-terminal MoS_2_ FETs grown at various temperatures. Colored dashed curves are eye guides, while black circles represent reference data for powder-source CVD^49^. **e** Statistical analysis of mobility for two-terminal MoS_2_ FETs grown at various temperatures, where the Y-function method^56^ was applied for evaluation. N represents the number of measured devices. The box represents the interquartile range, the line inside the box indicates the median, and the whiskers show the spread of the data. **f** Benchmark of mobility values for TMDC grown by powder-source CVD and MOCVD. Refs. [A], [B], and [C] are adapted from refs. ^44,57^, and ^13^.

To evaluate the electronic performance of monolayer MoS_2_, Hall bar and two-terminal field-effect transistor (FET) configurations were prepared via a standard photolithography process after transferring MoS_2_ to a SiO_2_/Si substrate, as shown in Fig. 5a and Supplementary Fig. 25. Figure 5b exhibit transfer curves with limited variation obtained from MoS_2_ grown at 1025 °C, showing the on/off current ratio exceeding ~10^7^ and clear saturation. Figure 5c shows output characteristics obtained from MoS_2_ grown at 1025 °C, showing ohmic behavior using Ni/Au contacts. Moreover, short-channel devices with channel lengths ranging from 2 μm down to 51 nm were fabricated using electron beam lithography, exhibiting an increase in drain current with decreasing channel length, as shown in Supplementary Fig. 26. This trend highlights their relevance to the practical realization of 2D nanoelectronics.

The mobilities achieved via MOCVD so far are comparatively lower than those obtained using powder-source CVD^2^, rendering small comparisons of *μ* values at room temperature less meaningful. In stead, our primary focus is placed on the temperature dependence of mobility (*μ*)^45,46^, rather than its absolute vale, as this provides clearer insight into the underlying electron transport mechanisms. The reports on the temperature dependence of mobility in MOCVD-grown MoS_2_ films have been considerably limited^47,48^, compared with MoS_2_ synthesized via powder-based CVD methods. Figure 5d and Supplementary Fig. 27 summarize the *μ* values of MoS_2_ channel, obtained by four terminal measurements, as a function of temperature for MoS_2_ grown under different conditions. As the growth temperature increases from 900 °C to 1025 °C, a marked transition from thermally activated behavior to typical power law behavior (*μ* ~ *T*^−γ^) above 100 K. The temperature coefficient (*γ*) in this power law is discussed based on the effect of the substrate, as shown in Supplementary Fig. 28. The temperature-dependent *μ* for MoS_2_ grown at 1025 °C attains a high mobility, approximately 66 cm^2^/Vs at room temperature and 749 cm^2^/Vs at 20 K, for monolayer MoS_2_ synthesized by MOCVD. Furthermore, this temperature dependence approaches values similar to the best data achieved previously with powder-source CVD^49^, where large single-crystal triangles have been measured on the SiO_2_/Si substrate, strongly indicating the single crystal formation by the annihilation of GBs. It is noteworthy that MoS_2_ properties were characterized on 2 × 2 cm^2^ sapphire wafers, while devices were fabricated using 2-inch wafers. The 2-inch wafers require ~50 °C higher substrate temperatures due to different MOCVD susceptors. The *μ* values of two-terminal MoS_2_ FETs in Fig. 5e show that the average room-temperature *μ* increases with increasing growth temperatures. Finally, Fig. 5f benchmarks the maximum and average *μ* values at room temperature, with the current study reporting the highest value to date. The mobilities reported here remain lower than those obtained via powder-source CVD. This discrepancy suggests that a higher defect density may still limit carrier transport. The systematic defect characterization and subsequent reduction will be essential to further enhance mobility, thereby bridging the gap with powder-source CVD.

## Discussion

In this study, we have revealed a self-aligned and self-limiting vdW epitaxy for unidirectional single-crystalline MoS_2_ film on c-plane sapphire, by employing high-precision, time-resolved in-plane XRD, differential DF-TEM and 4D-STEM. The epitaxial relationship between MoS_2_ and sapphire substrate was deterministically elucidated, with the MoS_2_ [$11\bar{2}0$] and [$\bar{1}100$] directions aligning with the sapphire [$11\bar{2}0$] and [$\bar{1}100$] directions, respectively. Our results demonstrate the superiority of MOCVD technology in growing monolayer TMDCs, providing a major impetus for future industrialization.

## Methods

### MOCVD growth

The MOCVD reactor employed in this study is horizontal-flow and cold-wall configuration. The graphite susceptor for single wafer is resistively heated from its backside and the maximum wafer size is 2 inches (Supplementary Fig. 1). Molybdenum oxychloride (MoO_2_Cl_2_) and hydrogen sulfide (H_2_S) were utilized as the molybdenum and sulfur precursors, respectively. The MoO_2_Cl_2_ canister was controlled at atmospheric pressure and bath temperature of 18 °C. The flow rate of N_2_ passing through the canister was varied from 50 to 400 sccm to regulate the supply of MoO_2_Cl_2_. The H_2_S flow rate was changed within the range of 2 to 20 sccm. The total N_2_ flow rate in the reactor was kept at 2500 sccm. MoS_2_ film was grown at substrate temperatures ranging from 800 °C to 1050 °C, under a constant chamber pressure of 50 Torr, with growth durations limited to a maximum of 2 h. All the gas flow rates and pressures were controlled by mass flow controllers and automatic pressure controllers, respectively. Alternatively, precursors of Mo(CO)_6_ and H_2_S were also utilized for MOCVD growth run for comparison. The Mo(CO)_6_ bath temperature is 5 °C and the flow rate N_2_ carrier gas is fixed at 20 sccm. Calculating from the sublimation pressures of MoO_2_Cl_2_ and Mo(CO)_6_ described in Supplementary Fig. 1c, all the MoS_2_ growth was conducted under sulfur-rich conditions. Sapphire substrates cutting into 2 × 2 cm^2^ were usually employed for the growth experiments, while 2-inch diameter wafers were utilized for the MoS_2_ growth used for device fabrication. The 2-inch c-sapphire substrates were procured by Orbray Co. Ltd. Prior to growth, thermal annealing process was performed in a muffle furnace at 1150 °C in air for 1 h to prepare surface steps aligned along the a-axis [$11\bar{2}0$].

### Characterizations

To confirm the crystal quality and thickness of films, Raman spectroscopy, PL spectroscopy and AFM were employed. Raman and PL measurements were conducted with a 532-nm excitation laser with an ~1 μm spot and ~0.5 mW. In-plane XRD measurements were performed using a Rigaku SmartLab system to identify the presence of low-angle domains in monolayer MoS_2_ (Supplementary Fig. 3). Initially, the diffraction peak corresponding to the {$11\bar{2}0$} plane of MoS_2_ was observed to align with the {$11\bar{2}0$} plane of the sapphire substrate, confirmed via a 2θχ/ϕ scan. Subsequently, a ϕ scan of {$11\bar{2}0$} planes of MoS_2_ was conducted with a typical scan rate of 0.3°/min and step width of 0.02°. Differential DF-TEM, 4D-STEM and HAADF-STEM experiments were carried out using a JEOL ARM 200 F TEM with acceleration voltages of 80, 200, and 200 kV, respectively. 4D-STEM orientation maps were generated from electron diffraction data set using a proprietary algorithm based on the NanoMEGAS ASTAR. For HAADF-STEM, the spherical aberration coefficient was less than 1 μm. The convergent angle was 17 mrad and the inner and outer angles of the ADF detector were 50 and 150 mrad, respectively. The SHG measurements were conducted using a mode-locked Ti:sapphire laser (wavelength: 810 nm, pulse width: ~100 fs, and repetition rate: 82 MHz) in a home-built optical microscope configured for backscattering. The LEED patterns at room temperature were acquired using a conventional LEED system (OCI, BDL600IR) under ultrahigh vacuum (UHV) conditions at 10^−^^8 ^Pa, with a 1 eV step in the energy range from 30 to 380 eV. The LEED *I-V* curves for five non-equivalent beams were extracted from LEED patterns with the background subtracted. The total cumulative energy range covered was approximately 1455 eV.

Theoretical calculations were performed by using the PHASE/0 code^50^, which is based on DFT^51^ and pseudo-potential schemes^52,53^ with plane-wave basis sets. For the exchange-correlation term, the PBE form was used^54^. For the van der Waals interactions, the DFT-D2 method was applied^55^. The cut-off energies for the wavefunctions and charge density were 56 Ry and 506 Ry, respectively. The number of *k* points sampled in the Brillouin zone was more than 5 × 5 per the surface unit cell of c-plane sapphire. All the models were optimized to meet the force criterion of 0.02 eV/Å. Sapphire slab employed here consists of six Al_2_O_3_ layers and the thickness of a vacuum region is 0.9 nm. The top (bottom) layer of a sapphire slab is terminated with only one Al atom per surface unit, to make its electronic states semiconducting. The calculated lattice constant for the hexagonal MoS_2_ monolayer is 0.318 nm, while that for sapphire is 0.4798 nm. In this study, the change in the lattice mismatch of MoS_2_ grown on sapphire is quite essential to see the stability of MoS_2_/sapphire heterostructures. Thus, the lattice constant for sapphire is set to 0.4789 nm to follow the experimental ratio of 1.506 for MoS_2_ and sapphire. This treatment is justified, because there is no chemical bond between MoS_2_ `monolayer and sapphire slab, and the lattice constant of sapphire slab is not affected by MoS_2_. Adsorption energy, Ead is defined as *E*_ad_ = (*E*^MoS2/sapphire^ − (*E*^MoS2^ + *E*^sapphire^))/S, where *E*^MoS2^, *E*^sapphire^, and *E*^MoS2/sapphire^ are the calculated total energies for MoS_2_ monolayer, sapphire, and MoS_2_/sapphire. S is the area of a superstructure.

### Device fabrication and transport characterization

To evaluate the transport properties of monolayer MoS_2_ grown by MOCVD, it was transferred onto a 90 nm SiO_2_/*n*^+^-Si substrate using a standard transfer technique (Supplementary Fig. 25). Polymethyl methacrylate (PMMA, MicroChem, 495k) was spin-coated on the MoS_2_/sapphire wafer and further supported by thermal release tape (TRT, Nitto, Revalpha). The TRT/PMMA/MoS_2_ stack was released from the sapphire wafer in a KOH solution (1 mol/L) and rinsed with deionized water. Following this, the stack was transferred to the SiO_2_/Si substates, and the TRT/PMMA film was removed in acetone. For device fabrication, a maskless aligner μMLA (Heidelberg instrument) with double layer resists (PMGI SF5/AZ1500) and NMD-3 developer was used to define channel and electrode patterns. Then, the shape of MoS_2_ channels were defined by CF_4_ plasma etching. A 1-nm/30-nm Ni/Au electrode was deposited in an UHV chamber at a base pressure of ~5×10^-8 ^Pa with a low deposition rate of 0.0014 Å/s for Ni and 0.15 Å/s for Au. The sample temperature was maintained at 15 °C in the UHV chamber to avoid thermal damage. All electrical measurements in this study were conducted using a vacuum prober with a cryogenic system, employing a Keysight B1500 semiconductor parameter analyzer.

## Supplementary information

Supplementary Information

Transparent Peer Review file

## Data Availability

Relevant data supporting the key findings of this study are available within the article and the Supplementary Information file. All raw data generated during the current study are available from the corresponding authors upon request.
