# Supplementary material for: Self-aligned and self-limiting van der Waals epitaxy of monolayer MoS2 for scalable 2D electronics
Source: Nat Commun. 2026 Jan 21;17:602. doi: 10.1038/s41467-026-68320-8 (PMC12824160; doi:10.1038/s41467-026-68320-8)
Supplement: Supplementary file 1 — Supplementary Information [file 41467_2026_68320_MOESM1_ESM.pdf]

Supplementary information

Self-aligned and self-limiting van der Waals epitaxy of monolayer MoS_2 for scalable 2D electronics

Yoshiki Sakuma[1*], Keisuke Atsumi[2], Takanobu Hiroto[3], Jun Nara[4], Akihiro Ohtake[1], Yuki Ono[5], Takashi Matsumoto[5], Yukihiro Muta[5], Kai Takeda[5], Emi Kano[6], Toshiki Yasuno[6], Xu Yang[6], Nobuyuki Ikarashi[6], Asato Suzuki[7], Michio Ikezawa[7], Shuhong Li[2], Tomonori Nishimura[2], Kaito Kanahashi[2], Kosuke Nagashio[2**]

[1]Research Center for Electronic and Optical Materials, National Institute for Materials Science, 1-1 Namiki, Tsukuba 305-0044, JAPAN
[2]Department of Materials Engineering, The University of Tokyo, 7-3-1 Hongo, Bunkyo-ku, Tokyo 113-8656, Japan
[3]Research Network and Facility Services Division, National Institute for Materials Science, 1-2-1 Sengen, Tsukuba 305-0047, Japan
[4]Research Center for Materials Nanoarchitectonics, National Institute for Materials Science, 1-1 Namiki, Tsukuba 305-0044, JAPAN
[5]Tokyo Electron Technology Solutions Limited, 650 Mitsuzawa, Hosaka-cho, Nirasaki, 407-0192, Japan
[6]Institute of Materials and Systems for Sustainability, Nagoya University, Furo-cho, Chikusa-ku, Nagoya, 464-8601, Japan
[7]Department of Physics, University of Tsukuba, 1-1-1 Tennodai, Tsukuba, 305-8571, Japan

*sakuma.yoshiki@nims.go.jp, **nagashio@material.t.u-tokyo.ac.jp

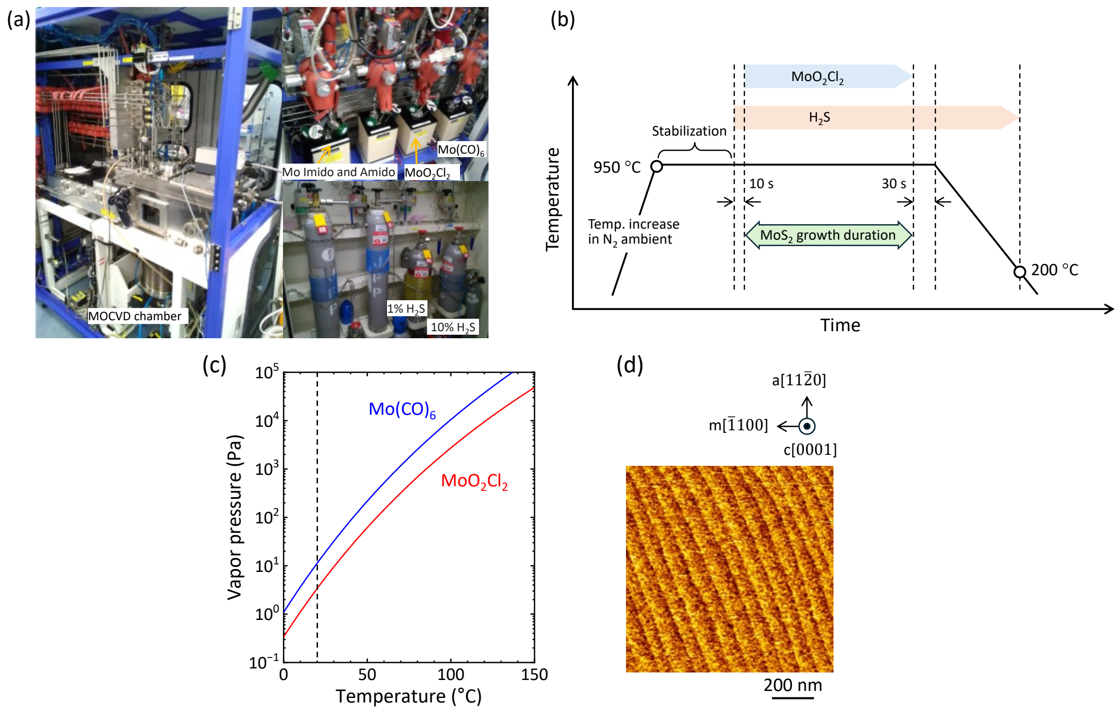

Supplementary Figure 1 (a) Photograph of a horizontal cold-wall MOCVD reactor with the carbon-free MoO_2Cl_2 and H_2S gas sources. Although, MoO_2Cl_2 is classified into inorganics to be precise, not organometals, this precursor can be used with the same way in conventional MOCVD growth system. Therefore, for simplicity, we call our synthetic method MOCVD in this report. (b) The detailed description of the MOCVD process steps in the case of growth at 950 °C using MoO_2Cl_2 as Mo precursor. The Mo supply is intentionally terminated 30 seconds before the onset of wafer cooling. This is a key advantage of MOCVD, where the timing of precursor flow can be precisely controlled by computer-operated pneumatic valves. After stopping the Mo precursor, H_2S continues to flow until the substrate temperature decreases to 200 °C, which serves the purpose of preventing the thermal decomposition of MoS_2 and healing sulfur vacancies. This protocol effectively prevents additional nucleation or growth during the cooling stage, since Mo supply has already been cut off. (c) Vapor pressures of MoO_2Cl_2 and $Mo(CO)_6$ as a function of temperature. (d) AFM image of pretreated sapphire surface with step and terrace structure.

Supplementary Note: Thermal stability, in particular, disproportionate decomposition, and volatility of several kinds of molybdenum oxychlorides are described in the literatures.[1,2] MoO_2Cl_2 is the most stable and has higher sublimation pressure among them.[3] According to Saeki *et al.*, the sublimation pressure is given by equation (1) at the temperature range of 50-157 °C.[2] In contrast, Ohta et al. reported the sublimation from solid $Mo(CO)_6$ as equation (2) at the range of −10.5-23.1 °C.[4] In (b), to compare the sublimation property between two precursors, we plotted the pressures by extrapolating the temperature range to 0 °C-150 °C.

$$\mathrm{Log_{10}}\ P\ (\mathrm{mmHg}) = -3830/T\ (\mathrm{K}) + 11.747 \qquad (1)$$

$$\mathrm{Log_{10}}\ P\ (\mathrm{Pa}) = 14.9 - 4058/T\ (\mathrm{K}) \qquad (2)$$

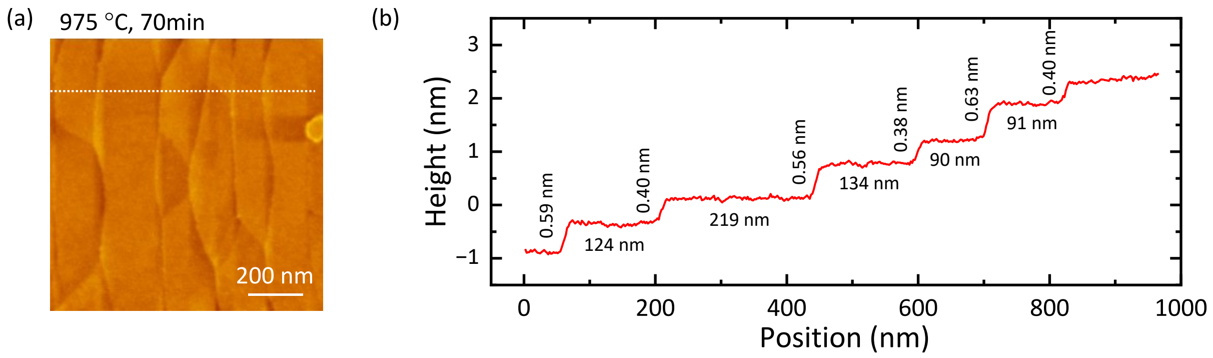

Supplementary Figure 2 (a) AFM image of monolayer MoS_2 grown on the sapphire substrate at 975 °C for 70 min. (b) Height profile along the white line in (a). For c-plane sapphire wafer with c/m = −0.2° of miscut angle, the single step height and the terrace width for 1 ML height are c/6 = 0.217 nm and approximately 62 nm, respectively. According to the height profile along the white line, several kinds of terrace widths appear depending on the step heights. Therefore, when the growth temperature is higher than 950 °C, the terrace width increased to ~200 nm with 3ML step height.

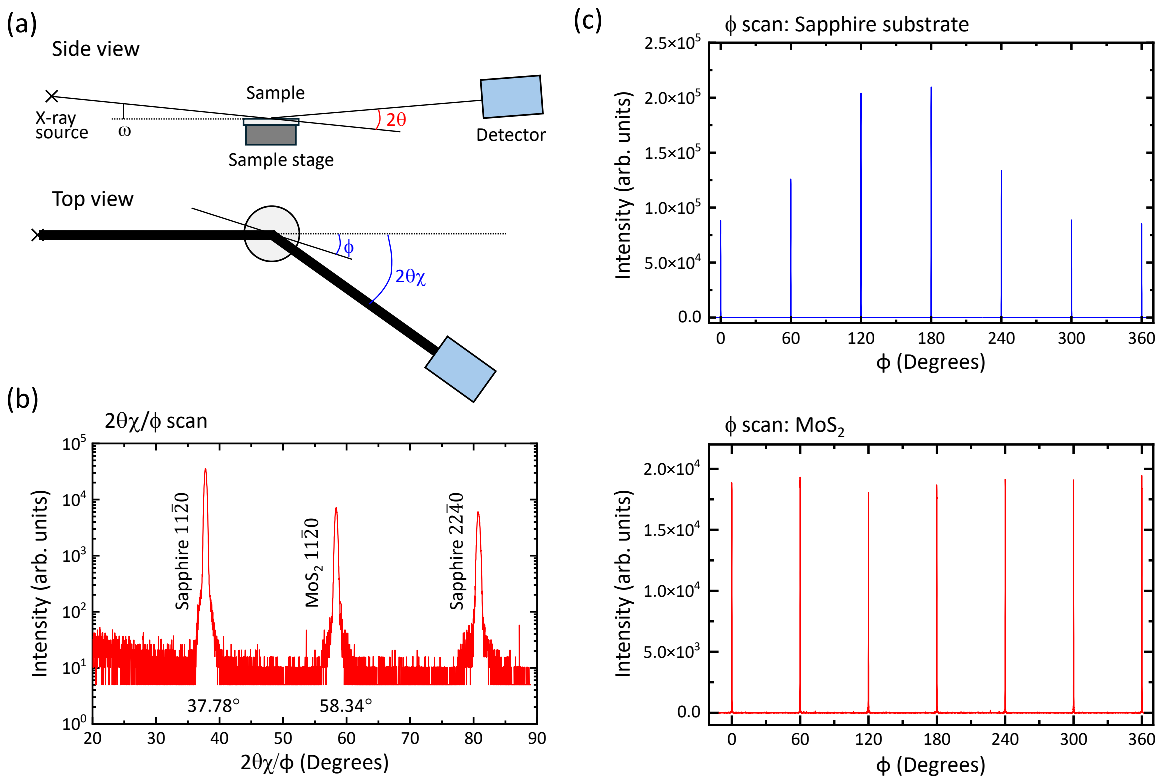

Supplementary Figure 3 (a) In-plane XRD measurement setup for 2θχ/ϕ scan and ϕ scan. Top view shows the situation where reflection from targeted {11$\bar{2}$0} plane is measured. (b) 2θχ/ϕ scan for MoS_2 on the sapphire substrate. (c) In-plane XRD azimuthal angle (ϕ) scans of {11$\bar{2}$0} planes for sapphire substrate (α-Al_2O_3) and monolayer MoS_2, respectively.

Supplementary Note: The basic procedures to make in-plane XRD measurements using Rigaku SmartLab diffractometer was described in the Method section.[5] As shown in (b), the diffraction peak corresponding to the {11$\bar{2}$0} plane of MoS_2 was observed to be parallel to that of the sapphire substrate, i.e., [11$\bar{2}$0]MoS_2//[11$\bar{2}$0]α-Al_2O_3. At fixed 2θχ angles of 37.78° and 58.34°, azimuthal ϕ scans of {11$\bar{2}$0} planes in both sapphire substrate and MoS_2 were conducted separately. The ϕ scan was conducted over 0°-360° under faster scan rate of 20°/min. In some cases, however, the ϕ range was narrowed to 0°-180° for a high-precision slow scan of 0.3°/min to shorten the measurement time. The peak resolution of the in-plane ϕ-scan is approximately ~0.2°, which was confirmed by the sapphire 11$\bar{2}$0 substrate peak. As clearly seen in (c), the strong diffraction peaks reflected from the {11$\bar{2}$0} planes of MoS_2 appeared with 60° intervals. This indicates that monolayer MoS_2 film contains negligible twisted MoS_2 domains and shows a high-quality in-plane epitaxial alignment with sapphire substrate. It should be noted that the origin of the ϕ coordinates shown in (c) were intentionally shifted, for clarity, from the absolute values obtained by 2θχ/ϕ scans for MoS_2 and sapphire, with considering the offset angle of ϕ between MoS_2 and sapphire of 10.28° = (58.34°−37.78°)/2.

(a)

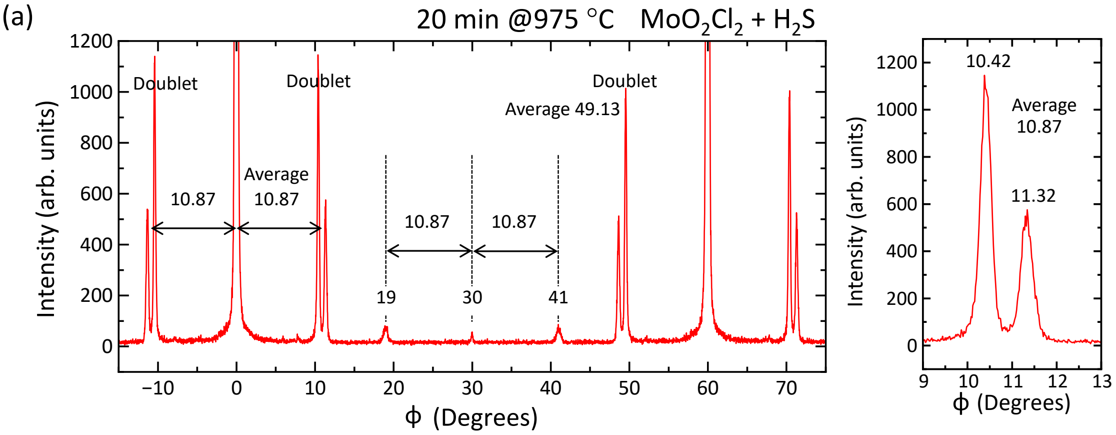

(b)

Sapphire (4.759 Å)		MoS$_2$ (3.161 Å)		Super cell	
Notation	Cell size (Å)	Notation	Cell size (Å)	Mismatch (%)	Twist angle (°)
$\sqrt{3} \times \sqrt{3}$ R30°	8.24	$\sqrt{7} \times \sqrt{7}$ R19.11°	8.36	+1.461	10.89
2×2 R0°	9.52	3×3 R0°	9.48	-0.368	0
$\sqrt{7} \times \sqrt{7}$ R19.11°	12.59	4×4 R0°	12.64	+0.420	19.11 (=30-10.89)
3×3 R0°	14.28	$\sqrt{19} \times \sqrt{19}$ R23.41°	13.78	-3.492	23.41
3×3 R0°	14.28	$\sqrt{21} \times \sqrt{21}$ R10.89°	14.49	+1.461	10.89
$2\sqrt{3} \times 2\sqrt{3}$ R30°	16.49	5×5 R0°	15.81	-4.129	30
$2\sqrt{3} \times 2\sqrt{3}$ R30°	16.49	$3\sqrt{3} \times 3\sqrt{3}$ R30°	16.43	-0.368	0
$2\sqrt{3} \times 2\sqrt{3}$ R30°	16.49	$2\sqrt{7} \times 2\sqrt{7}$ R19.11°	16.73	+1.461	10.89
$\sqrt{13} \times \sqrt{13}$ R13.9°	17.16	$3\sqrt{3} \times 3\sqrt{3}$ R30°	16.43	-4.276	16.10

(c)

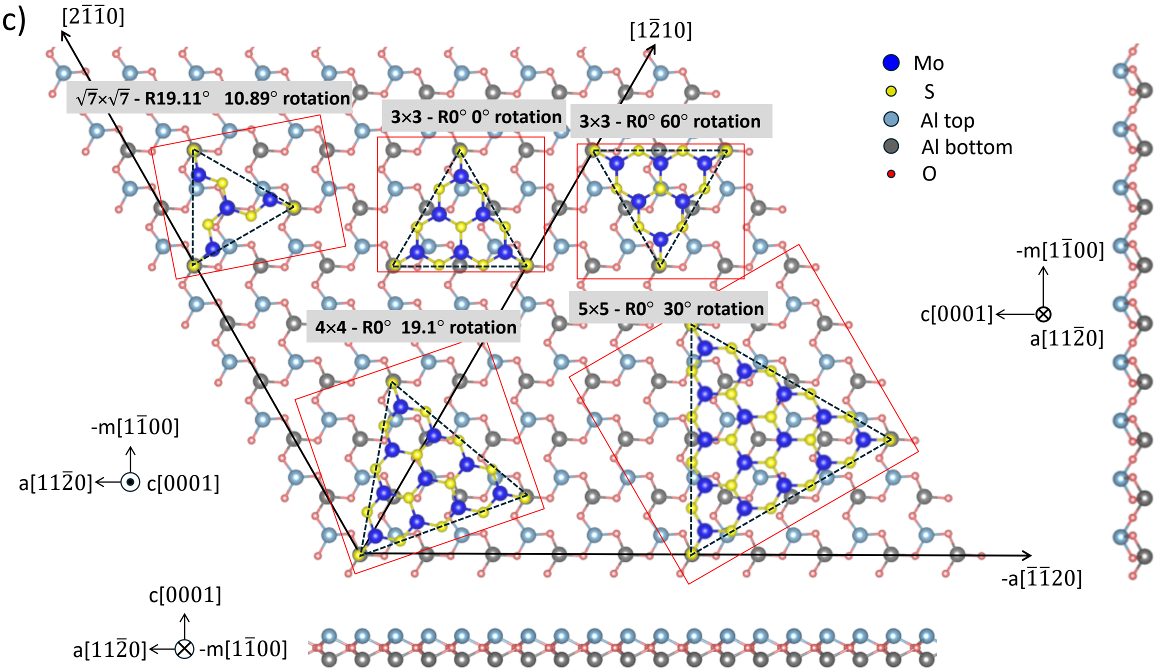

Supplementary Figure 4 (a) In-plane XRD azimuthal angle (ϕ) scans of {11$\bar{2}$0} planes for MoS_2 grown at 975 °C for 20 min with a step width of 0.02° and a scan rate of 0.3°/min. In the right figure, the peaks around 10.87° were magnified. The observed azimuthal angles indicate the relative in-plane twisted angles between the (11$\bar{2}$0) plane of MoS_2 and the (11$\bar{2}$0) plane of c-plane sapphire. (b) Supercell relations of the MoS_2 and sapphire substrate.[6] For simplicity, nine pairs with a twisted angles between 0° and 30° as well as a lattice mismatch of less than

5 % have been listed. The domains with twisted angles shown in red were experimentally confirmed. (c) Schematic illustrations for MoS_2 domains with 4 sets of supercell relations which was experimentally observed in (a), plus 60° antiparallel domain. To highlight the three-fold symmetry of the sapphire substrate with single-layer Al top surface, top and bottom Al atoms were colored differently, as shown in the side view of right figure. It should be noted that (c) presents only several representative examples and does not include all the allowed atomic models. Notably, the mirror image domains with respect to a-axis and m-axis are not shown here, other than the 60° antiparallel domain. Furthermore, we have not considered the exact overlaid structures of MoS_2 domains with the sapphire surface. Determining the most energetically stable configuration for each domain is beyond the scope of this paper.

Supplementary Note: In thermal statistical distributions, the occupation probability of the lowest energy state does not reach unity; rather, it follows the Maxwell–Boltzmann distribution, whereby energetically stable orientations exhibit higher occupation probabilities. This situation is illustrated in the potential energy landscape in the following figure (d), in which multiple local minima exist as a function of twisted angle, separated by energy barriers. The following table (e) presents the calculated strain induced by lattice mismatch and cell size as a function of twist angle through a supercell model. In addition, we calculated the volume fraction for each orientation from peak area intensities obtained by in-plane XRD. Basically, orientations with smaller lattice mismatch exhibit larger volume fractions, whereas energetically unfavorable orientation appear with very small volume fractions. Importantly, in the initial stage of nucleation, it is considered that nuclei with the minimum cell size of 10.89° first determine the epitaxial relationship. Although this configuration is energetically unfavorable, the experimental observation that 10.89° grains remain in sufficient quantity even after 20 min of film growth suggests that they do not transition to the most stable 0° domain by overcoming the energy barrier. This is attributable to the fact that the energy barrier increases as the nucleus size becomes larger. Consequently, the relationship between volume fraction and energy does not follow a simple proportionality. We note that such misoriented domains, while less frequently emphasized in the literature, have been observed experimentally in earlier XRD and TEM studies, as well as optical and AFM studies.[5–8]

(d)

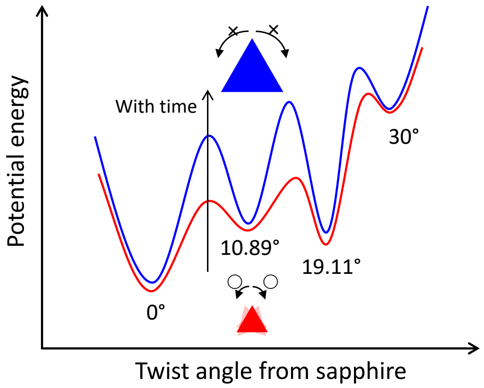

(e)

Super cell			
Twist angle (°)	Mismatch (%)	Cell size (Å)	Volume fraction (%)
0	-0.368	9.48	81.9
10.89	+1.461	8.24	17.3
19.11	+0.420	12.64	0.60
23.41	-3.492	13.78	NA
30	-4.129	15.81	0.17
16.10	-4.276	16.43	NA

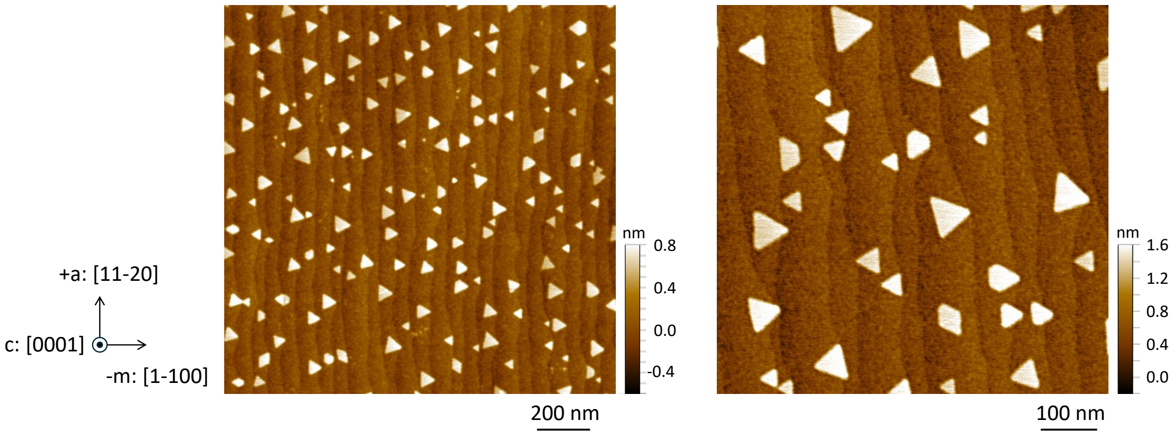

Supplementary Figure 5 AFM images of monolayer MoS_2 grown on the sapphire substrate with miscut angle of c/m = −0.2° at 975 °C for 4 min.

Supplementary Note: To address whether the nucleation mainly occurs at step edges or terraces, AFM observations were performed at the initial growth stage. It is evident that MoS_2 nuclei can form at various locations, such as the center of terraces, along step edges, or near the upper edges of steps, without any consistent positional preference. At the initial stage of growth (4 min), the number of 0° domains with vertices pointing toward the –m direction is larger than that of 60° domains with vertices pointing toward the +m direction. Furthermore, domains rotated by approximately ±10.87° relative to 0°and 60°are observed, which is in good agreement with the 20-min in-plane XRD data (Supplementary Figure 4). Furthermore, in contrast to the previous report,[9] we observed that 0° and 60° domains can coexist within terraces of the same height. These results clearly indicate that orientation control in the present growth process is not governed by step edges and/or difference in slab A and slab B.

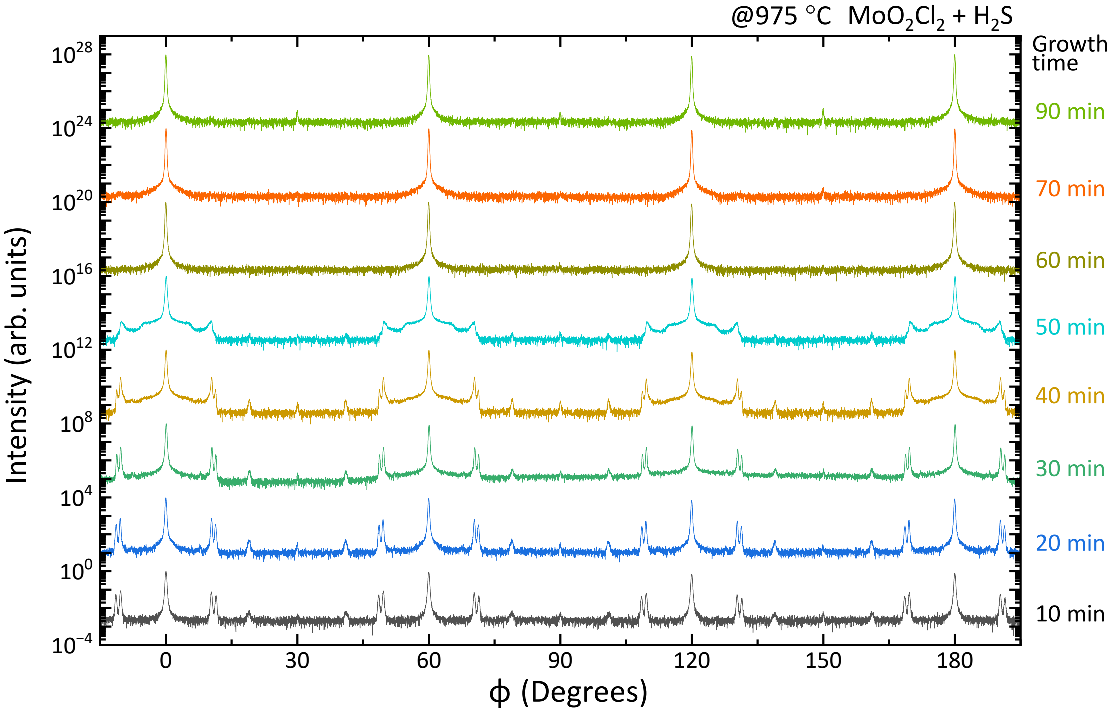

Supplementary Figure 6 In-plane XRD azimuthal angle (ϕ) scans of $\{11\bar{2}0\}$ planes for MoS_2 grown at 975 °C for various growth durations, ranging from 10 min to 90 min.

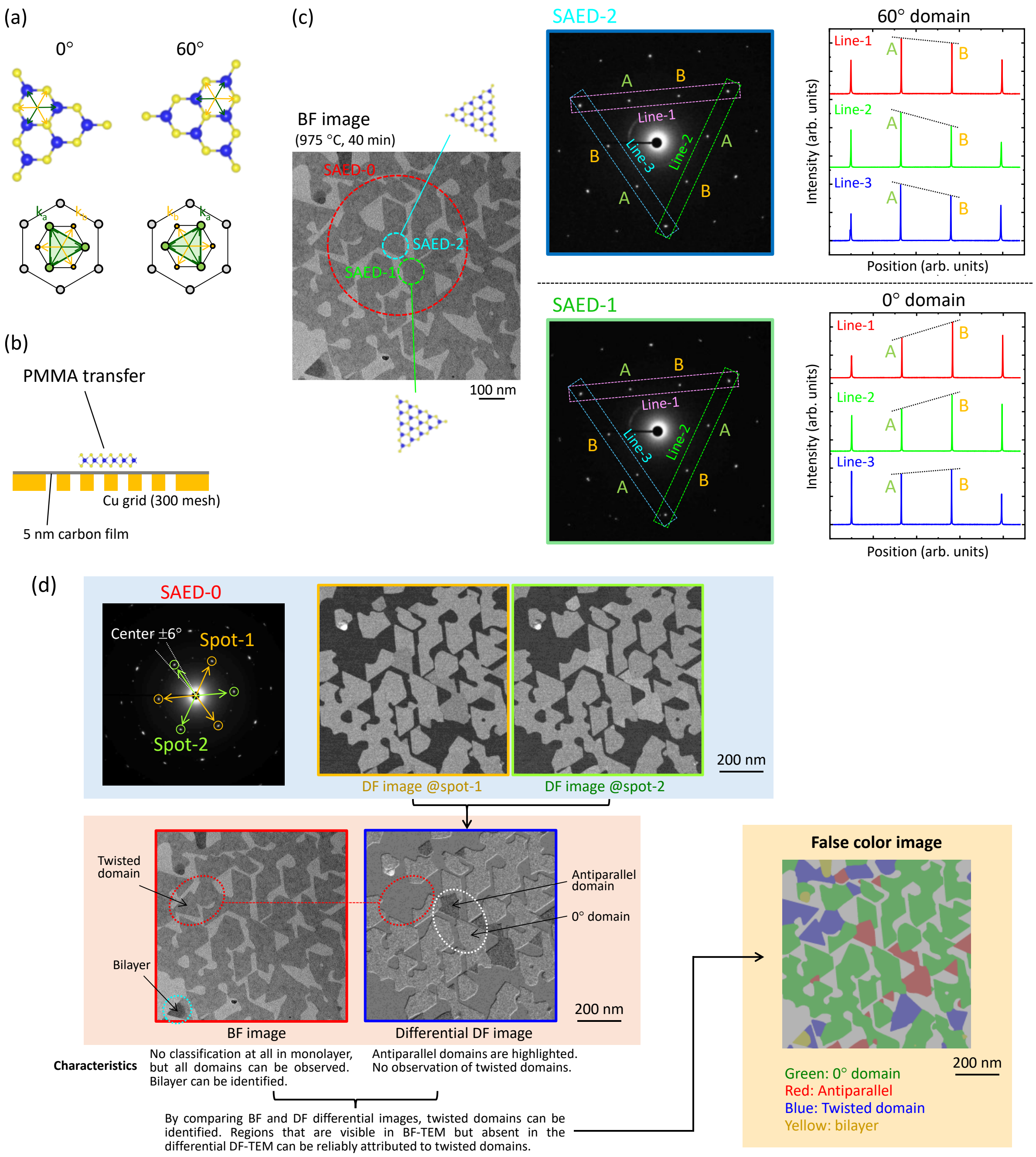

Supplementary Figure 7 (a) Atomic configurations of 0° and 60° triangular domains with sulfur zigzag edges (S_{zz}) in monolayer MoS_2 and their corresponding electron diffraction patterns. Theoretically, the diffraction spot intensities associated with the reciprocal lattice vectors $\mathbf{k}_a$ are consistently more pronounced than those corresponding to $\mathbf{k}_b$ due to the violation of Friedel's law.[10] (b) Schematic representation of the sample preparation procedure employed for differential DF-TEM analysis. Monolayer MoS_2 was transferred from a sapphire substrate to a TEM grid with 5-nm amorphous carbon film via the PMMA-assisted wet transfer method. (c) Schematic illustration of the methodology for distinguishing 0° and 60° antiparallel domains based on the violation of Friedel's law. MoS_2 sample grown at 975 °C for 40 min was selected as an example. Following the acquisition of a BF-TEM image, selected-area electron diffraction (SAED) patterns were recorded at two positions, denoted SAED-1 and SAED-2. Electron

diffraction intensities were subsequently obtained along three lines (line-1 to line-3). It is evident that the intensities at spots B(A) are always higher than A(B) at SAED-1(2), confirming the violation of Freidel law. It should be noted that, by considering the shape of triangular domains in BF-image, this relationship is the opposite to the theoretical prediction shown in (a). This might be an issue arising from the optical system in TEM, such as the camera settings. Therefore, we carried out these experiments while checking the relationship between the direction of three triangles in the employed TEM system: 1) the triangular pattern formed by connecting the bright diffraction spots (A or B), 2) the geometric direction of the triangular region in DF-image, and 3) the triangle of the Mo sublattice (requiring observations by atomic resolution HAADF-STEM). Consequently, it is also verified that the triangular domains have S_{zz} edges as illustrated in (a) in our typical growth conditions. (d) Schematic illustration of the differential DF-TEM strategy employed to construct false-color domain maps. Initially, SAED was acquired over a relatively wide area (SAED-0 in (c)). Since 0° and 60° antiparallel domains are always dominant at any area on the sample, spot-1 and spot-2 with the relation of 180° inversion can be selected, thereby violating Friedel's law. Therefore, two sets of DF-TEM images were acquired at diffraction spots 1 and 2 using a minimal objective aperture (±6°). In the differential DF-TEM image, 0° and 60° antiparallel domains are clearly distinguished because the contrast is doubly emphasized. Note that only domains with twist angles within ±6° are identified as 0°/60° domains due to the finite mechanical aperture size in this method. Conversely, domains where the diffraction spot is outside the objective aperture are not observed in the dark-field image, which can be judged as rotating domains. Subsequently, a BF-TEM image was obtained, where all domains are visible. By comparing the differential DF-TEM image with the BF-TEM image, the regions where no domains are observed in differential DF-TEM but are visible in BF-TEM can be recognized as twisted domains. Finally, this approach enables the construction of a comprehensive false-color domain map for monolayer MoS_2.

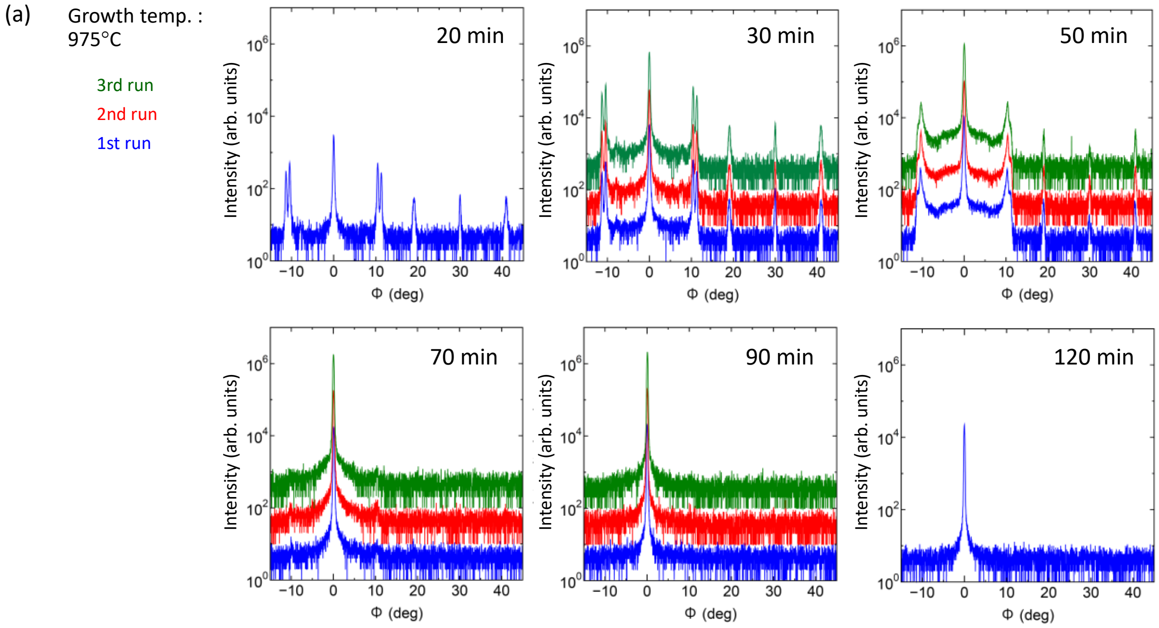

(b)

Growth time (min)	Runs	0° peak integral intensity (counts·deg)	Averaged integral intensity (counts·deg)	Standard deviation (counts·deg)	Coefficient of variation (%)
30	#1	1831	1814.66	94.20	**5.19**
	#2	1692			
	#3	1921			
50	#1	3570	3584.00	214.15	**5.98**
	#2	3329			
	#3	3853			
70	#1	4577	4729.67	108.15	**2.29**
	#2	4798			
	#3	4814			
90	#1	5139	5071.67	53.92	**1.06**
	#2	5007			
	#3	5069			

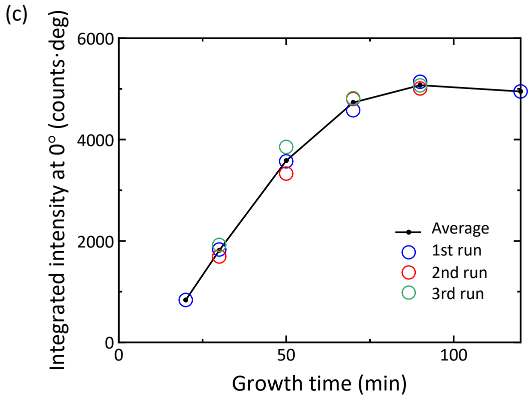

Supplementary Figure 8 (a) In-plane XRD data for samples grown at 975 °C for different growth times. (b) Integrated intensity data of the 0° peak, standard deviation, and coefficient of variation (CV) obtained from in-plane XRD data. (c) Integrated intensity at 0° peak as a function of growth time for all growth runs.

Supplementary Note: To demonstrate the high reproducibility of the self-aligned coalescence process in our MOCVD system, we conducted three consecutive sets of MOCVD runs with four different growth durations: 30 min (just before starting partial coalescence), 50 min (during self-aligned coalescence), 70 min (after completing coalescence, #1), and 90 min (after completing coalescence, #2) as shown in (a). In addition, to confirm the quantitative accuracy of the XRD peak intensity, another independent run of 20 min and 120 min were performed, and the integrated intensity of the 0° peak of all 14 samples was evaluated to investigate the self-limiting nature of MoS_2 growth. In order to shorten the measurement time, the scan was limited to one cycle of 60° (from −15° to +45°) and increased the scan speed to 1.2°/min, which is four times faster than 0.3°/min used in other measurements. Although this resulted in a slightly lower signal-to-noise ratio, the accuracy is sufficient for reproducibility evaluation. The

raw in-plane XRD data from each run show very similar peak shapes and intensities, confirming high reproducibility at the qualitative level. For quantitative comparison, we calculated the integrated intensity of the 0° peak from each dataset, as shown in (b). As a measure of variability, we used the coefficient of variation (CV = standard deviation / mean × 100 %). The CV values are sufficiently small throughout the growth series, and become even smaller after the coalescence is complete (70 min and 90 min), reflecting improved uniformity. Moreover, the self-limiting behavior can be seen in (c), suggesting the high quantitative accuracy of in-plane XRD.

(a)

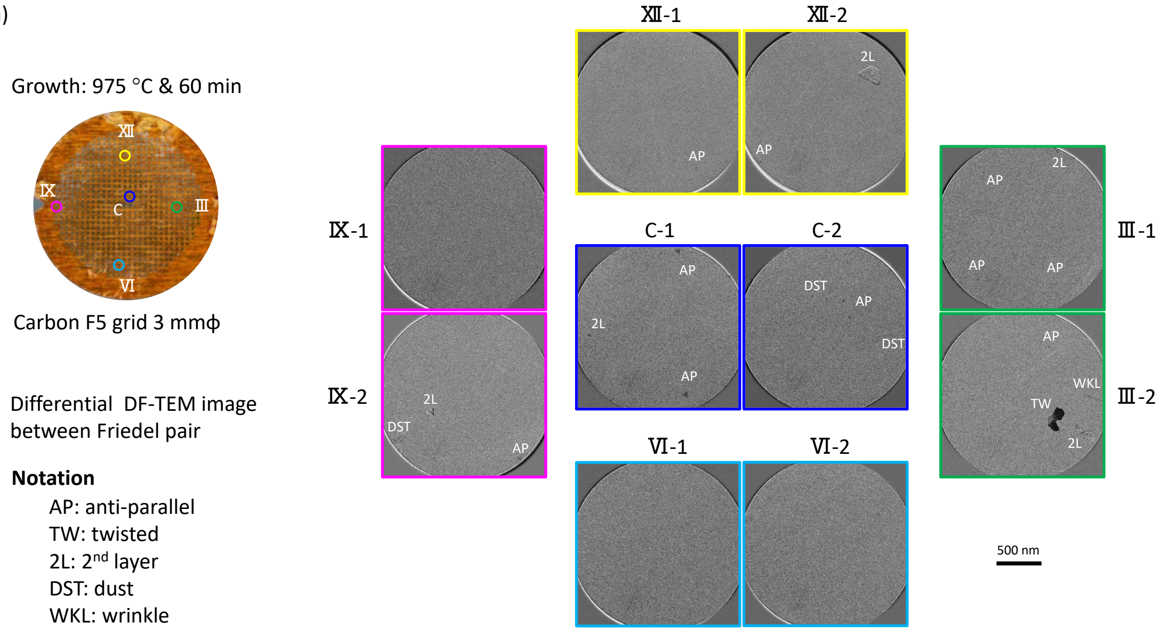
Growth: 975 °C & 60 min
Ⅻ
Ⅸ
C
Ⅲ
Ⅵ
Carbon F5 grid 3 mmϕ
Differential DF-TEM image between Friedel pair
Notation
AP: anti-parallel
TW: twisted
2L: 2nd layer
DST: dust
WKL: wrinkle
Ⅻ-1
Ⅻ-2
Ⅸ-1
Ⅸ-2
C-1
C-2
Ⅲ-1
Ⅲ-2
Ⅵ-1
Ⅵ-2
AP
2L
DST
TW
WKL
500 nm

(b)

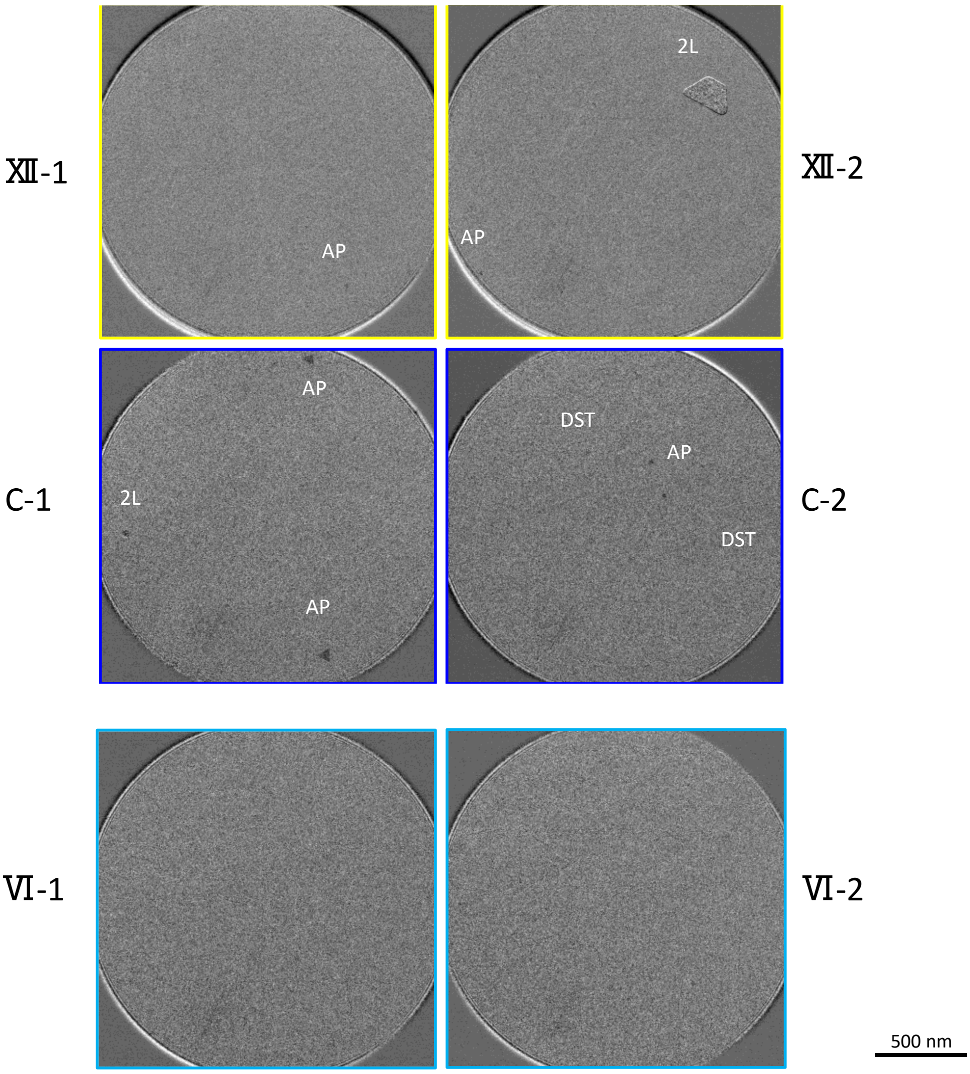
Ⅻ-1
Ⅻ-2
C-1
C-2
Ⅵ-1
Ⅵ-2
AP
2L
DST
500 nm

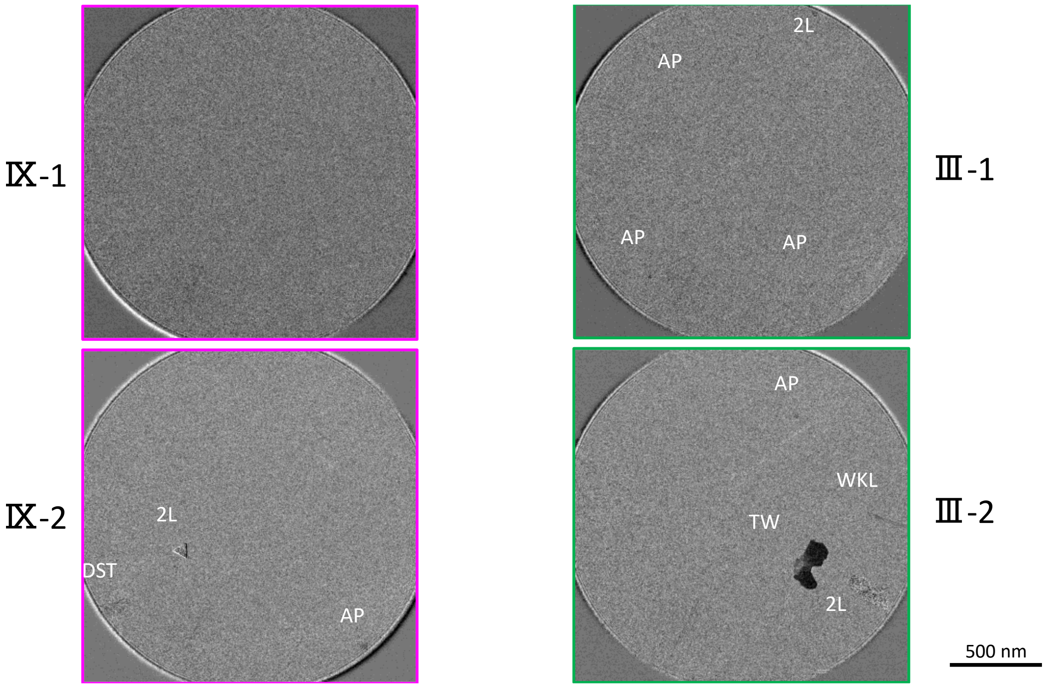

Supplementary Figure 9 (a) Photograph of a TEM grid onto which monolayer MoS_2, grown at 975 °C for 60 min, was transferred. Ten differential DF-TEM images obtained from a Freidel pair are shown in grayscale without false coloring (see Supplementary Figure 7d for details). The corresponding locations are indicated in the TEM grid photograph. It should be emphasized that the circular field of view in DF-TEM is restricted because the smallest objective aperture was employed to improve the detection limit of domain rotation angles. Therefore, the edge of this aperture is visible in the images. (b) Magnified images.

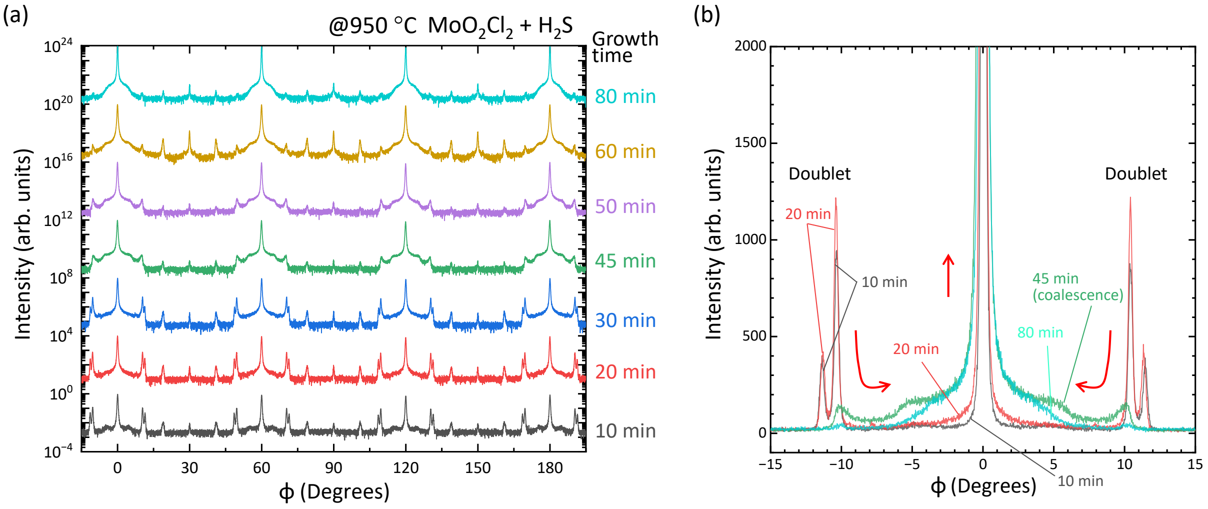

Supplementary Figure 10 In-plane XRD azimuthal angle (ϕ) scans of $\{11\bar{2}0\}$ planes for MoS_2 grown at 950 °C for various growth durations, ranging from 10 min to 80 min. (a) Full scan range data and (b) superimposed data focusing on the 0° domains with the doublet subpeaks at 10.4° and 11.3°.

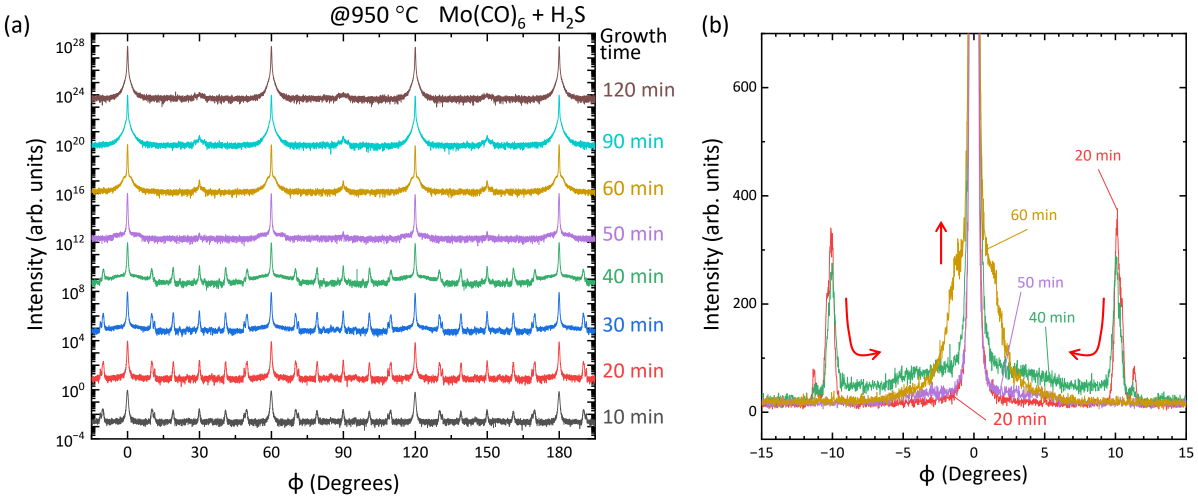

Supplementary Figure 11 In-plane XRD azimuthal angle (ϕ) scans of $\{11\bar{2}0\}$ planes for MoS_2 grown at 950 °C for various growth durations, ranging from 10 min to 120 min. The precursors used in this experiment are $Mo(CO)_6$ and H_2S, which are common in previous literature. (a) Full scan range data and (b) superimposed data focusing on the 0° domains with the doublet subpeaks at 10.4° and 11.3°. These results imply that the formation of single crystals via self-aligned coalescence is not exclusive to the MoO_2Cl_2 precursor employed in this study.

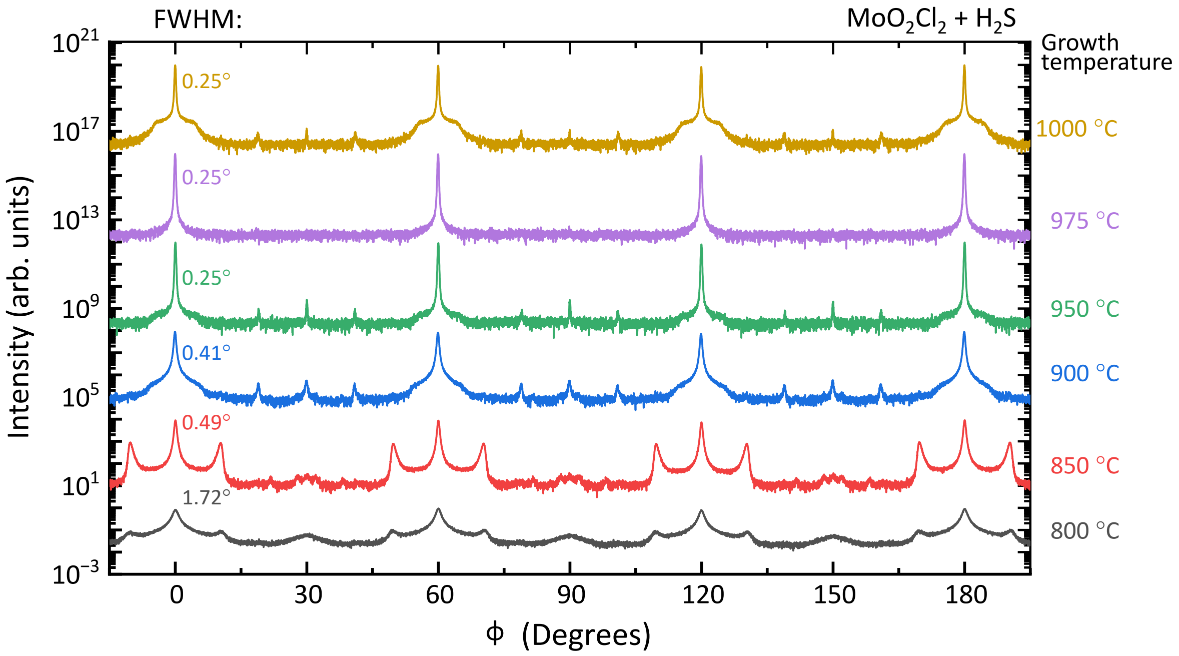

Supplementary Figure 12 In-plane XRD azimuthal angle (ϕ) scans of {11$\bar{2}$0} planes for MoS_2 grown for 60 min at various growth temperatures, ranging from 800 °C to 1000 °C. It should be noted that below 900 °C, the main peak intensity decreases, and the half-width broadens, indicating the degradation of crystallinity and increased in-plane mosaicity. Notably, at 850 °C and 800 °C, the peak originating from the 11° rotated domain remains even after full coverage, suggesting that the self-aligned coalescence does not proceed efficiently. Moreover, the material characterization was performed on 2×2 cm^2 sapphire substrates, whereas device fabrication utilized 2-inch sapphire wafers. This difference in wafer size necessitated distinct MOCVD conditions for optimization. Specifically, the 2-inch wafers required a substrate temperature approximately 50 °C higher than that used for the smaller substrates due to the different susceptors employed in the MOCVD system. For the 2×2 cm^2 wafers, a nominal growth temperature of 1000 °C was applied. However, this temperature was excessively high for sapphire substrates of that size, causing surface roughening. This subtle variation was not readily discernible in optical images. Instead, AFM image of MoS_2 grown at 1000 °C for 60 min on sapphire substrates is shown below. This image clearly show that monolayer MoS_2 did not fully cover the substrate surface under these high-temperature conditions, indicating incomplete self-aligned growth. This effect is evident in the broad tail structure at the 0° peak in the in-plane XRD for the sample at 1000 °C.

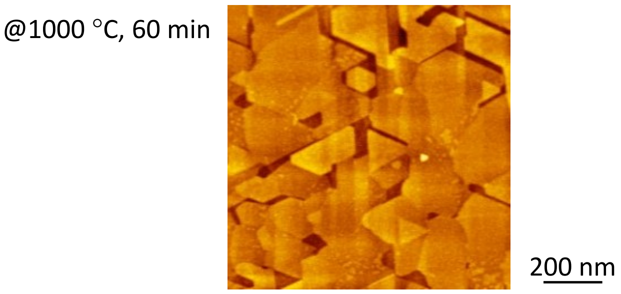

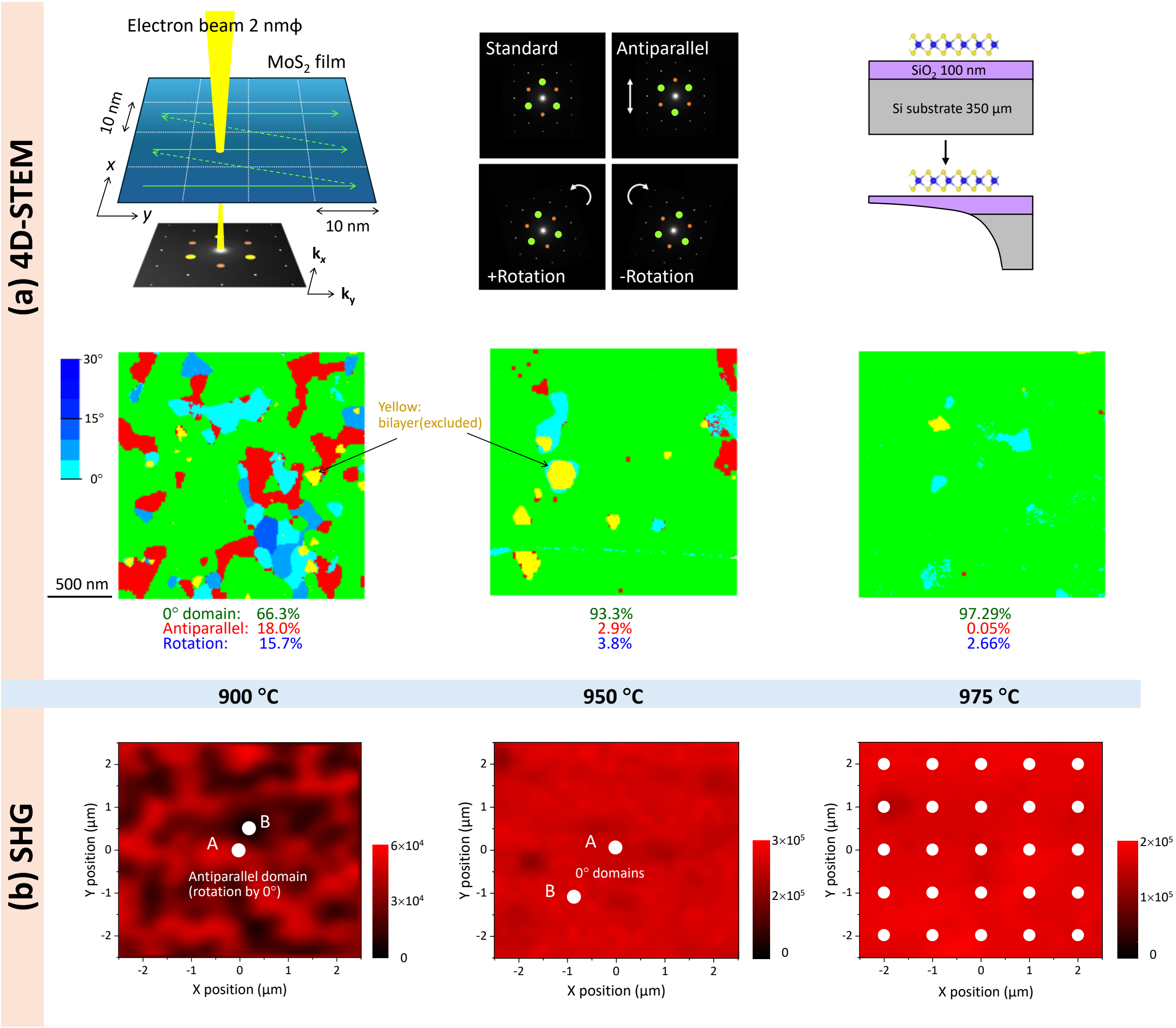

Supplementary Figure 13 Comparison between 4D-STEM and SHG results. (a) Four distinct types of diffraction patterns -standard (0°), antiparallel (60°), clockwise rotation, and anticlockwise rotation- can be identified through 4D-STEM measurements. 4D-STEM mappings for MoS_2 obtained at various growth temperatures. Schematic of the sample preparation for differential DF-TEM analysis is also shown. Monolayer MoS_2 was transferred from a sapphire substrate to a SiO_2/Si substrate using the PMMA wet transfer method. Subsequently, the backside Si was etched by ion milling, reducing the SiO_2 thickness much less than 50 nm to avoid electron scattering effect. (b) Schematic representation of the optical setup used for SHG measurements and SHG mapping images for MoS_2, obtained at various growth temperatures.

Supplementary Note:

4D-STEM: The 4D-STEM technique[8,11] enables the rapid acquisition of 2-nmϕ nanobeam electron diffraction patterns over a 2D array of spatial positions with a 10-nm pitch. Computational analysis of this dataset reveals structural variations on a pixel-by-pixel basis. For a fully continuous monolayer MoS_2 film grown at 900 °C for 60 min, the 0° domains,

represented in green, are dominant, while low-angle domains (blue) and antiparallel domains at 60° (red) coexist. Additionally, the yellow regions represent the bilayer, which is not included in the overall ratio calculation.

SHG: The optical setup was constructed for SHG measurements. Excitation light from a Ti:sapphire laser (wavelength: 810 nm, pulse width: ~100 fs, repetition rate: 82 MHz, intensity: 1 mW) focused onto the sample surface through an 80× objective lens. The light was focused onto the sample surface using an 80× objective lens. The generated SHG signal was collected through the same objective lens. The polarization of both the excitation and detected SHG light was controlled using a linear polarizer placed before the objective lens, either shared between excitation and detection paths or separately implemented on each side, in combination with half-wave plates. For polarization-resolved SHG measurements, the polarization elements were systematically rotated to analyze the SHG dependence under parallel polarization conditions.

SHG mapping images for MoS_2, obtained at various growth temperatures, were recorded at the polarization angle corresponding to the armchair direction. For the 850 °C sample (not shown here), where the armchair direction varies across domains, the polarization angle was set based on the armchair direction determined at a representative point. It should be noted that the maximum SHG intensities differ across all images. At 900 °C, positions A and B showed no rotational difference but demonstrated a notable intensity disparity, suggesting the presence of antiparallel domains. Although SHG is insensitive to antiparallel domains due to the six-fold pattern, the intensity is substantially suppressed at mirror twin boundary as well as tilted GBs.[12] At temperatures exceeding 950 °C, the absence of rotational differences and the uniform intensity levels indicate that all domains are well-aligned, which agrees well with the 4D-STEM results. For the 975 °C sample, the polarization dependent SHG patterns were taken at 25 different locations, as indicated by while circles. The data, shown in Figure 2e of the main text, clearly demonstrates the uniform orientation of MoS_2 at the wide area.

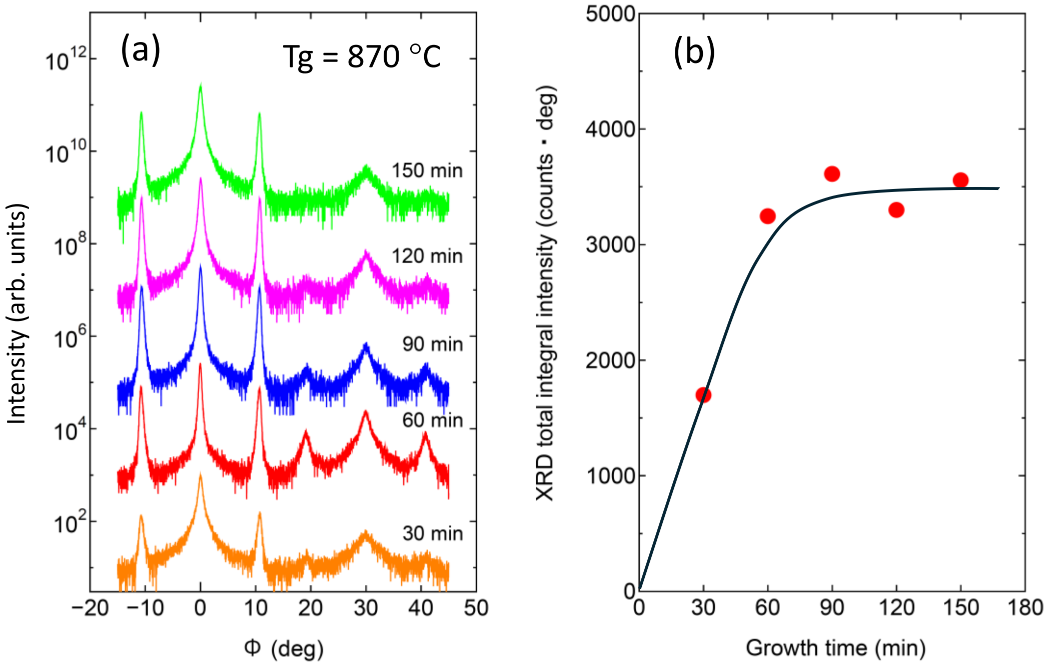

Supplementary Figure 14 (a) In-plane XRD azimuthal angle (ϕ) scans of $\{11\bar{2}0\}$ planes for MoS_2 grown at 870 °C for different growth times up to 150 min. (b) XRD total integral intensity between -15° and 45° for different growth times.

Supplementary Note: Here, longer growth time experiment was carried out to reveal whether single crystallization through grain boundary migration by self-aligned growth is possible or not at the low growth temperature of 870 °C. The monolayer MoS_2 was grown at 870 °C at different growth times up to 150 min. Then, in-plane XRD was carried out to extract the time evolution of self-aligned growth at the low temperature, as shown in (a). It is clear that single crystallization did not occur even at 150 min. Moreover, the XRD total integral intensity between -15° and 45° indicates that full coverage was reached at 60 min and the clear self-limiting behavior after 60 min. Although the single crystallization through self-aligned growth is ideally possible, it is not realistic experimentally because the kinetic rate constant follows an Arrhenius-type dependence ($\propto \exp(-Q/kT)$) and significantly longer growth times are required at lower temperatures to complete self-aligned growth.

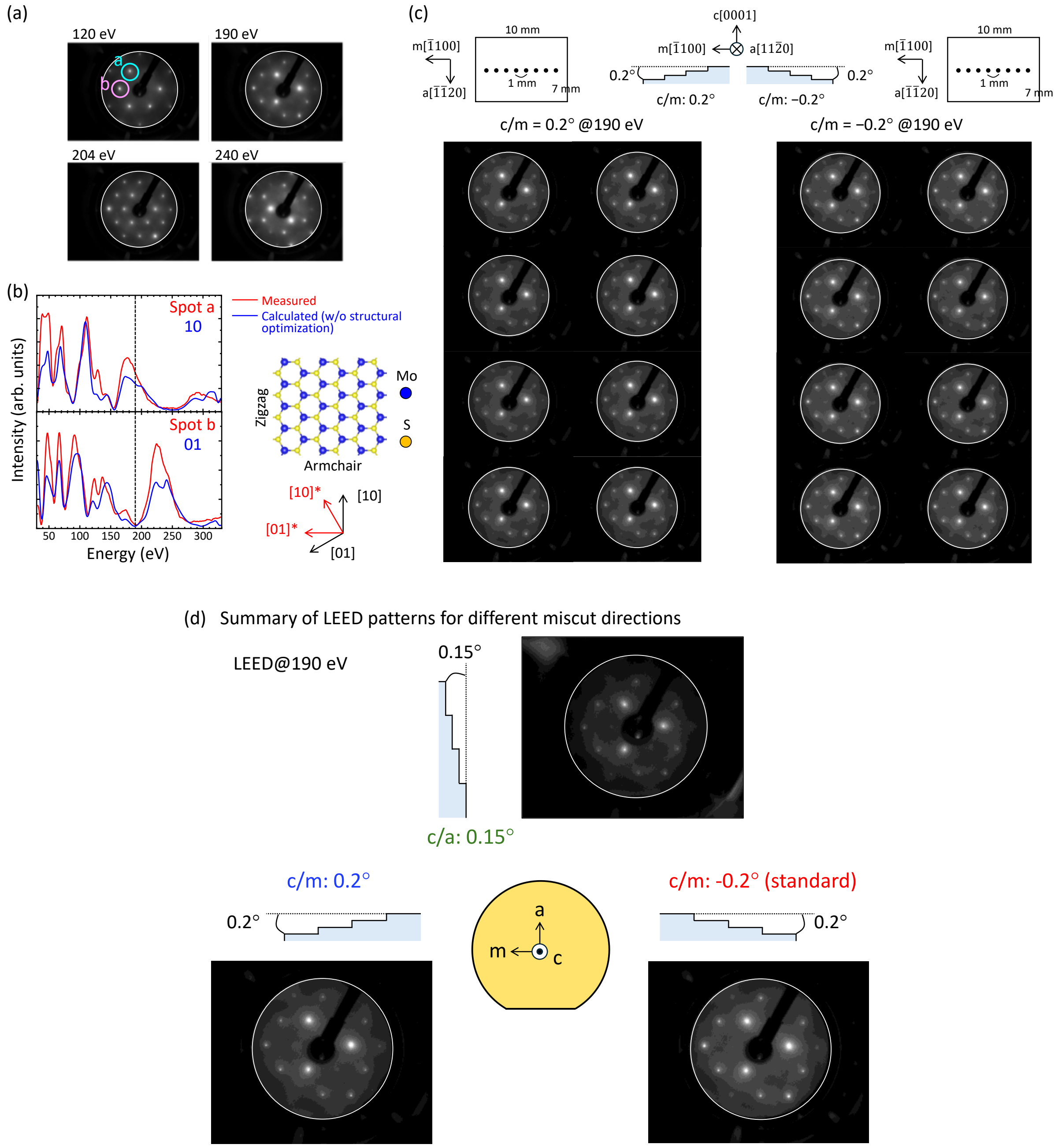

Supplementary Figure 15 (a) Representative LEED patterns of monolayer MoS_2 acquired at various electron energies for the miscut angle of c/m = −0.2°. Monolayer MoS_2 was grown on sapphire substate at 975 °C. (b) Experimentally obtained LEED *I*-*V* curves (red) for diffraction spots of “a” and “b” in (a) with simulated curves (blue) for diffraction spots of 10 and 01. (c) Schematic illustration of a monolayer MoS_2 film on a sapphire substrate where eight measurement points for LEED analysis are indicated. Two types of sapphire substrates with miscut angles of c/m = 0.2° and −0.2° were prepared for comparison. Eight LEED patterns are shown for each substrate. (d) Summary of LEED patterns of monolayer MoS_2 on sapphire substrates with different miscut directions.

Supplementary Note: Initially, LEED patterns of monolayer MoS_2 grown on sapphire substrates with a standard miscut angle of c/m = −0.2° were acquired at various electron beam energies, as shown in (a). Two representative diffraction spots, "a" and "b", were selected to assess the possible presence of antiparallel domains. Then, LEED *I-V* spectra (the intensity variations of diffraction spots as a function of the electron energy) for these spots were measured (red curves), as shown in (b). To determine the crystallographic origin of each diffraction spot, LEED *I-V* curves for the 10 and 01 spots of a monolayer MoS_2 single crystal, based on the atomic structure illustrated schematically, were computed using the SATLEED package developed by Barbieri and Van Hove.[13–15] As clearly seen in (b), the experimental LEED *I-V* curves for spots "a" and "b" closely match the simulated curves for the 10 and 01 spots, respectively. This agreement confirms that the monolayer MoS_2 is a single crystal and contains no antiparallel domains. To assess the single crystallinity of MoS_2 across the wafer, LEED patterns at 190 eV with a threefold symmetry were measured at eight locations across the sample. All locations exhibited identical threefold symmetric diffraction patterns, even on samples with an opposite miscut angle of c/m = 0.2°. Moreover, monolayer MoS_2 grown on a sapphire substrate with a different miscut angle of c/a = +0.15° displayed LEED *I-V* curves that remained consistent with the others. Furthermore, since the edge structures in the ±a directions are equivalent, the +a direction was also prepared. The LEED pattern for the substrate with c/a = 0.15° was consistent with those obtained for c/±m = 0.2°. Collectively, these results provide compelling evidence that the orientation of monolayer MoS_2 film is uniformly aligned across the wafer and that the epitaxial alignment during MOCVD growth is dictated not by step-edge directionality but by the interaction between MoS_2 and the underlying sapphire surface structure (or symmetry).

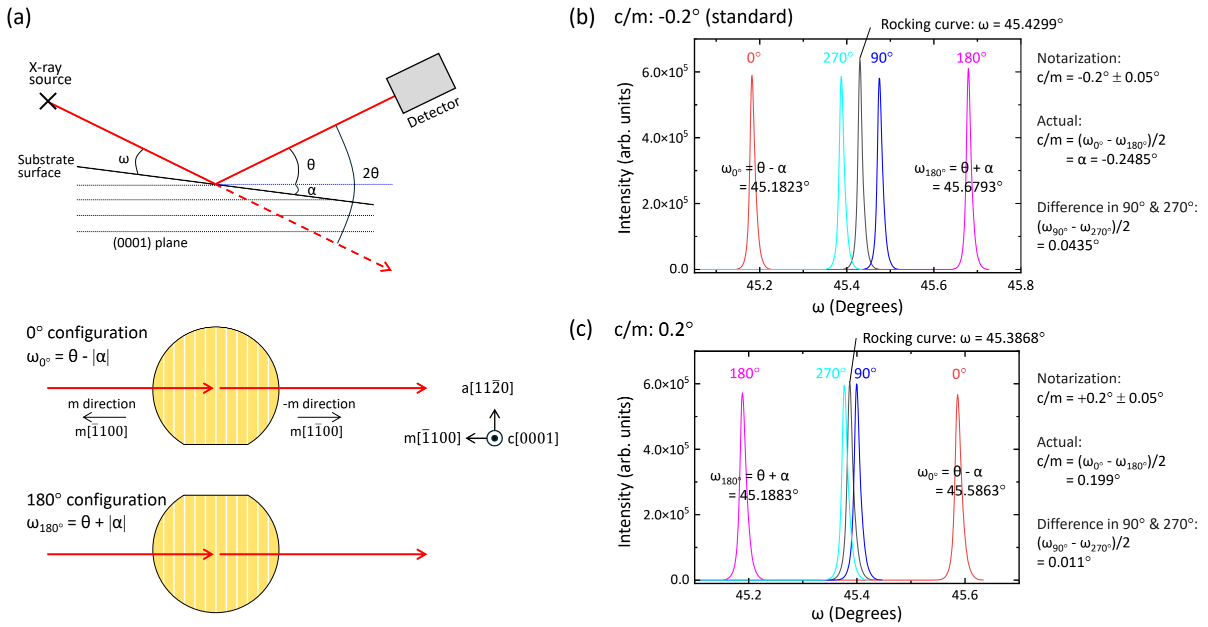

Supplementary Figure 16 (a) Out-of-plane XRD measurement setup for determining the miscut angle toward −m direction (c/m = −|α|°) of standard sapphire substrate. Wafer setting configurations for 0° and 180° setups. XRD rocking curve measurements of (b) c/m = −0.2° and (c) c/m = +0.2°.

Supplementary Note: The miscut angle of the sapphire substrate was evaluated by rocking curve measurements[16] using Rigaku SmartLab diffractometer equipped with a Ge (220) two-bounce channel-cut monochromator and HyPix-3000 detector. Initially, the sapphire substrate, oriented with a miscut angle of c/m = −0.2° (standard), was mounted on sample stage with the 0° configuration as depicted in (a), while the detector was positioned at the 2θ angle of 90.8° to match with the 00012 diffraction angle. Subsequently, the ω axis underwent rotation, leading to the peak intensity at $\omega_{0°} = \theta - |\alpha| = 45.1823°$, as illustrated in (b). Following this, the sapphire substrate was 180° rotated in plane (referred to as the 180° configuration), and the peak was found at $\omega_{180°} = \theta + |\alpha| = 45.6793°$ in the same way. Consequently, the miscut angle −|α| was determined to be $(\omega_{0°} - \omega_{180°})/2 = -0.2485°$, a value within the expected range considering the specified tolerance of −0.2° ± 0.05°. Measurements were also conducted at the 90° and 270° configurations. The incidental miscut angle along the a-axis was found to be negligible, assessed at 0.0435°. Furthermore, the miscut angle +|α| for the sapphire substrate with c/m = +0.2° was similarly evaluated to be +0.199°, as depicted in (c).

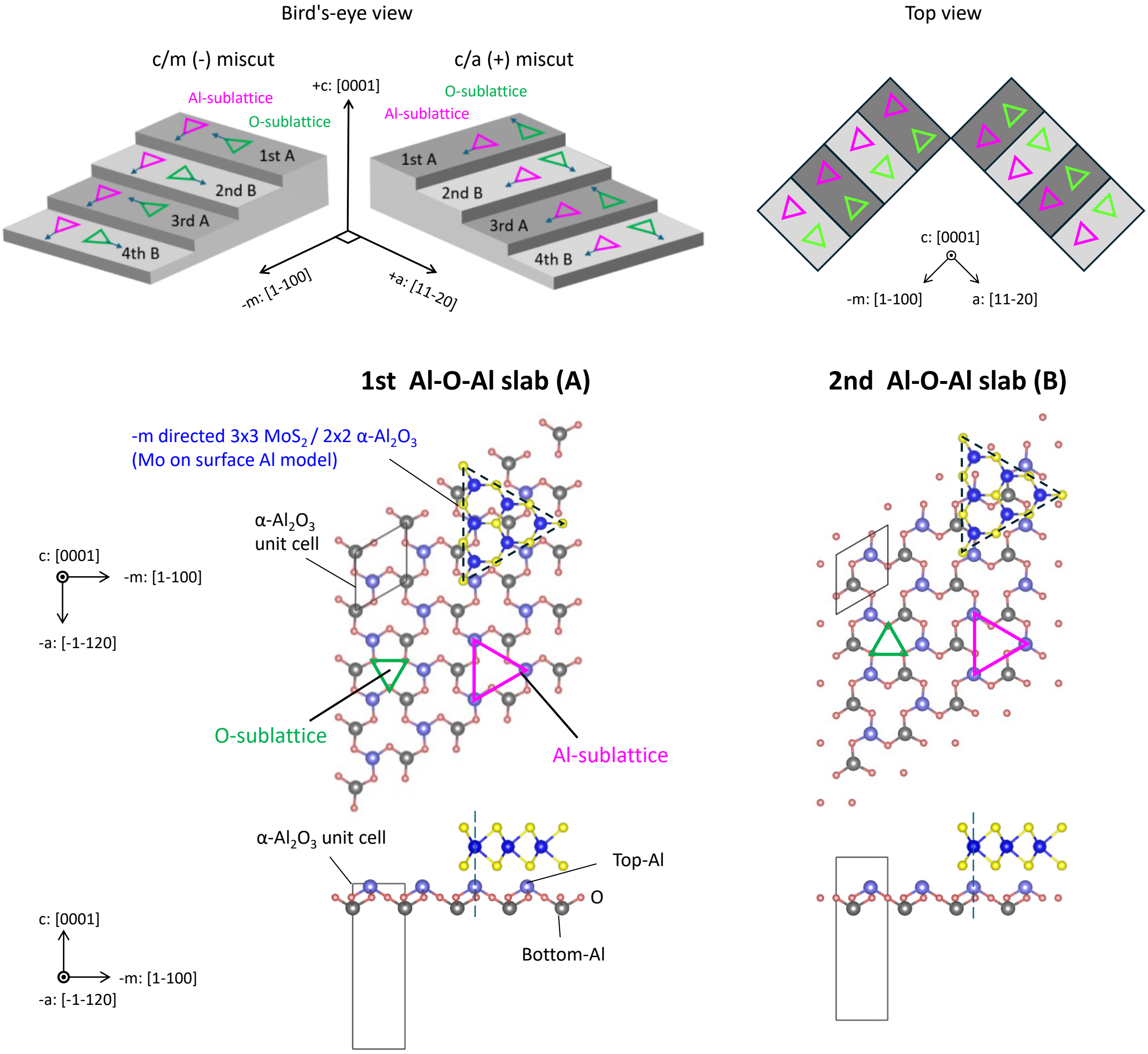

Supplementary Figure 17 Schematic illustration showing the difference between O-sublattice and Al-sublattice. In the previous report,[9] single crystalline monolayer MoS_2 with 30° orientation was obtained by preparing the AA slabs in miscut wafer of c/a = 1° in order to avoid 30°/90° antiparallel domains. In this case, the O-sublattice was taken into consideration, and, as is evident in above figures, alternating orientations of 30° and 90° appear at c/a = 0.2°. By increasing c/a to 1°, nucleation was restricted exclusively to the 30° orientation on AA slabs. In contrast, when considering the Al-sublattice, the 0° orientation consistently emerges in both the first layer (A slab) and the second layer (B slab). Accordingly, in the present experiment, MoS_2 triangle is directed to -m direction, indicating the growth proceeds in accordance with the symmetry of the Al sublattice. Furthermore, recent HAADF-STEM analysis[17] have elucidated that the atomic configuration adopts a structure in which Mo atoms are positioned directly above Al atoms.

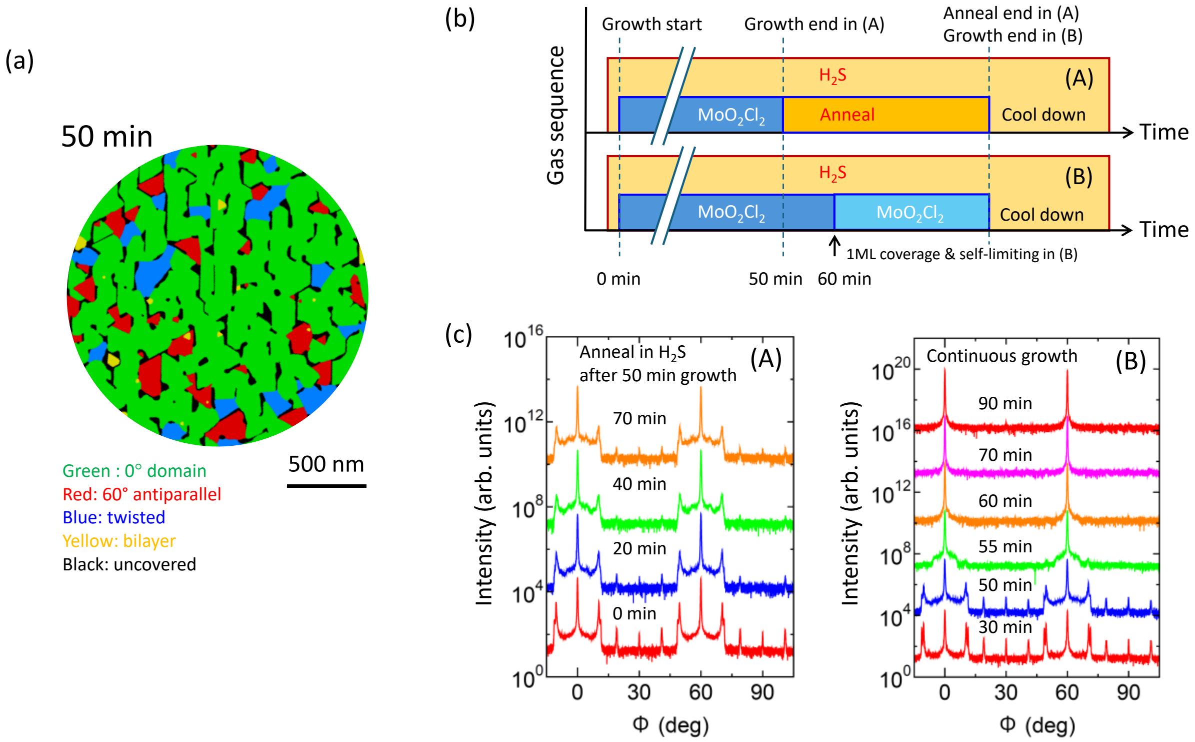

Supplementary Figure 18 (a) False-color map obtained by time-resolved differential DF-TEM analysis of MoS_2 acquired at 50 min at 975 °C. (b) Schematic illustration showing the gas flow sequence in two different growth experiments (A) and (B). (c) in-plane XRD intensities for the ϕ scan of MoS_2 acquired at different growth times at 975 °C for two different growth experiments (A) and (B).

Supplementary Note: To understand the GB migration mechanism, we conducted an additional experiment in which the growth was interrupted at 50 min by terminating the MoO_2Cl_2 flow while the H_2S annealing was continued. As shown in the in-plane XRD data (A) in (c), even when the H_2S annealing time was extended up to 70 min after the 50 min growth, further coalescence and annihilation of small-angle twisted domains into 0° domain was not detected by in-plane XRD. In contrast, when the growth continued without terminating the MoO_2Cl_2 flow until the end of growth time (up to 90 min), as shown in the in-plane XRD data (B) in (c), the self-aligned growth was clearly observed after 60 min. These results indicate that partial contact between domains alone is insufficient to drive GB migration by curvature-driven grain growth mechanism; continuous BG migration occurs only after the precursor gases are continuously supplied and low-angle twisted domains become completely enclosed by neighboring 0° domain.

Here, a key characteristic of GB migration during the above-mentioned self-aligned single-crystallization process is that the GB moves not only in the normal direction but also along its tangential direction. Recently, the importance of GB migration in grain growth and recrystallization processes has also been recognized in two-dimensional materials, and both experimental and simulation studies have been actively conducted.[18–21] It has been pointed out

that a simple curvature-driven mechanism, *i.e.*, mass transport solely based on grain curvature—is insufficient to explain many observed phenomena. Moreover, GB migration has been reported to involve coupled normal motion (perpendicular to the boundary) and tangential motion (parallel to the boundary), described as a shear-coupled mechanism. Nevertheless, GB migration is an extremely complex phenomenon; its dynamics and atomistic details are still far from being fully understood, and substantial further research is required.

(a)

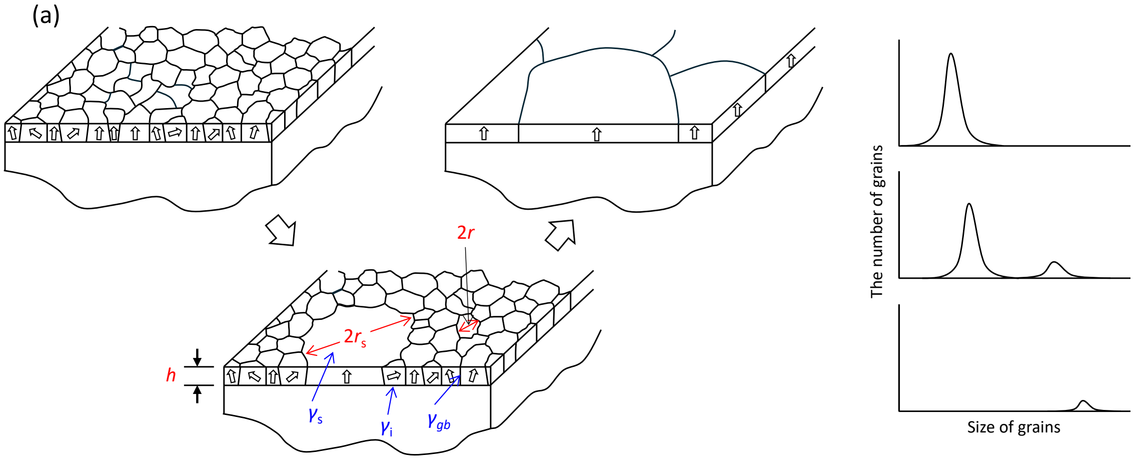

(b)

	Growth rate	Interface energy	Surface energy	Grain boundary
3D thin films	$$\frac{dR}{dt} = M\left\{\frac{\bar{\gamma}_i - \gamma_i}{h} + \frac{\bar{\gamma}_s - \gamma_s}{h} + \gamma_{gb}\left(\frac{1}{\bar{r}} - \frac{1}{r}\right)\right\}$$			
MoS_2 (present case)		Large (h = 0.65 nm)	~0	Small but not negligible

Supplementary Figure 19 (a) Schematic representations of epitaxial grain growth (EGG) in polycrystalline thin films on single crystalline substrates for conventional metallic systems,[22,23] Secondary grain growth is observed in the absence of nucleation of new grains. (b) The rate of grain growth is presented, where M denotes the average grain boundary mobility, γ_{gb} represents the average grain boundary energy, $\bar{r}$ is the average grain radius, r is the radius of the grain under consideration, r_s is the radius of the secondary grain, h is the film thickness, which is smaller than grain size. Additionally, γ_s is the surface energy of the growing grain, γ_i is the energy at the grain/substrate interface, $\bar{\gamma}_i$ and $\bar{\gamma}_s$ are the average values for the entire film.

Supplementary Note: For the metallic 3D thin film system, both $\bar{\gamma}_i$ and $\bar{\gamma}_s$ exhibit a strong dependence on the crystallographic orientation of individual grain. Grains with orientations that minimize the total energy (including both surface and interface contributions) preferentially grow more rapidly than grains with other orientations, provided that the initial grain sizes are approximately equivalent. Consequently, grain growth driven by surface and interface energies in films on single crystal substrates results in three-dimensionally constrained, epitaxial orientations, i.e., epitaxial grain growth is anticipated.

In contrast, additional intrinsic characteristics must be considered for 2D materials such as MoS_2. Owing to the absence of dangling bonds, the surface energy of MoS_2 is effectively negligible. Furthermore, since the film thickness, h, is limited to merely 0.65 nm, it is reasonable to assume that the interfacial energy term becomes crucial as the main driving force for single crystal formation in high-temperature MOCVD. This idea explains why the low angle rotated domains converge to the 0° domain. However, as predicted by DFT calculations (Figure

3e and Supplementary Figure 20b), there may not be a significant difference in the interfacial energy between the 0° and 60° domains on sapphire. The disappearance of the antiparallel domains seems to require consideration of another factor, namely the GB energy in the third term on the right side of the above equation. It is reported that the formation of GBs is accompanied by localized strain accumulation near the boundaries in monolayer MoS_2.[24–26] This strain serves as the driving force contributing to grain boundary annihilation for minimizing the total energy of system.

In fact, as seen in the 45 min sample shown in Figure 2c, the remaining low angle and antiparallel domains are already surrounded by the 0° domains and their size are small. Therefore, even if the interfacial energies of the 0° and 60° domains are almost the same, it is quite possible that curvature-driven GB migration occurs due to energy gain, by reducing the GB length and eventually disappearing. Thus, although the dominant driving force for single-crystal formation would be the interface energy term, the GB energy term may also play an important role, particularly in the annihilation of antiparallel and low-angle domains that persist just prior to achieving complete monolayer coverage.

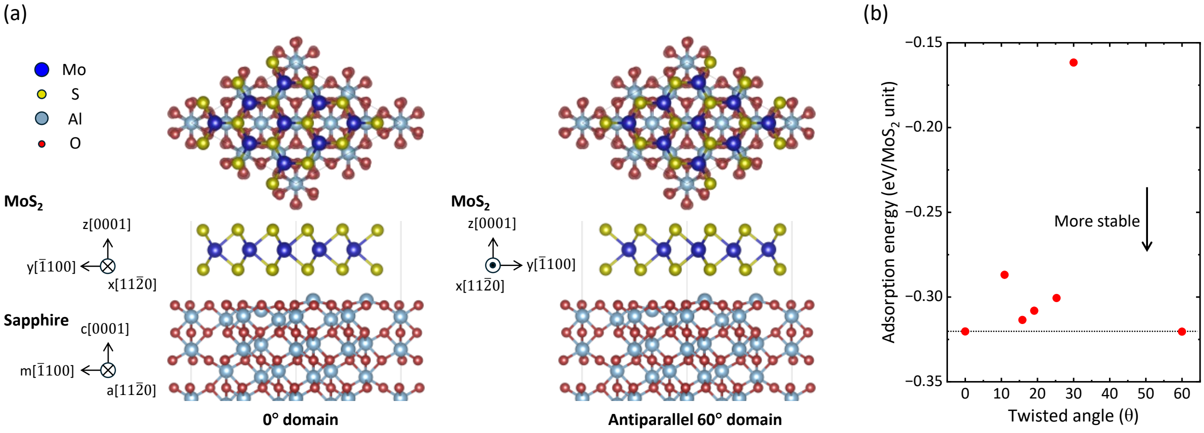

Supplementary Figure 20 (a) Schematic illustration of the 0° and antiparallel 60° domain monolayer MoS_2 on the sapphire substrate. Sapphire substrate with semiconductor property was structurally stabilized with monolayer MoS_2, where the rearrangement of top Al atoms can be seen. Note that the orientation of the 0° domain monolayer MoS_2 on the sapphire substrate is defined by the bottom layer of the unit cell, as specified in the standard crystallographic information (cif) file. See figure captions of Figure 3c. (b) DFT calculation of the adsorption energy as a function of different twisted angles between MoS_2 and sapphire substrate. In this calculation, to neglect the ambiguity in the atomic configuration of MoS_2 domain edge, it is assumed that commensurate infinite monolayer MoS_2 slabs adsorbed with different twisted angles on c-plane sapphire of stoichiometric single-Al-terminated surface, which has been reported most energetically stable.[27,28]

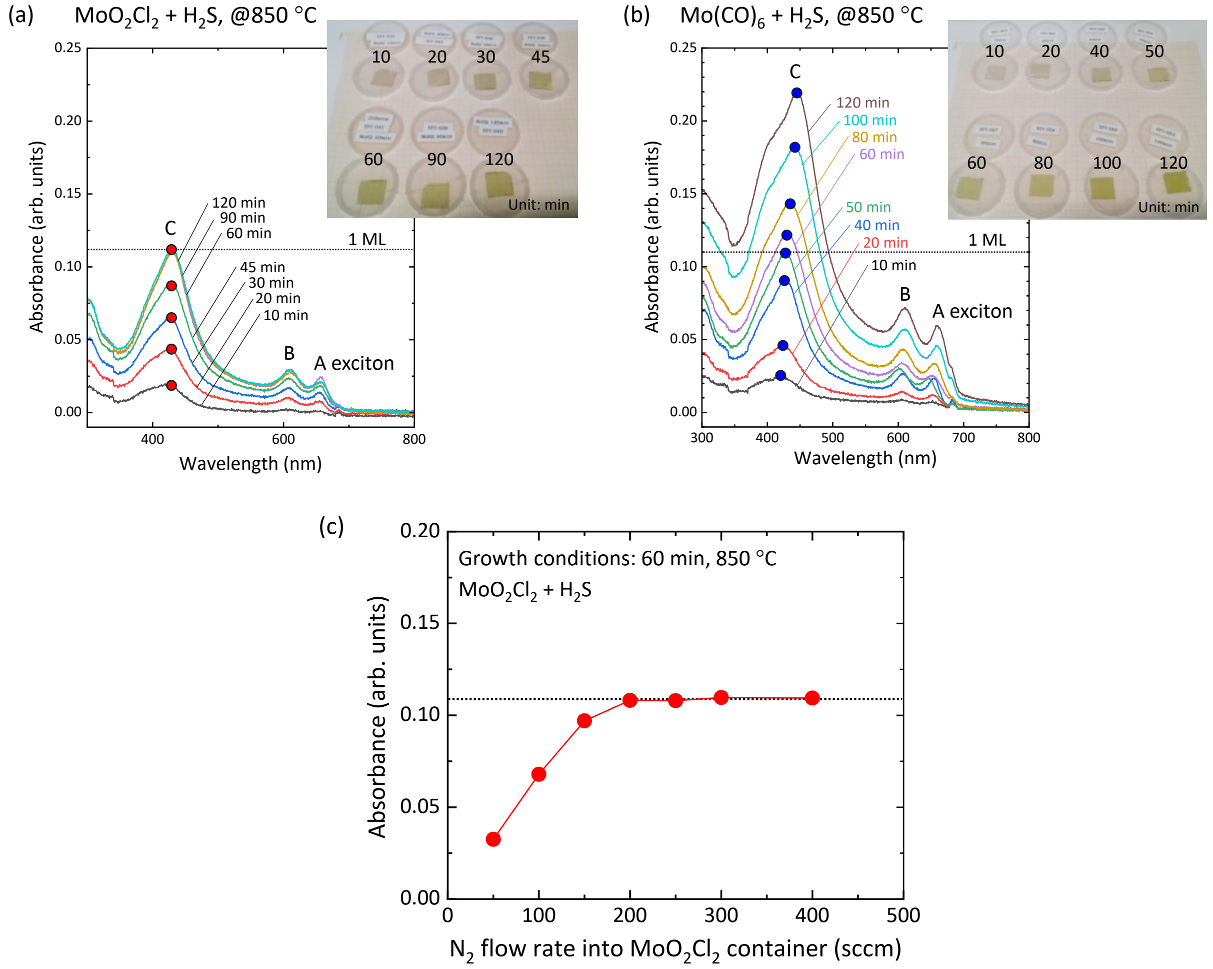

Supplementary Figure 21 Absorbance spectra of MoS_2 films grown on sapphire substrates for various growth times using (a) MoO_2Cl_2 and H_2S precursors and (b) $Mo(CO)_6$ and H_2S precursors. The characteristic A, B, and C exciton peaks are observed. Notably, double-side-polished sapphire substrates were employed for these absorbance measurements. The insets display photographs of MoS_2 films on sapphire substrates at different growth times. For the $Mo(CO)_6$ precursor, the darkness of the sapphire substrates increases with extended growth time, while for the MoO_2Cl_2 precursor, it saturates at 60 min, demonstrating the self-limited layer-controlled synthesis. (c) To investigate the effect of Mo precursor supply on the self-limiting behavior, the N_2 flow rate into the MoO_2Cl_2 container was increased to enhance Mo precursor delivery, and MOCVD growth was performed under conditions of 60 min and 850 °C. The absorbance at the C-exciton peak was plotted as a function of N_2 flow rate into the MoO_2Cl_2 container. This apparent saturated tendency with an increased Mo precursor supply provides compelling evidence for self-limiting behavior. This clearly indicates that the growth saturation is not limited by a reduction in growth rate originating from the slower kinetics of MoO_2Cl_2 precursor.

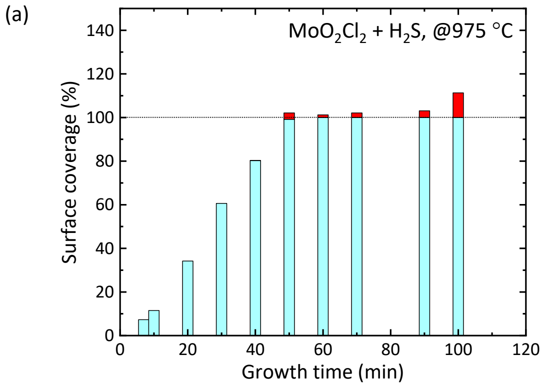

Supplementary Figure 22 Surface coverage of monolayer MoS_2 grown at 975 °C on sapphire substrates using MoO_2Cl_2 and H_2S precursor gases, as was determined from SEM. This data suggests that profound self-limiting growth behavior at an elevated temperature of 975 °C.

Supplementary Note: The observed self-limiting growth mechanism is ascribed to the site-selective adsorption behavior of the MoO_2Cl_2 precursor. The proposed scenario is as follows: MoO_2Cl_2 exhibits a strong preferential adsorption onto the dangling bonds of the sapphire substrate surface, while displaying markedly limited interaction with the chemically inert surface of monolayer MoS_2. This selective adsorption behavior likely inhibits further nucleation of a second MoS_2 layer by the reaction with H_2S. In contrast, the $Mo(CO)_6$ precursor appears to nucleate the second layer regardless of the underlying surface inertness of MoS_2. This distinction is also reflected in the time-resolved in-plane XRD spectra. As shown in Figure 2b and Supplementary Figure 6, even after reaching complete monolayer coverage at 60 min, the primary diffraction peak at 0° exhibits no additional broadening, and the integrated intensity saturates. In contrast, in the case of $Mo(CO)_6$ precursor, the 0° peak displays a pronounced broadening at extended growth durations of 90 and 120 min, as detected in Supplementary Figure 11. This phenomenon is ascribed to the mosaic spread introduced by a secondary epitaxial MoS_2 layer, which exhibits rather broad in-plane misalignment around 0°.

However, we hypothesize that this site selectivity of MoO_2Cl_2 may be disturbed by the presence of GBs. Unsaturated dangling bonds appear near GBs, and MoO_2Cl_2 may adsorb there and nucleate the second layer of MoS_2. This mechanism likely accounts for the subtle broadening observed in the XRD peak beyond 60 min of growth, as well as the slight appearance of 30° twisted domains in the spectrum acquired at 950 °C, as shown in Supplementary Figure 10. In fact, AFM measurements revealed a higher density of second-layer islands in samples grown at 950 °C relative to those synthesized at 975 °C. To fully leverage the self-limiting characteristics for achieving uniform monolayer coverage, it is therefore imperative to facilitate the in-plane self-alignment of grains and prevent the formation of GBs, thereby preserving the intrinsic site-selectivity of the precursor–substrate interaction.

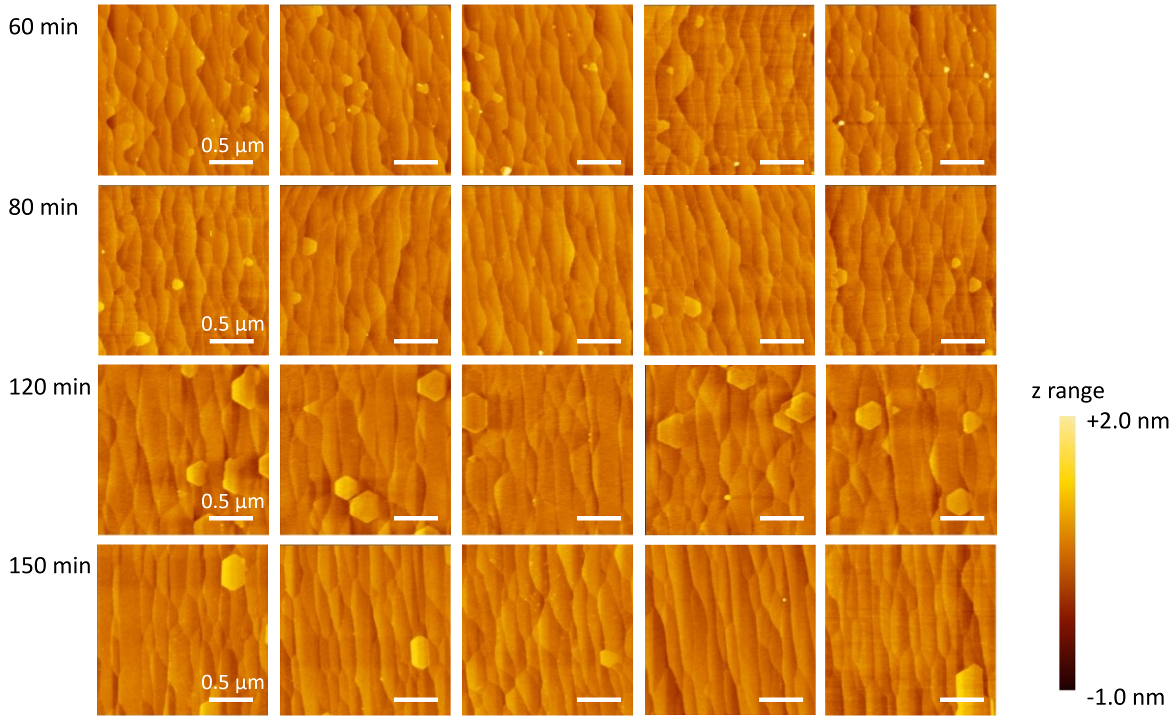

Supplementary Figure 23 AFM images of MoS_2 grown at 975 °C on sapphire substrates using MoO_2Cl_2 and H_2S precursor gases. The area number density of the second-layer domains is sufficiently suppressed, remaining limited to only a few domains per μm^2 even at the growth duration of 150 min. Although some second-layer islands are noticeable in the AFM image after 120 min of growth, the coverage of the second layer can be estimated to be less than 5%. As shown in Figure 4a, since the absorbance of a monolayer is 0.11, an additional 5% surface coverage of second layer would increase the absorbance only to 0.116, consistent with the observation that it does not affect the absorbance spectra. Since the island height is approximately 0.6 nm, the second layer has formed, and islands higher than three layers have not been observed.

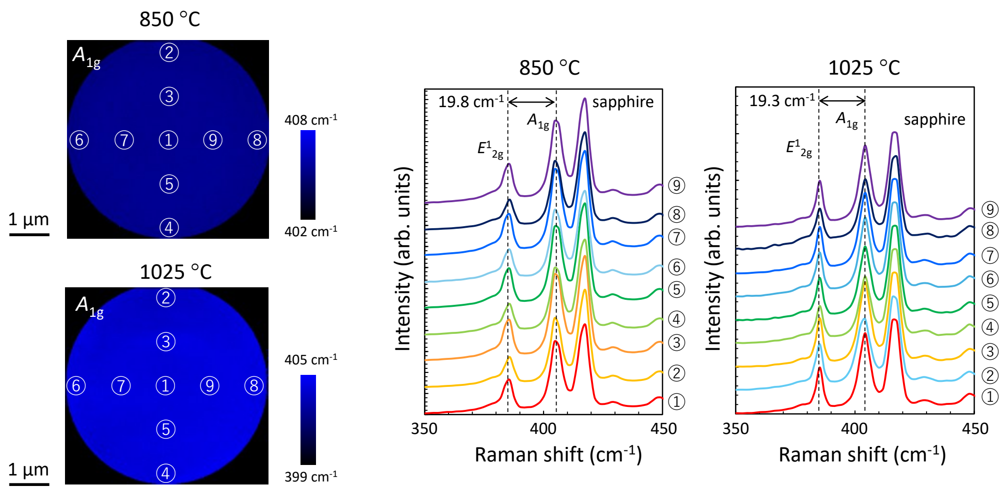

Supplementary Figure 24 Raman spectra for monolayer MoS_2 grown at 850 °C and 1025 °C for 60 min. Across the 2-inch wafer, the Raman mapping consists of 111,547 data points, and for each analysis region (①–⑨), the reported values represent the average of 1,116 points, corresponding to 1% of the total dataset. It is clear that difference between E^1_{2g} and A_{1g} is 19.8 cm^{-1} for 850 °C and 19.3 cm^{-1} for 1025 °C, which indicates monolayer MoS_2.

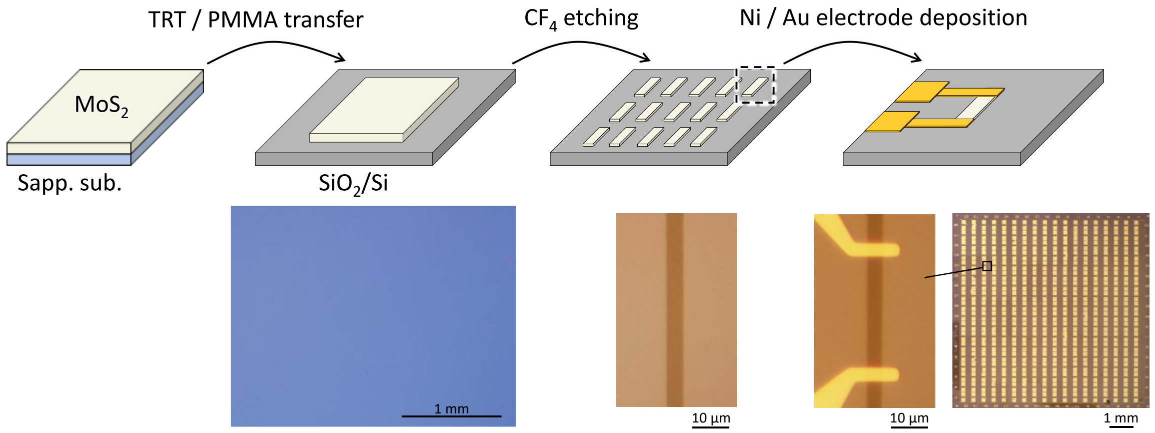

Supplementary Figure 25 Schematic illustration of device fabrication process. First of all, monolayer MoS_2 grown on a sapphire substrate was transferred to a SiO_2/Si substrate.[29] The MoS_2 channel region was defined by CF_4 etching. Finally, Ni/Au electrode was formed for back gate MoS_2 FET.

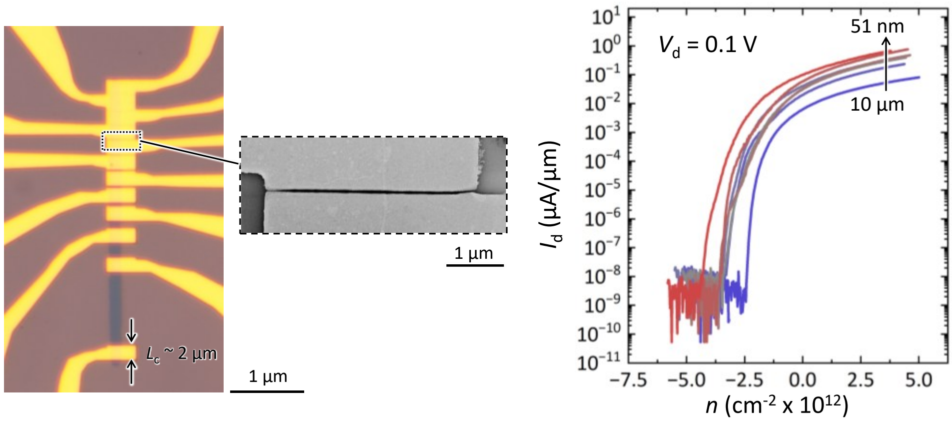

Supplementary Figure 26 Photograph of short-channel devices with channel lengths ranging from 2 µm to 51 nm, fabricated using electron beam lithography. The monolayer MoS_2 was grown at 975 °C on the sapphire substrate and transferred on the SiO_2/Si substrate. As the channel length decreases, a clear enhancement in drain current is observed. Using the transfer length method, the contact resistance was estimated to be approximately 150 kΩµm. Consequently, the mobilities of the short-channel devices, while measured to be on the order of a few cm^2/V·s, primarily reflect the influence of contact resistance rather than limitations in channel quality.

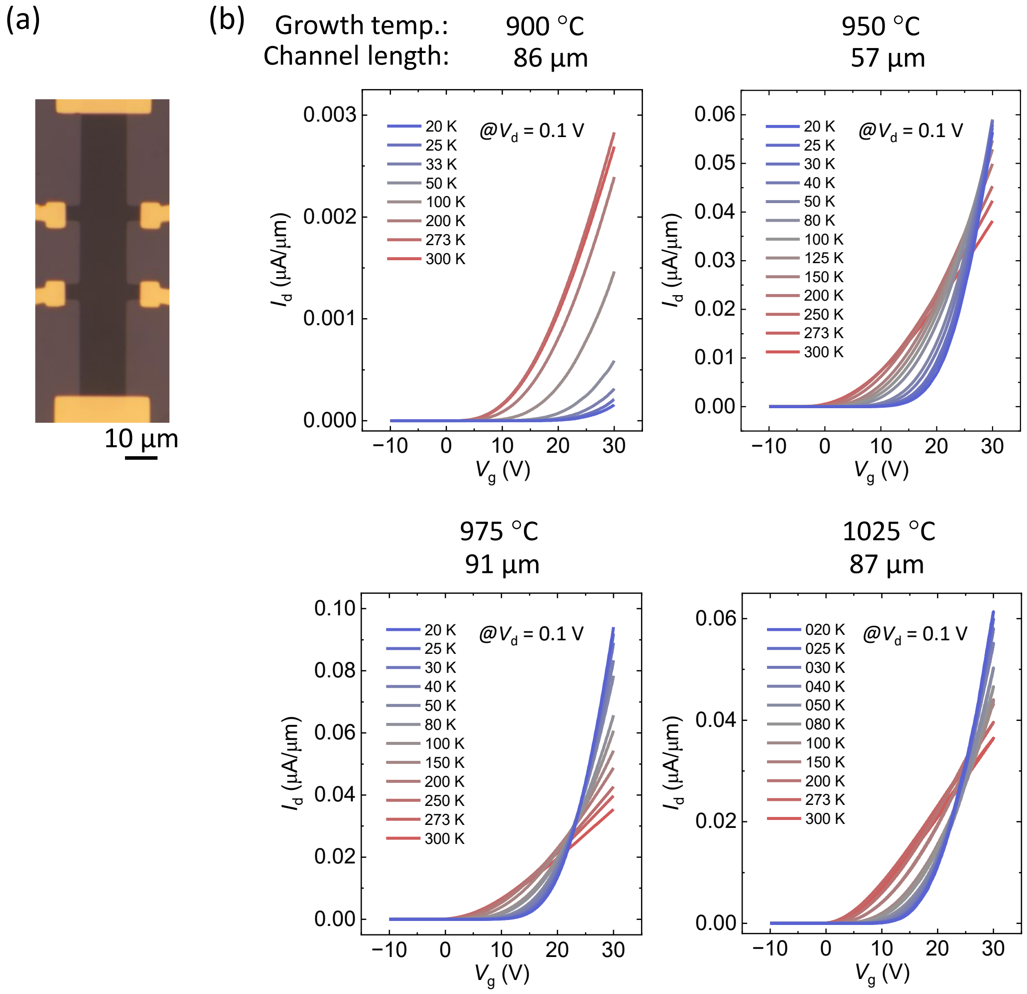

Supplementary Figure 27 (a) Photograph of typical four probe device. (b) Two probe I_d-V_g curves at various temperatures for monolayer MoS_2 transferred onto SiO_2/Si substrate. The growth temperature and channel length are indicated at the top of figures.

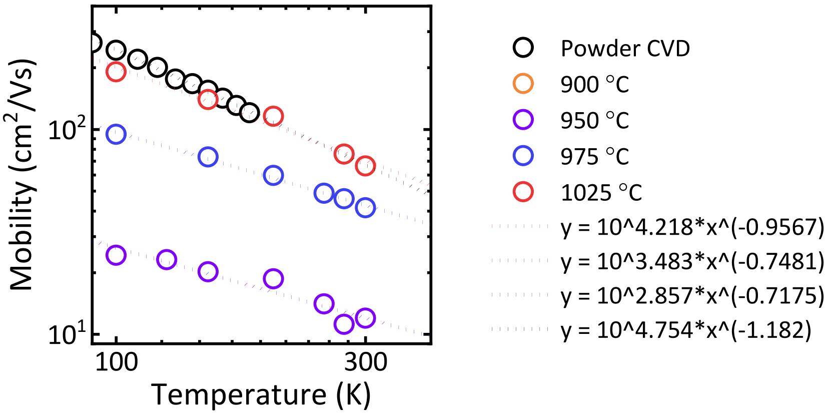

Supplementary Figure 28 The mobility of monolayer MoS_2 in vacuum was predicted to be limited by optical phonon in MoS_2 for the temperature around room temperature and temperature coefficient of $\gamma = 1.69$ in $\mu \sim T^{-\gamma}$.[30] However, this is drastically changed when MoS_2 is placed on different substrates because surface optical phonon of substrate is involved in the carrier scattering in MoS_2 through the equation $\Gamma \propto \omega_{so} exp(-\hbar\omega_{so}/k_B T)$, where $\hbar\omega_{so} = 22$, 59, 100 meV for HfO_2, SiO_2, *h*-BN, respectively.[31] Because of surface optical phonon scattering, when HfO_2 is used as back gate oxide, the mobility is generally reduced. However, HfO_2 is often used as back gate oxide for mobility measurement of MoS_2 and high mobility is often obtained. In contrast, MoS_2 encapsulated in *h*-BN exhibit $\gamma = 1.9$,[32] which is higher than theoretical expectation. Therefore, at this moment, the limiting factor for temperature dependence of mobility for MoS_2 on substrates has not been clarified yet. The situation is further complicated by the role of dielectric screening from high-κ oxides. In our present case, MoS_2 is placed on SiO_2/Si substrate and γ increases with improving the crystallinity of MoS_2.

Supplementary References

1. Gäggeler, H. W. & Türler, A. *Gas-phase chemistry of superheavy elements*. (Springer, Berlin, Heidelberg, 2014).
2. Saeki, Y. & Matsuzaki, R. Thermochemical properties of molybdenum dioxydichloride. *Denki Kagaku oyobi Kogyo Butsuri Kagaku* **34**, 504–505 (1966).
3. Sato, T., Shibata, H., Hayashi, H., Takano, M. & Kurata, M. Chlorination of UO_2 and $(U,Zr)O_2$ solid solution using $MoCl_5$. *J Nucl Sci Technol* **52**, 1253–1258 (2015).
4. Ohta, T., Cicoira, F., Doppelt, P., Beitone, L. & Hoffmann, P. Static vapor pressure measurement of low volatility precursors for molecular vapor deposition below ambient temperature. *Chemical Vapor Deposition* **7**, 33–37 (2001).
5. Chubarov, M., Choudhury, T. H., Zhang, X. & Redwing, J. M. In-plane x-ray diffraction for characterization of monolayer and few-layer transition metal dichalcogenide films. *Nanotechnology* **29**, 055706 (2018).
6. Dumcenco, D. *et al.* Large-area epitaxial monolayer MoS_2. *ACS Nano* **9**, 4611–4620 (2015).
7. Chubarov, M. *et al.* Wafer-scale epitaxial growth of unidirectional WS_2 Monolayers on sapphire. *ACS Nano* **15**, 2532–2541 (2021).
8. Dosenovic, D. *et al.* Mapping domain junctions using 4D-STEM: toward controlled properties of epitaxially grown transition metal dichalcogenide monolayers. *2D Mater* **10**, 045024 (2023).
9. Fu, J. H. *et al.* Oriented lateral growth of two-dimensional materials on c-plane sapphire. *Nat Nanotechnol* **18**, 1289–1294 (2023).
10. Deb, P. *et al.* Imaging polarity in two dimensional materials by breaking Friedel's law. *Ultramicroscopy* **215**, 113019 (2020).
11. Psilodimitrakopoulos, S. *et al.* Optical versus electron diffraction imaging of Twist-angle in 2D transition metal dichalcogenide bilayers. *npj 2D Mater Appl* **5**, 77 (2021).
12. Yin, X. *et al.* Edge nonlinear optics on a MoS_2 atomic monolayer. *Science* **344**, 488–490 (2014).
13. Ohtake, A., Suga, T., Goto, S., Nakagawa, D. & Nakamura, J. Atomic structure of the Se-passivated GaAs(001) surface revisited. *Sci Rep* **13**, 18140 (2023).
14. Rous, P. J. & Pendry, J. B. The theory of tensor LEED. *Surf Sci* **219**, 355–372 (1989).
15. Rous, P. J. *et al.* Tensor LEED: A technique for high-speed surface-structure determination. *Phys Rev Lett* **57**, 2951–2954 (1986).
16. Doucette, L. D., da Cunha, M. P. & Lad, R. J. Precise orientation of single crystals by a simple x-ray diffraction rocking curve method. *Rev Sci Instrum* **76**, 036106 (2005).
17. Kano, E., Nara, J., Takenaka, K., Yasuno, T., Atsumi, K., Li, S., Nishimura, T., Kanahashi, K., Uzuhashi, J., Nagashio, K., Sakuma, Y., Ikarashi, N. Direct observation of atomic configuration of a highly oriented MoS_2 film/ α -$Al_2O_3(0001)$ using atomic resolution electron microscopy, *Appl. Phys. Lett.*, **127**, 252102 (2025).
18. Ren, X. & Jin, C. Grain boundary motion in two-dimensional hexagonal boron nitride. *ACS Nano* **14**, 13512–13523 (2020).

19. Waters, B. & Huang, Z.-F. Grain rotation and coupled grain boundary motion in two-dimensional binary hexagonal materials. *Acta Mater* **225**, 117583 (2022).
20. Taha, D., Mkhonta, S. K., Elder, K. R. & Huang, Z.-F. Grain boundary structures and collective dynamics of inversion domains in binary two-dimensional materials. *Phys Rev Lett* **118**, 255501 (2017).
21. Azizi, A. *et al.* Dislocation motion and grain boundary migration in two-dimensional tungsten disulphide. *Nat Commun* **5**, 4867 (2014).
22. Thompson, C. V. Grain growth in thin fims. *Annu. Rev. Mater. Sci.* **20**, 245–268 (1990).
23. Thompson, C. V., Floro, J. & Smith, H. I. Epitaxial grain growth in thin metal films. *J Appl Phys* **67**, 4099–4104 (1990).
24. Nalin Mehta, A. *et al.* Grain-boundary-induced strain and distortion in epitaxial Bilayer MoS_2 lattice. *J Phys Chem C* **124**, 6472–6478 (2020).
25. Ly, T. H. *et al.* Misorientation-angle-dependent electrical transport across molybdenum disulfide grain boundaries. *Nat Commun* **7**, 10426 (2016).
26. Hong, J. *et al.* Atomistic dynamics of sulfur-deficient high-symmetry grain boundaries in molybdenum disulfide. *Nanoscale* **9**, 10312–10320 (2017).
27. Godin, T. J. & LaFemina, J. P. Atomic and electronic structure of the corundum (α-alumina) (0001) surface. *Phys Rev B* **49**, 7691–7696 (1994).
28. Wang, X.-G., Chaka, A. & Scheffler, M. Effect of the environment on α-A_2O_3 (0001) surface structures. *Phys Rev Lett* 84, 3650–3653 (2000).
29. Kim, H., Ovchinnikov, D., Deiana, D., Unuchek, D. & Kis, A. Suppressing nucleation in metal-organic chemical vapor deposition of MoS_2 monolayers by alkali metal halides. *Nano Lett* **17**, 5056–5063 (2017).
30. Kaasbjerg, K., Thygesen, K. S. & Jacobsen, K. W. Phonon-limited mobility in n-type single-layer MoS_2 from first principles. *Phys Rev B* **85**, 1–16 (2012).
31. Ma, N. & Jena, D. Charge scattering and mobility in atomically thin semiconductors. *Phys Rev X* **4**, 011043 (2014).
32. Cui, X. *et al.* Multi-terminal transport measurements of MoS_2 using a van der Waals heterostructure device platform. *Nat Nanotechnol* **10**, 534–540 (2015).
