# Supplementary material for: Self-aligned and self-limiting van der Waals epitaxy of monolayer MoS2 for scalable 2D electronics
Source: Nat Commun. 2026 Jan 21;17:602. doi: 10.1038/s41467-026-68320-8 (PMC12824160; doi:10.1038/s41467-026-68320-8)
Supplement: Supplementary file 2 — Transparent Peer Review file [file 41467_2026_68320_MOESM2_ESM.pdf]

nature portfolio

Peer Review File

Self-aligned and self-limiting van der Waals epitaxy of monolayer MoS_2 for scalable 2D electronics

Corresponding Author: Professor Kosuke Nagashio

Parts of this Peer Review File have been redacted as indicated to remove third-party material.

This file contains all reviewer reports in order by version, followed by all author rebuttals in order by version.

Attachments originally included by the reviewers as part of their assessment can be found at the end of this file.

Version 0:

Reviewer comments:

Reviewer #1

(Remarks to the Author)
See attachment.

[Editorial Note: see end of file]

Reviewer #2

(Remarks to the Author)
Comments on the Manuscript: “Self-aligned and self-limiting van der Waals epitaxy of monolayer MoS_2 to annihilate grain boundaries for scalable 2D electronics”
The manuscript reports a compelling growth concept wherein misoriented monolayer MoS_2 domains undergo a self-aligned coalescence process to yield single-orientation films on c-plane sapphire via MOCVD. The authors provide experimental support using in-plane XRD, differential dark-field TEM, 4D-STEM, and SHG mapping, and further demonstrate promising field-effect mobility. While the concept of grain boundary (GB) annihilation through epitaxial grain coalescence is interesting and potentially impactful, several aspects of the mechanistic interpretation, statistical characterization, and device-level validation remain insufficiently addressed.
I believe this work has merit but, in its current form, I do not recommend publishing this work in Nature Communications.
Below, I outline my major concerns:
-Mechanism Uncertainty
1. The manuscript would benefit from a more detailed description of the MOCVD process, particularly regarding the timing of precursor delivery and shutdown, as well as the cooling protocol. It appears that all in-plane XRD and structural characterizations were performed post-growth, meaning that the samples had undergone a full cooling stage prior to measurement.
This raises an important mechanistic question: why does the transition to single-orientation crystallinity appear to occur sharply around the 40-minute growth mark? While the authors attribute this transition to in-situ domain coalescence and grain boundary migration, an alternative possibility is that some degree of nucleation and growth occurs during the cooling stage. At that point, the system may lack sufficient thermal energy to drive proper orientation, leading to the formation of metastable, misoriented domains.
Furthermore, the observed suppression of new misoriented domains at later growth stages (e.g., at 40 min) may not reflect an intrinsic self-alignment mechanism, but instead result from reduced available surface area for new nucleation events.
2. Would the timing of this crystallinity transition shift if the supply rates of MoO_2Cl_2 and H_2S were varied? A parametric study would clarify the role of precursor flux in the coalescence mechanism.
3. The author believes that their unidirectional MoS2 with 0° alignment is due to the interface energy between MoS2 and sapphire. However, both their DFT calculations (Fig. S15) and prior literature (Refs. 7, 8, 13, 37) consistently suggest that the 30° configuration is thermodynamically more stable. Moreover, Figure 2a does not clearly reveal a distinct “buffer layer” that could alter this behavior. The authors should reconcile these inconsistencies and provide a more convincing

explanation.
4. Although the manuscript claims orientation is not governed by step edges, the sapphire substrates were intentionally annealed to create step-terrace structures. The authors should clarify this contradiction. In the meantime, the author should try other sapphire planes (e.g., A-plane, M-plane) or miscut direction (e.g. C to A plane) to confirm the MoS_2 lattice is not governed by step edges.
5. The AFM images presented in Figures S2c, 1b, and 3b reveal that the sapphire step structure evolves significantly with growth time and temperature. Such step morphology changes—particularly under high-temperature conditions exceeding 950 °C—could plausibly influence domain orientation selection, nucleation behavior, and coalescence dynamics.
I encourage the authors to discuss the potential impact of these evolving step features on the orientation control mechanism proposed in the manuscript. In particular, the work by Zheng et al. (Nature Communications 14, 592 (2023)) provides relevant insights into step-edge reconstruction on sapphire at elevated temperatures and its influence on epitaxial alignment of TMDCs. That study emphasizes the importance of growth timing and surface evolution in controlling domain orientation, which appears to share similarities with the present work.
-Single Crystallinity Assessment
6. While DF-TEM (Fig. 1d) reveals local orientation, the sampling area is too limited to convincingly support wafer-scale single-crystallinity. The authors should provide more statistically significant data, possibly via multi-area DF-TEM.
7. Could strain or tilt introduced during the transfer process affect DF-TEM interpretation? Additionally, are there high-resolution images available showing grain boundary structures between 0° and 60° domains? In the 35-minute sample (Fig. 1d), some flakes visually appear antiparallel, yet DF-TEM indicates they are all 0°.
8. The grain size is reported to be ~200 nm, while the SHG laser spot size typically exceeds 1 μm. The observed intensity fluctuations in Fig. 1f and Fig. S10 suggest the presence of multiple domains within each spot. Therefore, SHG mapping may not be sufficient to conclude a reduction in grain boundaries without supporting high-resolution data. To me, the provided mapping image contains many grain boundaries.
-Self-limiting monolayer
9. The claim of self-limited monolayer growth requires further scrutiny. In Figure 3b, the AFM images clearly show bilayer triangular MoS_2 domains, which contradicts the assertion of monolayer-only growth. Moreover, the Raman $A_{1}g$ peak variation of ~2 cm^{-1} in Figure 3e aligns with the known difference between monolayer (Δ = 19 cm^{-1}) and bilayer (Δ = 21 cm^{-1}) MoS_2, suggesting that non-negligible bilayer regions are present across the wafer.
While MoO_2Cl_2 provide slower kinetics compared to $Mo(CO)_6$, reduced growth rate alone does not confirm a self-limiting mechanism. To convincingly establish self-limitation, the authors should perform a systematic study by increasing the MoO_2Cl_2 supply or extending the growth time to demonstrate that no additional layer forms under higher precursor flux or prolonged exposure.
-Inhomogeneous device performance
10. In Figure 4, the authors present electrical characteristics from several batches of MoS_2 FETs. However, the data exhibit substantial device-to-device variation—exceeding three orders of magnitude in current density (Figure 4b) and showing large mobility spread (Figure 4e). This high degree of inhomogeneity raises concerns about the claim of wafer-scale single crystallinity.
General concerns
11. Different characterizations were performed on samples grown at different temperatures—e.g., thickness analysis at 850 °C, grain mapping at 975 °C, and devices at 1025 °C. The rationale for these choices should be explained. Is the epitaxial mechanism or material quality consistent across these temperatures?
12. The manuscript would benefit from additional language polishing and restructuring for clarity and flow.

Reviewer #3

(Remarks to the Author)
The growth of single-crystalline monolayer TMDC films is highly desired for their lab-to-fab transition. Focusing on this objective, the manuscript “Self-aligned and self-limiting van der Waals epitaxy of monolayer MoS2 to annihilate grain boundaries for scalable 2D electronics” reported an MOCVD growth approach for achieving large-area monolayer MoS2 single crystals, based on the self-alignment of initially-misoriented domains. The growth of MoS2 also occurs layer-by-layer, leading to good spatial uniformity and high electronic performance.

In my opinion, the growth mechanism revealed in this work is very interesting and is fundamentally different from previous efforts. It might be even more robust/reproducible, if the growth process can be fully understood and controlled, since there is less stringent requirement on the orientation of the initial MoS2 nuclei. With that being said, I think there are several important questions to be addressed to better elucidate the growth mechanism and understand why this particular growth process is governed by a totally different mechanism from previous ones:

1. Sapphire substrate requirements: By comparing the LEED patterns of MoS2 grown on c-sapphire with opposite miscut angles (Fig. 2c), the authors concluded that the alignment of MoS2 is governed by the terrace instead of the step edges. Does this mean that this growth approach is also compatible with conventional c-sapphire or different miscut directions? Further discussion or experimental evidence would be helpful. Additionally, the authors are suggested to perform AFM characterization on the MoS2 flakes at an initial growth stage to examine whether the nucleation mainly occurs at step edges or terraces.

2. Formation of isolated domains of twisted MoS2: In Fig. 1c and S4, in-plane XRD observes the preferential twist angles of isolated MoS2 domains (e.g., in the 20 min growth sample), and the angles match with MoS2/sapphire lattice model. However, the formation energy of these twisted domains should be significantly higher than that of 0-degree and 60-degree

domains (as shown in DFT calculation results in Fig. 2e and S15). Why do the twisted domains form in this particular growth approach? It would be necessary to discuss the mechanism and also compare it with existing work.

3. MoS2 domain alignment mechanism: Two possible mechanisms for domain alignments are compared, including the rotation model and grain boundary migration model. The authors identified that the grain boundary migration is a more plausible mechanism. However, why does the grain rotation not happen at the early nucleation/growth stage? DFT calculations are desired to compare the activation energies for domain rotation versus GB migration. Moreover, due to the similar free energies of 0-degree and 60-degree domains, I think the conversion from 60-degree to 0-degree domain requires not only a large activation energy, but also a precise control of growth thermodynamics, which should be very challenging. The authors are suggested to discuss further how this growth approach achieves good controllability.

4. Temperature-dependence of self-aligned coalescence: The authors identified that the domain alignment works for growth temperatures above 900 C. However, I think it is also possible that the growth rate and grain boundary migration rate are lower at low temperatures, so that additional growth time is needed for the full merge of MoS2 domains and for the self-alignment.

5. Mobility extraction of the MoS2 device: The devices used for measuring the electron mobility have quite long channels (tens of micrometers), which are not comparable to the practical application scenario of 2D nanoelectronics. For example, Ref. 48 uses a short-channel device (200 nm), which can lead to lower mobility values but is closer to the practical application. The authors are suggested to fabricate short-channel devices to better estimate and benchmark the mobility.

6. Defect density measurement: The authors discussed the MoS2 device performance and the crossover from thermally activated to phonon-limited transport in MoS2 grown at higher temperatures (Fig. 4d). It would be helpful to directly measure the defect density by using STM, conductive AFM, or STEM, etc., to better correlate with electrical data and compare with previous work.

7. Batch-to-batch reproducibility of the self-aligned coalescence phenomenon: On page 7 of the manuscript, the authors mentioned that the time-dependent domain orientation evolution is highly reproducible in MOCVD but inconsistent in powder-source CVD. It is needed to include the data from different growth batches to support this conclusion. For example, error bars for the inset of Fig. 1c are required.

Version 1:

Reviewer comments:

Reviewer #1

(Remarks to the Author)
In their response letter and revised manuscript, the authors have largely addressed the comments raised by me. However, for the claim “phonon limited electron mobility at room temperature”, I still do not agree. Temperature-dependent measurements can only tell us that which factors dominate the changes in mobility, while they cannot tell us what specifically dominates the mobility at a given temperature. The mobility is typically limited by defect and phonon scattering at room temperature. Since defect scattering is not particularly sensitive to temperature, so the change in mobility with temperature appears to be phonon-dominated. In addition, the full name of word “vdW” is already provided in line 20, so it is unnecessary to repeat it in line 22.

Reviewer #2

(Remarks to the Author)
Before providing my detailed comments on the revised manuscript, I must note that the format of the author’s response letter is difficult to read and follow. This has made the review process more time-consuming. I strongly recommend that the authors reply to each reviewer comment one by one, and clearly indicate the corresponding revisions (e.g., by quoting or pointing to the changed paragraph). In addition, please avoid asking the reviewer to cross-reference other reviewers’ comments before fully addressing the current question. It is unreasonable to expect reviewers to repeatedly switch back and forth between different comments.
In terms of content, I find the most interesting aspect of this work to be the proposal of a new mechanism for achieving single-crystal TMD films. However, in the response, the authors emphasized that “the main purpose of this manuscript is not to reveal the growth mechanisms.” This statement is confusing and somewhat contradictory, since without a convincing mechanism the generality of the method remains questionable. There still several key questions remain unclear:
1. Why is 0° considered the most stable orientation? Normally, lower adsorption energy corresponds to a more stable state. Please clarify your DFT calculation methodology and re-examine your results. (Suggested references: Small 2020, 16, 2000596; ACS Nano 2023, 17 (2), 1196–1205).
2. Why does the mechanism occur only after contact or specifically at 40 minutes? In principle, the timing of this crystallinity transition should vary if the supply rates of MoO_2Cl_2 and H_2S are changed, since precursor supply affects the timing of contact. Additional experimental evidence is needed to substantiate this claim.
3. How is this process related to the substrate? The authors suggest that higher growth temperatures (e.g., 1000 °C), which are believed to cause surface roughening, lead to degraded crystallinity. Could direct evidence for this roughening be provided, and could the authors further explain how surface roughness impacts crystallinity? In this context, it would be

highly valuable to demonstrate results on A-plane or M-plane sapphire. This would help clarify whether substrate orientation guides the growth or whether the observed behavior is truly a self-aligned phenomenon. More experimental support is required to strengthen this point.
4. Please avoid overclaiming. For example, the statement: “It is worth noting that the temperature dependence of mobility in MOCVD-grown MoS_2 films has, to the best of our knowledge, not yet been reported.” is inaccurate. First, there are already published studies reporting temperature-dependent electrical measurements of MOCVD-grown MoS_2. (Nature Electronics volume 7, pages356–364 (2024)) Second, even if this were not the case, such a statement does not add substantive novelty to this manuscript.

Reviewer #3

(Remarks to the Author)
I appreciate the author’s significant effort in responding to my questions and revising the manuscript. Upon reading the revised version, I am more convinced by the observed domain self-alignment phenomenon and the proposed grain boundary annihilation mechanism. The device results presented in the revised manuscript are also helpful to demonstrate the electrical performance of the MOCVD-synthesized film. Nevertheless, there are still several points from my previous comments that I feel the authors should address more adequately. I would like to clarify my questions further, and look forward to the authors’ response and revision:

1. The authors partially addressed my Comments 2-3 by suggesting that the probability of twisted domain formation is governed by Maxwell-Boltzmann distribution, and that the domain rotation is suppressed as domain size increases. However, in my original Comments 2-3, I was also concerned about why the twisted domain percentage in this work (prior to coalescence) is higher than existing reports, and why the self-alignment of 60-degree and 0-degree domains was unobserved in previous growths (e.g., ACS Nano, 2015, 9, 4611). To address these questions and to provide guidance for future efforts on the growth of single-crystalline TMD monolayers, I think it is necessary to provide more discussions on the growth mechanism and clearly compare with existing growth approaches.

2. In my previous Comment 4, my intent was to suggest the authors characterize the domain alignment in coalesced MoS2 grown at lower temperatures (e.g., 850 C). Indeed, the coalescence would take a longer time due to reduced growth rates, but it is still practical in my opinion. I believe this experiment would be meaningful because it could reveal whether the domain alignment is solely constrained by growth time or also by the temperature-dependent grain boundary migration rate. In case if a lower temperature, longer growth time also result in domain self-alignment, the authors should clarify it in the manuscript.

3. In the response to my Comment 4, samples grown at 850 C were non-uniform and polycrystalline, but why did the authors present a uniform A1g Raman mapping for the 850 C sample in Fig. 3e? This discrepancy needs to be clarified.

Version 2:

Reviewer comments:

Reviewer #1

(Remarks to the Author)
I cannot agree the claim “phonon limited electron mobility at room temperature”. In the absence of defect scattering or other impurity scattering mechanisms, where only phonon scattering is operative, the measured mobility of monolayer MoS2 should approach its intrinsic theoretical limit (according to the Matthiessen’s rule of mobility $1/\mu=1/\mu_{imp} +1/\mu_{ph}$). This should represent the highest mobility achievable experimentally in monolayer MoS2 devices and therefore will outperform all prior reports. Obviously, this is not the case as claimed by the authors. The as-achieved mobility of 66 cm2/Vs at room temperature is significantly worse than the previous state-of-the-art results [for example, 160 cm2/Vs in Science 390, eaea0849 (2025)]. Given that mobility is purportedly limited solely by phonon scattering, I request a detailed comment on why the measured mobility not only fails to approach the intrinsic theoretical limit but also underperforms relative to prior reports.

Reviewer #2

(Remarks to the Author)
This revised manuscript has largely addressed my previous questions. With the new experimental data, the proposed “self-aligned” mechanism appears much more reliable and convincing. However, before publication, I have one minor suggestion: the authors should correct their adsorption-energy definition to Ead = (EMoS2/sapphire – (EMoS2 + Esapphire))/S .

Reviewer #3

(Remarks to the Author)
I appreciate the authors' further efforts and explanations, and I recommend acceptance of the revised manuscript. Congratulations to the authors for identifying the self-aligned and self-limiting vdW epitaxy effect and revealing its

mechanism.

Open Access This Peer Review File is licensed under a Creative Commons Attribution 4.0 International License, which permits use, sharing, adaptation, distribution and reproduction in any medium or format, as long as you give appropriate credit to the original author(s) and the source, provide a link to the Creative Commons license, and indicate if changes were made.
In cases where reviewers are anonymous, credit should be given to 'Anonymous Referee' and the source.
The images or other third party material in this Peer Review File are included in the article's Creative Commons license, unless indicated otherwise in a credit line to the material. If material is not included in the article's Creative Commons license and your intended use is not permitted by statutory regulation or exceeds the permitted use, you will need to obtain permission directly from the copyright holder.
To view a copy of this license, visit https://creativecommons.org/licenses/by/4.0/

Reply to the Reviewers

We would like to express our sincere thanks to all the reviewers for these precious comments concerning our manuscript. These comments are all valuable for revising and improving the quality of the manuscript. We have carefully revised the manuscript according to the comments/suggestions of the reviewers. Please find a marked copy of the revised manuscript that shows changes made on the revision highlighted in color.

Reply to first reviewer

The words and sentences corrected are **highlighted in red** in the manuscript.

Reviewer #1: *The manuscript authored by Yoshiki Sakuma et al. reports the epitaxy of wafer-scale single crystalline MoS_2 monolayers on c-plane sapphire via a self-aligned coalescence mechanism. The authors claim that although the initial nucleation produces domains of various orientations, these rotationally misoriented domains are deterministically self-aligned and merged into energetically favorable 0° domain during coalescence, yielding a continuous, unidirectional single-crystal. The mechanism elucidated by the authors is interesting and fundamentally differs from previous results. Although the present work provides novel insights into the synthesis of wafer-scale single-crystalline TMDCs films, the central point (i.e., the underlying mechanism) lacks strong evidence to support it. Therefore, I cannot recommend its publication in Nature Communications, at least for the present.*

Reply: We sincerely appreciate the reviewer's constructive feedback and thoughtful evaluation of our manuscript. We carefully address the points raised in the revision process.

Comment 1: *The authors claim that although the initial nucleation produces domains of various orientations, the low-angle twisted and antiparallel 60° domains, upon impingement of the domains in lateral, started to merge into the energetically favorable 0° domain to form a single crystal, driven by the adsorption energy difference. There are several key issues here that need to addressed in detail by the authors.* ***First(1),*** *given the adsorption energy difference, why does the initial nucleation not yield the most energetically stable domains, but instead generate domains with various orientations? This result contradicts most of the previous results.* ***Second(2),*** *why does the adsorption energy difference only come into effect when domains with different orientations come into contact?* ***Third(3),*** *c-plane sapphire comprises six repeated slabs with ABABAB stacking in a bulk unit cell along the z axis [Figure below, adopted from Nature Nanotechnology. 18, 1289–1294 (2023)]. Is the sapphire step a mono-step height (~0.2 nm) corresponding to alternating slabs of A and B? If this is the case, the 0° orientation is the most energetically stable on slab A, while the 60° orientation is the most stable on slab B. Therefore, even if the authors' argument is correct, it is impossible to obtain a single-crystalline MoS_2 wafer under such circumstances because of the coexistence of slabs A and B (i.e., 0° and 60° orientations).*

Reply: Thank you for your detailed comments. We address each point below.

(1) In thermal statistical distributions, the occupation probability of the lowest energy state does not reach unity; rather, it follows the Maxwell–Boltzmann distribution, whereby energetically stable orientations exhibit higher occupation probabilities. This situation is illustrated in the potential energy landscape in the following figure (a), in which multiple local minima exist as a function of twisted angle, separated by energy barriers. The following table (b) presents the calculated strain induced by lattice mismatch and cell size as a function of twist angle through a supercell model. In addition, we calculated the volume fraction for each orientation from the peak area intensities obtained by in-plane XRD. Basically, orientations with smaller lattice mismatch exhibit larger volume fractions, whereas energetically unfavorable orientation appear with very small volume fractions. Importantly, in the initial stage of nucleation, it is considered that nuclei with the minimum cell size of 10.89° first determine the epitaxial relationship. Although this configuration is energetically unfavorable, the experimental observation that 10.89° grains remain in sufficient quantity even after 20 minutes of film growth suggests that they do not transition to the most stable 0° domain by

overcoming the energy barrier. This is attributable to the fact that the energy barrier increases as the nucleus size becomes larger. Consequently, the relationship between volume fraction and energy does not follow a simple proportionality. We note that such misoriented domains, while less frequently emphasized in the literature, have been confirmed experimentally in earlier XRD and TEM studies, as well as optical and AFM studies [ACS Nano, 2015, 9, 4611; Nanotechnology, 2018, 29, 055706; ACS Nano, 2021, 15, 2532; 2D mater., 2023, 10, 045024.]. These discussions have been incorporated into **Fig. S6.**

(a)

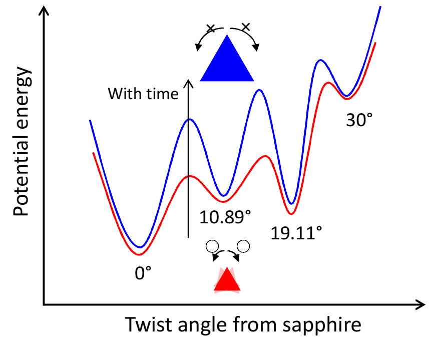

(b)

Super cell			
Twist angle (°)	Mismatch (%)	Cell size (Å)	Volume fraction (%)
0	-0.368	9.48	81.9
10.89	+1.461	8.24	17.3
19.11	+0.420	12.64	0.60
23.41	-3.492	13.78	NA
30	-4.129	15.81	0.17
16.10	-4.276	16.43	NA

(2) If the rotation of a misoriented nucleus toward the 0° orientation is considered, the atomic displacement required is relatively large and cannot be accomplished through thermal fluctuations alone. In contrast, once these domains come into contact with the energetically most favorable 0° domains, the domain boundaries migrate to form 0° domains. In this process, the atomic displacement required for boundary migration is considerably smaller.

(3) Regarding the comparison with *Nature Nanotechnology* 2023, 18, 1289, we would like to point out that Tung group consider the O-sublattice, while we consider the Al-sublattice, as shown in the following (c) and (d). Tung group obtained 30° orientation by preparing the AA slab in miscut wafer of c/a = 1°(O-sublattice) in order to avoid 30°/90° antiparallel domains. On the other hand, if we consider the Al-sublattice, 0° orientation is always most stable orientation. This explanation was added as in **Fig. S17**. Moreover, we performed AFM observations at the initial growth stage (4 min) to directly identify the orientations of MoS_2 triangles. At this stage, the sapphire substrate preserved its characteristic single-step height. The AFM image in the following figure (e) shows that 0° and 60° domains can coexist within terraces of the same height, as indicated by red and blue arrows. This observation demonstrates that the MoS_2 orientation in our system is not determined by alternating A/B slabs of the sapphire substrate. This explanation was added as in **Fig. S3**.

(c)

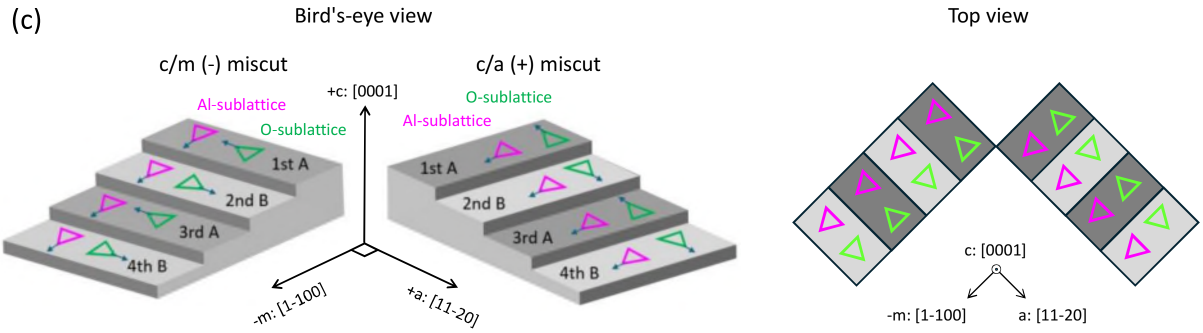

(d)

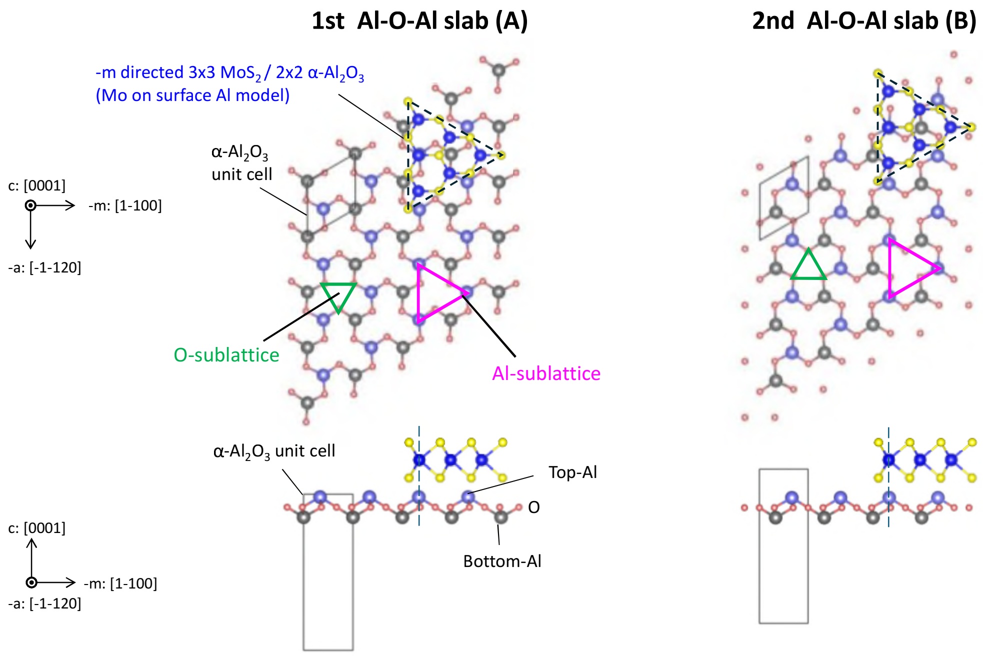

(e)

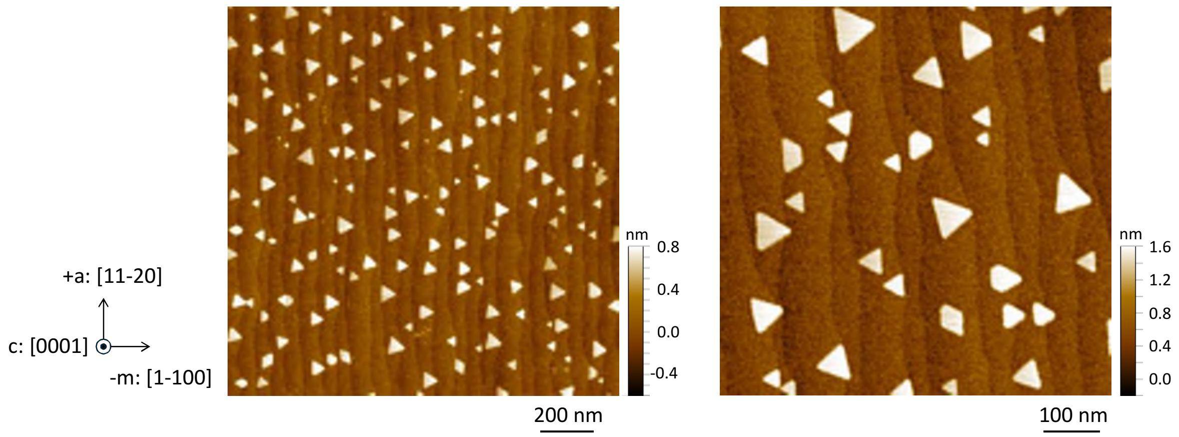

Comment 2: *Fig. 1d is the core evidence supporting the authors' argument, but it is difficult to validate the conclusion that the wafers are single-crystal because the TEM characterization is limited to extremely small local areas. The authors should perform wafer-level characterization, with at least 10 or more different locations randomly selected. In addition, I'm a little skeptical of the percentage data for different orientations in Fig.1d. At 35 min, the 0° domain is 48.5%, while the 60° domain is 9.44%. This is very confusing and contradicts the results of Fig. 1b. The authors should give more than 10 AFM data like Fig. 1b, which can enable us to clearly obtain the percentage of different orientations and cross-verify with the TEM data in Fig.1d.*

Reply: Thank you for your critical yet constructive comments. To substantiate our claim of self-aligned single-crystal formation, we have additionally acquired ten differential DF-TEM images, as presented below. These results clearly demonstrate single-crystalline domains with only minimal occurrences of antiparallel and twisted orientations across the entire 3 mm ϕ TEM grid. Furthermore, in-plane XRD measurements can detect misoriented domains in over a 19 mm ϕ area. The spot area of LEED measurements is ~1 mmϕ and 10 mm range. These data also support the wafer-scale single-crystal formation. This explanation is added in **Page 8 and Fig. S10**.

Regarding the apparent discrepancy between Fig. 1b and Fig. 1d, we clarify that Fig. 1b presents the **quantity ratio** (based on the number of domains) of different orientations based on AFM analysis, whereas Fig. 1d shows the **area ratio** (based on the occupied area of domains) derived from TEM measurements. This distinction is now explicitly emphasized in **Page 5** and **figure caption of Fig. 1d**.

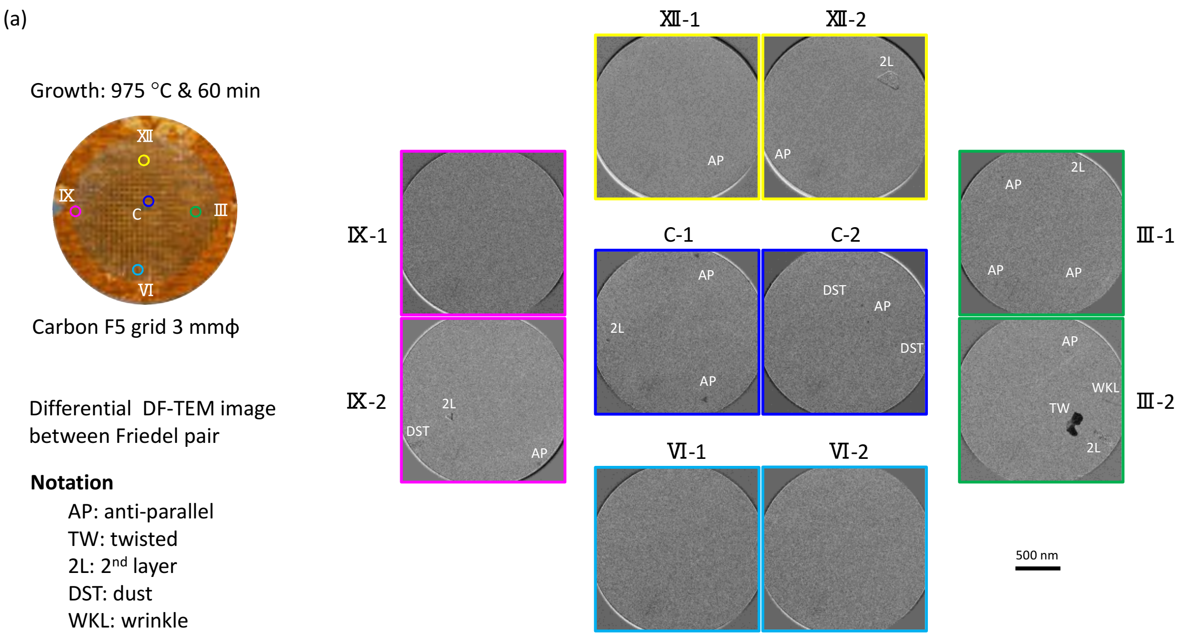

Comment 3: *From Fig. S6, although diffraction spot intensities associated with the reciprocal lattice vectors ka are different from those corresponding to kb, the difference is quite small. Could such a small difference be masked by experimental error, leading to erroneous conclusions such as the percentage of 0° and 60° domains?*

Reply: The difference between diffraction spot intensities for ka and kb is theoretically ~10% [Ultramicroscopy, 2020, 215, 113019.], which is sufficiently high to distinguish ka and kb. The following figures compare the previous report with our present case. Both shows similar difference, which is indeed large enough to distinguish ka and kb. For clarity, eye guide was also added in **Fig. S8(c)**.

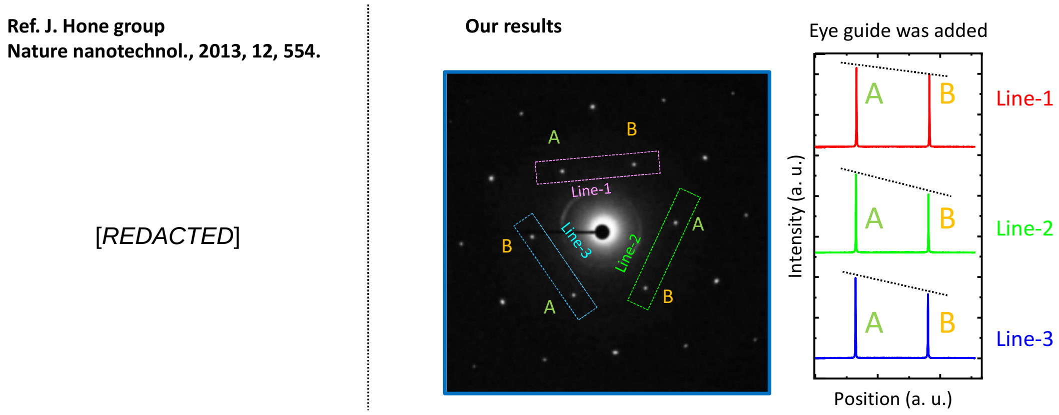

Comment 4: *For the sentence "About ~55% of domains exhibited 0° and the remainder was 60°. Upon closer inspection, however, ~11° and ~49° domains were also discerned, as shown by blue dotted lines", the preceding and following parts contradict each other. For the preceding part, there are only two orientations, i.e., 0° and 60°. While the following part highlights that there are other orientations. The authors should do a statistic to point out the percentage of different orientations.*

Reply: Thank you for your comments. The previous explanation was insufficient. We intended to emphasize the quantity ratio of the two domains, as explained in the reply to comment1. Therefore, the statement "About ~55% of domains exhibited 0° and the remainder was 60°" was replaced with "In terms of the quantity ratio of domains, approximately 55% exhibited 0°, with the remainder at 60°." as noted on **Page 5**.

Comment 5: *For sentence "the subpeak with 10.89° twisted angle was resolved into a doublet at 10.4° and*

11.3° (average: 10.85°)", how is the angle value of 10.89° extracted? The angle value of 10.89° is not the specific angle of these two peaks, nor is it the average value. In fact, the authors also used the angle value of 11° prior to this sentence. The inconsistent angular values are extremely confusing.

Reply: Thank you for your critical reading. Twisted angle of 10.89° in **Fig. S6** was adapted from theoretical value calculated from the supercell model. On the other hand, experimentally determined values were 10.42° and 11.32° and their average is 10.87° (10.85° was not correct.). Therefore, theoretical value of 10.89° was revised to experimental average value of 10.87°. These modifications were conducted in the main text (**Page 6**). **Fig. S6** was also revised.

Comment 6: *From Fig. 1b, it can be known that the terrace width of the sapphire surface increased significantly. For Fig. 1b, the terrace width after growing at 950° is over 200 nm. What is the terrace width after growing at 975° or higher?*

Reply: Thank you for your comments. The following AFM was obtained for sample grown at 975 °C for 70 min. For c-plane sapphire wafer with c/m = -0.2° of miscut angle, the single step height and the terrace width for 1ML height are c/6 = 0.217 nm and approximately 62 nm, respectively. According to the height profile along the white line, several kinds of terrace widths appear depending on the step heights. Therefore, when the growth temperature is higher than 950 °C, the terrace width increased to ~200 nm with 3ML height. This data was added in **Fig. S4**.

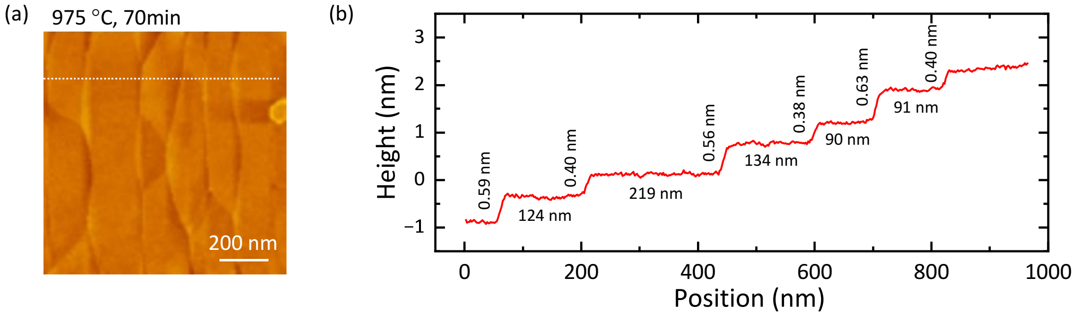

Comment 7: *In page 7, the authors write "upon impingement of the domains in lateral at approximately 40 min, the low-angle twisted domains, followed by the antiparallel 60° domains", what data does the authors have to support that the coalescence 60° domains occurred after the low-angle twisted domains?*

Reply: We have accumulated multiple experimental observations suggesting that low-angle twisted domains tend to disappear before the antiparallel 60° domains, leading eventually to a single-crystal film. However, upon carefully re-examining all available data, we concluded that the current evidence is insufficient to definitively establish the sequence of disappearance between these two types of domains. Accordingly, we have revised the manuscript to state "Upon impingement of the domains in lateral at approximately 40 min, the low-angle twisted and the antiparallel 60° domains started to merge into the energetically favorable 0° domain." In **Page 7**.

Comment 8: *For Fig. S9, it seems that only the curve growing at 975° has no twisted domains. For the curve growing at 1000°, it is quite same with that at 950° and 900°, showing a broad tail structure at the foot of the 0° peak and also twisted domains. This does not seem to be the case, as the authors claim, that higher temperatures favor single-crystal formation. This also seems inconsistent with the mobility results in Figure 4.*

Reply: Thank you for your critical reading. As we mentioned in the main text **P14 (highlighted in red, bottom)**, MoS_2 properties were characterized on 2×2 cm^2 sapphire wafers, while devices were fabricated using 2-inch wafers. The 2-inch wafers require ~50 °C higher substrate temperatures due to different MOCVD susceptors. The growth temperature of 1000 °C is too high for 2×2 cm^2 sapphire wafers. So, their surface became rough, resulting in the degradation of MoS_2 grown at 1000 °C. This can be seen as broad tail structure at the 0° peak in in-plane XRD of **Fig. S13**. In case of 2-inch wafer growth, this surface roughness of sapphire substrates appeared at 1050 °C. These explanations were also added in **the figure caption of**

Fig. S13.

Comment 9: *For SHG mapping in Fig. 1f, the intensity is quite non-uniform. In addition, the scale is too large, spanning five orders of magnitude (from 0 to 10^5). The authors must tune the scale to within an order of magnitude. Any sample is homogeneous as long as the scale is large enough. For right panel of Fig. 1f, how many SHG curves at different locations are superimposed?*

Reply: Thank you for your comment. Regarding the "apparent" intensity non-uniformity in the SHG map of Figure 1f, as shown in the figure below, we had previously used discrete scale when constructing 3D maps. However, by adopting continuous scale, the "impression" changes significantly, although we obtained highly uniform SHG map, as before. We have therefore adopted continuous scale. In addition, the polarization dependence of SHG measured at different 25 points are shown below, which also demonstrate uniformity of monolayer MoS_2. These data were revised in **Figure 1f** and some additional analysis was added in **Figure S14**.

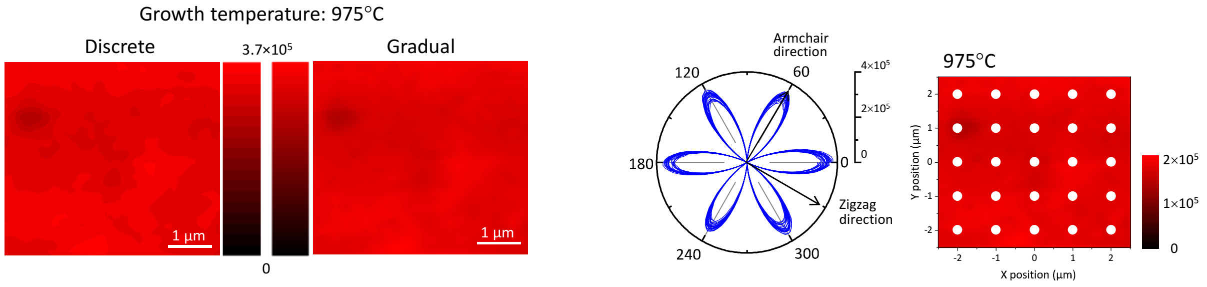

Comment 10: *In Fig. 2b and Fig. S11, why are there some irregular mosaic backgrounds in the LEED images in addition to the diffraction spots?*

Reply: We really appreciate this comments since we did not realize this problem. As shown below, the problem is due to the reduction of resolution during automatic PDF conversion. Therefore, **Fig. 2b** and **Fig. S15** were carefully checked after PDF conversion. In this revision, this issue was solved. Thank you.

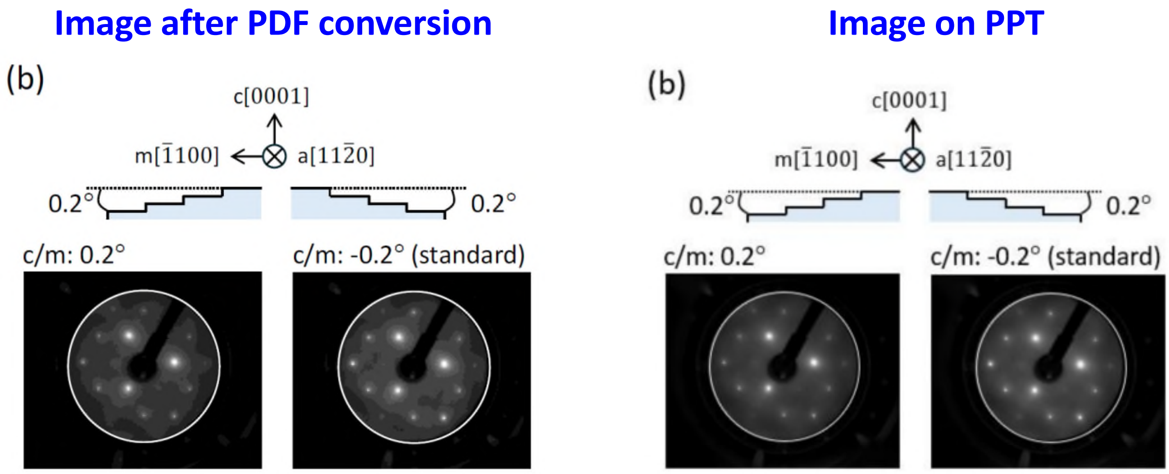

Comment 11: *To support the self-limiting growth, the authors should show more AFM images. For each growth time (over four different growth times), the authors should present AFM images of more than 10 random positions on the wafer.*

Reply: Thank you for your comment. In this revision, AFM images were obtained again to support the self-limiting growth. As clearly seen in the following AFM images, The area number density of the second-layer domains is suppressed, remaining limited to only a few domains per μm^2 even at the growth duration of 150 min. Since the island height is approximately 0.6 nm, the second layer has formed, and islands higher than three layers have not been observed. This data was added in **Fig. S23**. Moreover, absorbance measurements for MoS_2 grown at 975 °C were also conducted, which was included in **Figure 3a**.

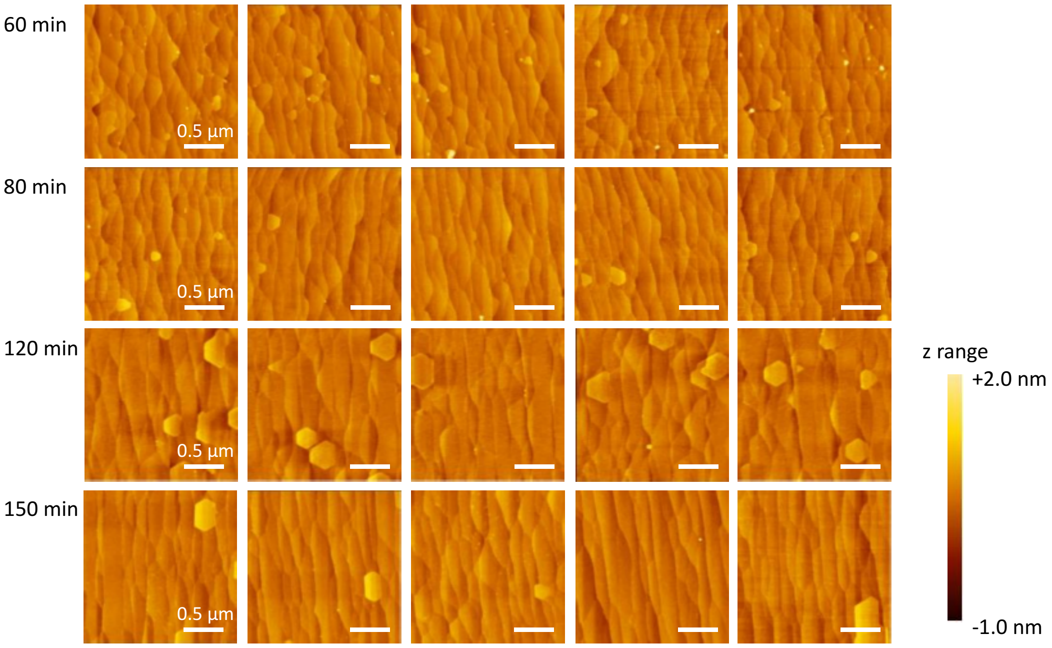

Comment 12: *For the sentence "High-resolution in-plane XRD ϕ scans at five different points in the wafer, as shown in Figure 3d, macroscopically revealed the formation of uniform film without low angle and antiparallel domains throughout the wafer", the XRD data cannot demonstrate that there are no antiparallel domains.*

Reply: Thank you for indicating our miswriting. Since XRD cannot separate antiparallel domains, the word of "antiparallel domains" was removed.

Comment 13: *For transfer curves (Fig. 4b) and output characteristics (Fig. 4c), why do the authors use samples grown at two different temperatures? They should standardize the samples and present data from a unified sample.*

Reply: Thank you for comments. Since the highest mobility was obtained for the sample grown at 1025 °C. The output curves were replaced with the results obtained from the sample grown at 1025 °C, as shown below. (**Figure 4c**)

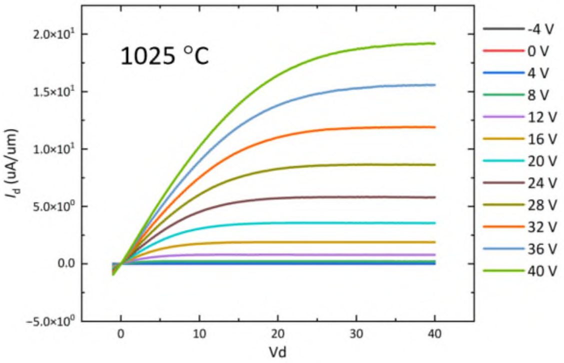

Comment 14: *In page 14, the authors state: "mobilities achieved via MOCVD are comparatively lower than those obtained using powder CVD, rendering small comparisons of μ values at room temperature less meaningful". I cannot agree with the authors' view. Mobility is an intrinsic property of a sample, which is not determined by the growth method. If the mobility of samples obtained by a growth method is lower than that of other methods, this method has no application value and should be eliminated. The authors should*

explain why the mobility of MOCVD samples is comparatively lower than those obtained using powder CVD—whether it is due to too many defects in the samples or other reasons.

Reply: We apologize for the insufficient explanation. Currently, the mobility of MoS_2 films grown by MOCVD is much lower than that of films grown by powder-source CVD. Thus, comparing small differences in room-temperature mobility (e.g., 25 vs. 32 $cm^2/V \cdot s$) is of limited significance, since such values alone cannot clarify the transport mechanism. In our device measurements, we have clearly demonstrated differences in the transport mechanisms based on the temperature dependence of mobility. Accordingly, our intention was to emphasize the importance of carefully evaluating the temperature dependence, rather than focusing on small variations in the mobility values. This emphasis is particularly crucial because, to our knowledge, the temperature dependence of the mobility in MoS_2 films grown by MOCVD has not been reported. Based on this reasoning, we have revised the relevant sentence in **Page 14** of the manuscript.

For the reason why MOCVD mobility is lower than powder CVD mobility, it is not clear at present. However, it should be pointed out that the highest powder CVD mobility was obtained in the heterostructure with *h*-BN [nature commun., 2024, 15, 1825.], not SiO_2. Strictly speaking, the direct comparison is not fair. Nevertheless, we feel that the defects density may be different since the growth conditions are different, for example, the initial grain size for MOCVD is much smaller than that for powder CVD. These discussions were added in **Page 15**.

Comment 15: *The authors claim the phonon limited electron mobility, I also do not agree with this because we cannot simply draw this conclusion directly from the temperature evolution of mobility. The temperature evolution can only indicate whether phonons come into play. In addition, the obtained mobility is lower than the phonon limit value.*

Reply: This is also due to the insufficient explanation. Kaasbjerg [PRB, 2012, 85, 115317.] predicted that mobility of monolayer MoS_2 in vacuum can be limited by optical phonon in MoS_2 for the temperature around room temperature and temperature coefficient of $\gamma = 1.69$ in $\mu \sim T^{-\gamma}$. However, this is drastically changed when MoS_2 is placed on different substrates because surface optical phonon of substrate is involved in the carrier scattering in MoS_2 through the equation $\Gamma \propto \omega_{so} exp(-\hbar\omega_{so}/k_B T)$, where $\hbar\omega_{so}$=22, 59, 100 meV for HfO_2, SiO_2, *h*-BN, respectively. Because of surface optical phonon scattering, when HfO_2 is used as back gate oxide, the mobility is generally reduced. This is true for graphene case. However, HfO_2 is often used as back gate oxide for mobility measurement of MoS_2 and high mobility is often obtained. In contrast, MoS_2 encapsulated in h-BN exhibit $\gamma = 1.9$ [nature nanotechnol. 2015, 10,534.], which is higher than theoretical expectation. Therefore, at this moment, the limiting factor for temperature dependence of mobility for MoS_2 on substrates has not been clarified yet. The situation is further complicated by the role of dielectric screening from high-κ oxides. In our present case, MoS_2 is placed on SiO_2/Si substrate and γ increases with improving the crystallinity of MoS_2, as shown below. Based on this discussion, in this revision, the above discussion and γ values for different growth temperatures were added in **Fig. S28**.

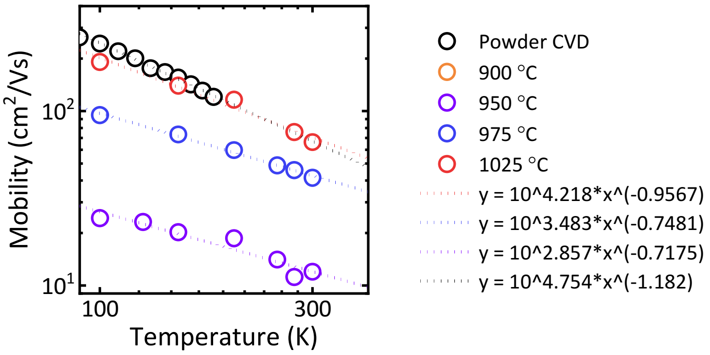

Comment 16: *Regarding "attains a record-high mobility, approximately 66 cm²/Vs", I suggest the authors remove the claim of "record-high".*

Reply: We have removed "record-high" from the abstract and main text.

Comment 17: *Regarding "approaches values similar to those achieved previously with powder CVD46", I suggest the authors cite the most recent literature. Ref. 46 cited by the authors is already 8 years old and does not represent state-of-the-art results.*
Reply: Thank you for your comment. We have checked literatures again. When we limit the mobility of 1L-MoS_2 on SiO_2/Si substrate for fair comparison, indeed, ref. 46 reports highest mobility so far. Of course, there are some other papers with higher mobilities, but all data use heterostructure with *h*-BN.

Comment 18: *For the second paragraph in the main text "Since c-plane of bulk sapphire exhibits……and other techniques", the logic of the writing is very confusing. First, the authors state in the first sentence that based on basic symmetry guideline it should be possible to realize MoS_2 single crystals on sapphire. However, in the second sentence immediately following, the authors go on to say that simply on the basis of basic symmetry guideline, the experiment did not realize MoS_2 single-crystal. Are the authors trying to illustrate the basic symmetry guideline in the first sentence, or are they trying to show by the second sentence that the basic symmetry guideline is wrong now? Subsequently, the authors highlight that two major strategies known as step edge-guided and symmetry-guided epitaxy have been proposed. In fact, both strategies are within the framework of basic symmetry guideline (i.e., the first sentence).*
Reply: We appreciate the reviewer's comment and acknowledge that the original explanation was insufficient, which may have led to confusion. From a purely symmetry-based perspective, heteroepitaxy of a threefold-symmetric monolayer MoS_2 on a threefold-symmetric c-plane sapphire (bulk as well) should, in principle, yield a single crystal under equilibrium conditions. (This is also discussed in reply to comment1) In practice, however, growth occurs under nonequilibrium conditions, and pioneering work by Kis group did not achieve such single crystallinity. While it might be theoretically possible to suppress antiparallel domains by drastically reducing supersaturation and growing at an extremely slow rate, the growth rate would become impractically low for any realistic application. Consequently, previous studies have introduced approaches such as substrate miscut and step-edge guidance to promote single-crystal growth. We have revised the Introduction **Page 2&3** accordingly to clarify this point simply.

Comment 19: *For the third paragraph in the main text, it is also very confusing. This is because the authors raised many key scientific problems in this passage, but then the authors don't address any of them.*
Reply: As correctly noted by Reviewer #3, our growth process is robust and reproducible because it places less stringent requirements on the orientation of the initial MoS_2 nuclei. In the Introduction, we highlighted two central scientific challenges: (i) the inherent difficulty of controlling the atomic structure of the sapphire surface precisely, and (ii) the possible modification of the sapphire surface, namely ,buffer layer formation, induced by sulfur precursors and other process gases.
In this study, we prepared sapphire substrates with miscut angles of c/m = ±0.2° and c/m = -1° (not shown in this manuscript) and c/a = 0.2° (for c/a data, please see the reply to comment4 of reviewer#2). Under these conditions, domain coalescence proceeded successfully, ultimately leading to single-crystal MoS_2. With respect to buffer layer formation, the underlying mechanism remains under debate; therefore, we intentionally refrained from making definitive claims in this manuscript. By focusing the discussion specifically on the control of sapphire surface atomic structure, we demonstrate that our growth mode does not require precise tuning of the miscut direction or angle, thereby ensuring robustness and reproducibility. These clarifications and the scope of our conclusions have now been explicitly incorporated into **Page10 (middle)** of the revised manuscript.

Comment 20: *In line 21, the authors write about "quasi-van der Waals (vdW) epitaxy". What does "quasi" mean? I am confusing for the use of word "quasi".*
Reply: As indicated, explanation was insufficient. We define vdW epitaxy for 2D growth on 2D material and quasi-vdW epitaxy for 2D growth on 3D substrate. Therefore, this explanation was added in **Page 2 Line19**.

Comment 21: *For "LEED intensity - voltage", there are two redundant spaces.*
Reply: Thank you. Corrected **(Page 9)**.

Comment 22: *Regarding Raman mapping of Fig. 3e, why does it resemble a scatter plot?*
Reply: This was also the issue of scale, where more than 5 colors were used. In this correction, 2 colors were used. Uniformity is clearly seen. **Fig. 3e** was replaced.

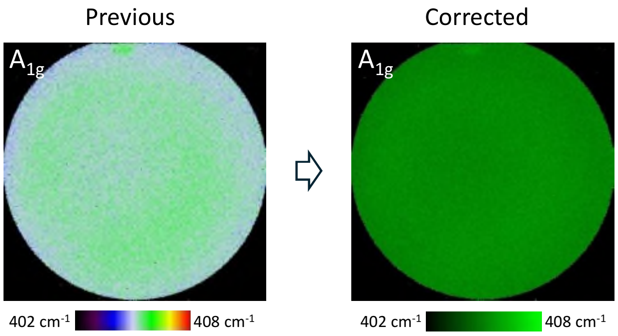

Reply to second reviewer

The words and sentences corrected are **highlighted in blue** in the manuscript.

Reviewer #2: *The manuscript reports a compelling growth concept wherein misoriented monolayer MoS_2 domains undergo a self-aligned coalescence process to yield single-orientation films on c-plane sapphire via MOCVD. The authors provide experimental support using in-plane XRD, differential dark-field TEM, 4D-STEM, and SHG mapping, and further demonstrate promising field-effect mobility. While the concept of grain boundary (GB) annihilation through epitaxial grain coalescence is interesting and potentially impactful, several aspects of the mechanistic interpretation, statistical characterization, and device-level validation remain insufficiently addressed.*
I believe this work has merit but, in its current form, I do not recommend publishing this work in Nature Communications. Below, I outline my major concerns:
Reply: We sincerely appreciate the reviewer's thoughtful and positive evaluation. We carefully address the points raised in the revision process.

-Mechanism Uncertainty
Comment 1: *The manuscript would benefit from a more detailed description of the MOCVD process, particularly regarding the timing of precursor delivery and shutdown, as well as the cooling protocol. It appears that all in-plane XRD and structural characterizations were performed post-growth, meaning that the samples had undergone a full cooling stage prior to measurement.*
This raises an important mechanistic question: why does the transition to single-orientation crystallinity appear to occur sharply around the 40-minute growth mark? While the authors attribute this transition to in-situ domain coalescence and grain boundary migration, an alternative possibility is that some degree of nucleation and growth occurs during the cooling stage. At that point, the system may lack sufficient thermal energy to drive proper orientation, leading to the formation of metastable, misoriented domains.
Furthermore, the observed suppression of new misoriented domains at later growth stages (e.g., at 40 min) may not reflect an intrinsic self-alignment mechanism, but instead result from reduced available surface area for new nucleation events.
Reply: Thank you very much for comments. As shown by the following figure, in our MOCVD process, the Mo precursor (MoO_2Cl_2) supply is intentionally terminated 30 seconds before the onset of cooling stage. This is a key advantage of MOCVD, where the timing of precursor flow can be precisely controlled. After stopping the Mo precursor, H_2S continues to flow until the substrate temperature decreases to 200 °C, which serves the purpose of preserving MoS_2 from thermal decomposition and/or healing sulfur vacancies. This protocol effectively prevents additional nucleation or growth during the cooling stage, since Mo supply has already been cut off. Therefore, the possibility that misoriented domains are formed during cooling is highly unlikely. Moreover, the reviewer pointed out that nucleation of misoriented domains at later growth stage is suppressed due to reduced available surface area, which may give "apparent" self-limiting behavior. However, when using the conventional $Mo(CO)_6$ precursor, bilayer growth proceeds irrespective of reduced available surface area. In contrast, the phenomenon we discovered with the MoO_2Cl_2 precursor is not the suppression of new domain formation, but rather coalescence and annihilation of pre-existing twisted and antiparallel domains, as confirmed by differential DF-TEM images. Thus, the sharp transition to a single-orientation state around 40 minutes is best explained by in-situ domain coalescence and grain boundary migration during active growth, rather than by any post-growth effects during cooling. The description of the MOCVD process and above-mentioned explanation were added in **Fig. S2.**

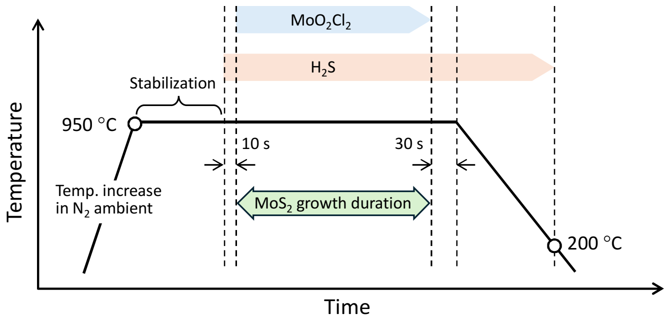

Comment 2: *Would the timing of this crystallinity transition shift if the supply rates of MoO_2Cl_2 and H_2S were varied? A parametric study would clarify the role of precursor flux in the coalescence mechanism.*
Reply: We have not yet conducted detailed experiments regarding the behavior when changing the timing of precursor supply. If the Mo flow rate is increased to enhance the growth rate, the onset of coalescence will be advanced; however, if the growth rate becomes excessively high, the self-aligned process may not finish before the full coverage, potentially resulting in incomplete single crystallization. We consider that the timing of precursor supply may be a key factor distinguishing our results from previous studies. However, this will be our future work.

Comment 3: *The author believes that their unidirectional MoS_2 with 0° alignment is due to the interface energy between MoS_2 and sapphire. However, both their DFT calculations (Fig. S15) and prior literature (Refs. 7, 8, 13, 37) consistently suggest that the 30° configuration is thermodynamically more stable. Moreover, Figure 2a does not clearly reveal a distinct "buffer layer" that could alter this behavior. The authors should reconcile these inconsistencies and provide a more convincing explanation.*
Reply: Thank you for comments. Let me clarify our orientation results in conjunction with those of other groups, as shown below. Left figure is our DFT simulation (Fig. S15). Larger adsorption energy for MoS_2 on sapphire substrate indicates more stable situation, that is, 0°. Therefore, our experimental results, the coalescence to 0° domain, is consistent with the DFT simulation. However, as the reviewer indicated, other groups report different results and they proposed different mechanisms, as summarized below. The main purpose of this manuscript is not to reveal the growth mechanisms but to show self-aligned growth through the multifaceted experimental results. Our conclusion is that self-aligned growth occurs through the most stable epitaxial relationship (0°). Moreover, this is related with **comment1(3) from reviewer #1**. Therefore, please refer to it.
Regarding the reviewer's suggestion about the "buffer layer" in the cross-sectional HAADF-STEM image of Figure 2a, we acknowledge that our explanation in the main text has been limited, as it is extremely challenging to obtain direct evidence on this issue. Nevertheless, based on our recent cross-sectional HAADF-STEM results for the MoS_2/sapphire interface, we have observed that the buffer layer on the sapphire substrate decreases with increasing growth temperature. This observation is consistent with our finding that single-crystal formation is enhanced at higher growth temperatures, since MoS_2 can directly "see" the sapphire surface structure without a termination layer, thereby promoting epitaxial growth.
In this revision, because it is difficult to provide conclusive results at present, we have limited our statement to: "Previous studies have discussed the presence of a buffer layer on the sapphire substrate. In our case, this buffer layer exhibited weaker HAADF-STEM contrast than in previous report, which likely facilitates the formation of an epitaxial relationship between MoS_2 and sapphire." **(Page 9)**

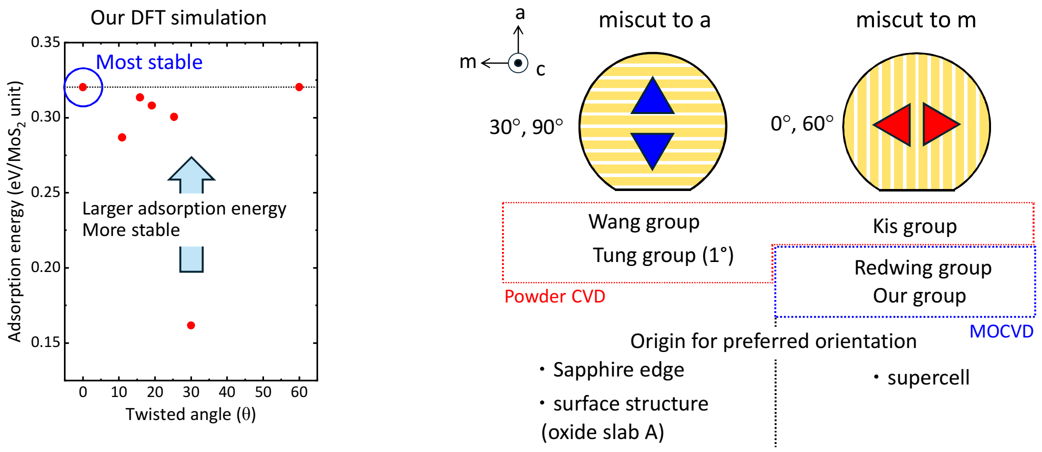

Comment 4: *Although the manuscript claims orientation is not governed by step edges, the sapphire substrates were intentionally annealed to create step-terrace structures. The authors should clarify this*

contradiction. In the meantime, the author should try other sapphire planes (e.g., A-plane, M-plane) or miscut direction (e.g. C to A plane) to confirm the MoS_2 lattice is not governed by step edges.

Reply: The surface of as-received sapphire substrates usually exhibits polishing scratches. Therefore, annealing was performed to flatten and clean the sapphire surface. Moreover, MOCVD growth on the a-plane and m-plane was not included in the present study because their surface symmetries differ significantly from that of the c-plane. On the other hand, to address the reviewer's concern, we conducted additional experiments using miscut substrates toward the a-direction on the c-plane. Since the edge structures in the +a and –a directions are equivalent, only the +a direction was prepared, and MOCVD growth was performed under our optimized condition of 975 °C. As shown below, the LEED pattern for the substrate with c/a = 0.15° was consistent with those obtained for c/±m. These results clearly demonstrate that the epitaxial orientation is not governed by edge structures, but by the surface structure (or symmetry) of c-plane sapphire substrate. The corresponding data have been included in **Fig. S15**.

(d) Summary of LEED patterns for different miscut directions

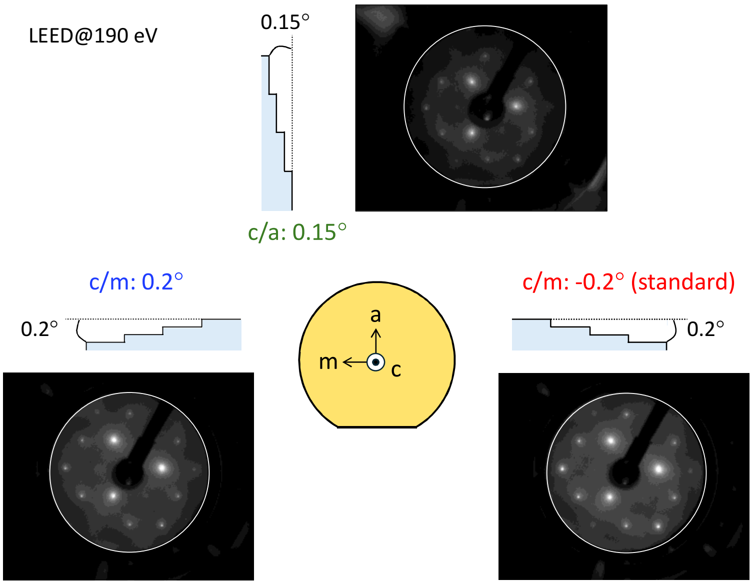

Comment 5: *The AFM images presented in Figures S2c, 1b, and 3b reveal that the sapphire step structure evolves significantly with growth time and temperature. Such step morphology changes—particularly under high-temperature conditions exceeding 950 °C—could plausibly influence domain orientation selection, nucleation behavior, and coalescence dynamics.*

I encourage the authors to discuss the potential impact of these evolving step features on the orientation control mechanism proposed in the manuscript. In particular, the work by Zheng et al. (Nature Communications 14, 592 (2023)) provides relevant insights into step-edge reconstruction on sapphire at elevated temperatures and its influence on epitaxial alignment of TMDCs. That study emphasizes the importance of growth timing and surface evolution in controlling domain orientation, which appears to share similarities with the present work.

Reply: Thank you for your insightful comment and for highlighting the work by Zheng et al. (Nature Communications 14, 592 (2023)). The study by Zheng et al. indeed provides an important perspective on how step-edge reconstructions on sapphire surfaces contribute to epitaxial alignment during the nucleation stage. However, our present work focuses primarily on the coalescence stage following nucleation, which marks a key distinction between the two studies. While changes in step and terrace morphology during high-temperature MOCVD growth are unavoidable, especially due to the chemical reactivity of H_2S. Nevertheless, we would like to emphasize that cross-sectional HAADF-STEM in Figure 2a, as discussed in comment #3, shows us the limited buffer layer on sapphire substrate, which facilitates the epitaxial relationship between MoS_2 and sapphire. This indicates that the coalescence of grains in our study is governed more strongly by the epitaxial relationship between MoS_2 and sapphire surface, rather than by the dynamic step-terrace morphology. These discussions were added in **Page 10**.

-Single Crystallinity Assessment

Comment 6: *While DF-TEM (Fig. 1d) reveals local orientation, the sampling area is too limited to convincingly support wafer-scale single-crystallinity. The authors should provide more statistically significant data, possibly via multi-area DF-TEM.*

Reply: Thank you for your valuable comment. This is consistent with **comment2 from reviewer #1**. Therefore, please refer to it.

Comment 7: *Could strain or tilt introduced during the transfer process affect DF-TEM interpretation? Additionally, are there high-resolution images available showing grain boundary structures between 0° and 60° domains? In the 35-minute sample (Fig. 1d), some flakes visually appear antiparallel, yet DF-TEM indicates they are all 0°.*

Reply: In terms of effect of strain or tilt on DF-TEM, in 2D crystals, the electron diffraction intensity is elongated in the direction perpendicular to the crystal plane, forming a rod-like shape. Consequently, specimen tilting is expected to have little effect on the relative intensities between diffracted waves that contribute to image formation. Furthermore, the violation of Friedel's law originates from the anisotropy of the atomic scattering factor (i.e., the lack of spherical symmetry), which is not influenced by strain or tilt. Therefore, even in the presence of strain or tilt, the differences in relative intensities between diffracted waves arising from the violation of Friedel's law are considered to remain unaffected.

In terms of high-resolution atomic image of grain boundaries, in this study, we focus on the annihilation of grain boundaries based on differential DF-TEM method. Therefore, in this revision, we additionally took differential DF-TEM more than 10 locations (reply to comment 6). We just started to observe the grain boundaries during the domain coalescence and annihilation by TEM. We will report a very detailed study on the grain boundaries in the next paper.

For false color image (Fig. 1d), the reviewer considers that some flakes visually appear antiparallel, yet DF-TEM indicates they are all 0°. Since grain shape itself does not tell us the accurate orientation, our orientation assignment relies on the differential DF-TEM criterion above, not on flake shape. Please see the following DF-TEM, whose contrast was largely enhanced. It is clear that 0° domains can be distinguished from 60° domains.

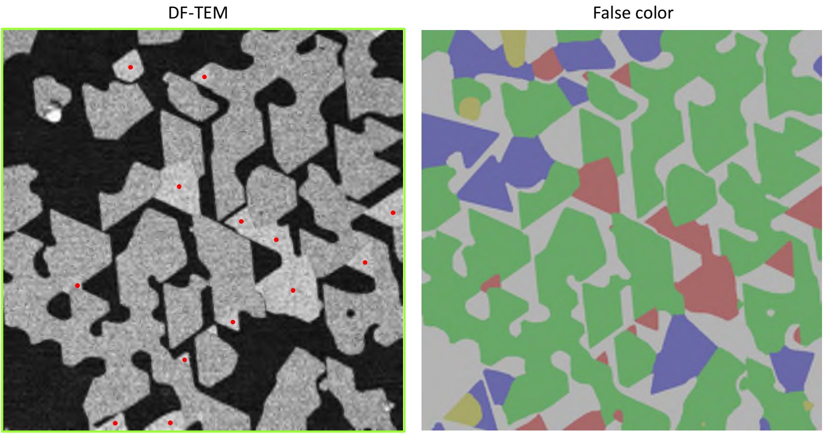

Comment 8: *The grain size is reported to be ~200 nm, while the SHG laser spot size typically exceeds 1 μm. The observed intensity fluctuations in Fig. 1f and Fig. S10 suggest the presence of multiple domains within each spot. Therefore, SHG mapping may not be sufficient to conclude a reduction in grain boundaries without supporting high-resolution data. To me, the provided mapping image contains many grain boundaries.*

Reply: Thank you for your comment. We note that this concern is similar to **Comment 9 from Reviewer #1**. Please refer to our detailed response there.

-Self-limiting monolayer

Comment 9: *The claim of self-limited monolayer growth requires further scrutiny. In Figure 3b, the AFM images clearly show bilayer triangular MoS_2 domains, which contradicts the assertion of monolayer-only*

growth. Moreover, the Raman A_{1g} peak variation of ~2 cm^{-1} in Figure 3e aligns with the known difference between monolayer (Δ = 19 cm^{-1}) and bilayer (Δ = 21 cm^{-1}) MoS_2, suggesting that non-negligible bilayer regions are present across the wafer.
While MoO_2Cl_2 provide slower kinetics compared to $Mo(CO)_6$, reduced growth rate alone does not confirm a self-limiting mechanism. To convincingly establish self-limitation, the authors should perform a systematic study by increasing the MoO_2Cl_2 supply or extending the growth time to demonstrate that no additional layer forms under higher precursor flux or prolonged exposure.
Reply: Thank you for your comments. In terms of self-limited monolayer growth, this question is the same as **comment11 from reviewer#1**. Please refer to it.
In terms of Raman A_{1g} peak variation of 2 cm^{-1}, this variation does not come from the existence of bilayer. The Raman spectra across the wafer was shown below. It is clear that difference between E^1_{2g} and A_{1g} is 19.8 cm^{-1}, which indicates monolayer MoS_2. These data was added in **Fig. S24**.

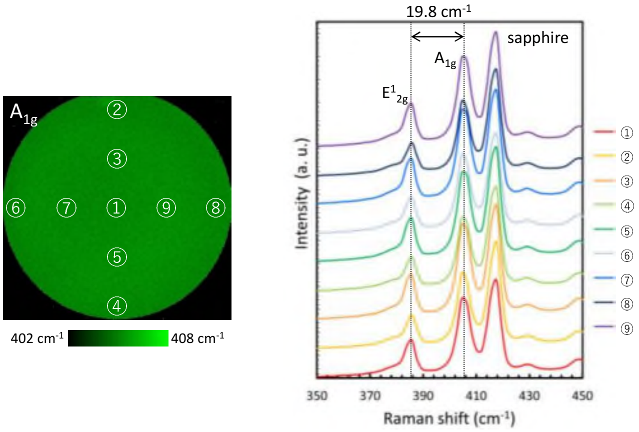

Moreover, in terms of effect of the increase in Mo precursor supply on self-limiting behavior, by increasing the N_2 flow rate into the MoO_2Cl_2 container, the supply of the Mo precursor was enhanced, and MOCVD growth was carried out at the growth conditions of 60 min and 850 °C, as shown below. The absorbance at the C-exciton peak was plotted as a function of N_2 flow rate into the MoO_2Cl_2 container. This apparent saturated tendency with an increased Mo precursor supply provides compelling evidence for the self-limiting behavior. This clearly indicates that the growth saturation is not limited by a reduction in growth rate originating from the slower kinetics of MoO_2Cl_2 precursor. This data was added in **Fig. S21**.

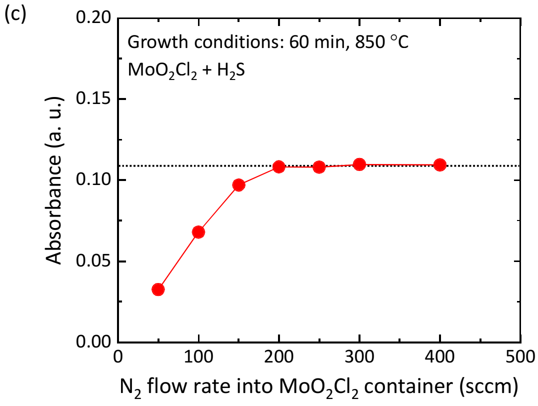

-Inhomogeneous device performance
Comment 10: *In Figure 4, the authors present electrical characteristics from several batches of MoS_2 FETs. However, the data exhibit substantial device-to-device variation—exceeding three orders of magnitude in current density (Figure 4b) and showing large mobility spread (Figure 4e). This high degree of inhomogeneity raises concerns about the claim of wafer-scale single crystallinity.*
Reply: We apologize for the insufficient explanation in the original submission. In **Fig. 4b**, the I_d–V_g data for all channel lengths (from 100 μm down to 1 μm) are included in a single plot. To address the reviewer's concern, we have revised the figure so that the curves are color-coded according to channel length, which

makes it clear that the apparent variation arises from the systematic increase in current as the channel length decreases, as shown below. Thus, the large spread in current density is not due to inhomogeneity among devices of identical geometry, but rather because devices with different channel lengths were plotted together. Moreover, some variation in mobility comes from the PMMA transfer and variation of contact resistance since two terminal devices were measured to obtain the average for mobility.
Fig. 4b was replaced.

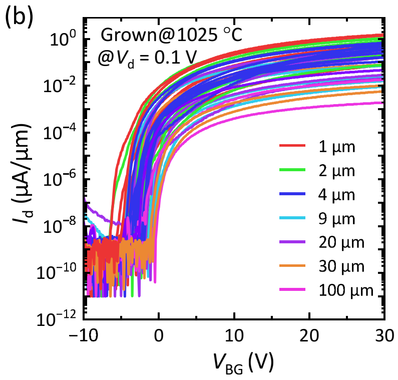

General concerns
Comment 11: *Different characterizations were performed on samples grown at different temperatures—e.g., thickness analysis at 850 °C, grain mapping at 975 °C, and devices at 1025 °C. The rationale for these choices should be explained. Is the epitaxial mechanism or material quality consistent across these temperatures?*
Reply: Thank you for your comment. As you suggested, the growth temperature is important factor. Let me explain this using in-plane XRD shown below (**Fig. S13**). At the growth temperature is low, the single crystallization due to the coalescence was insufficient. With increasing the growth temperature, it was enhanced and the best temperature is 950~975 ℃. Based on this, most of data are shown for 975 ℃. Exception "in the previous submission" was absorption measurements to reveal the self-limiting behavior, which was indeed 850 ℃. Therefore, absorption measurements at 975 ℃ was also included in **Fig. 3a** in this revision.
In contrast, for the devices, the best temperature was 1025 ℃. (This is the same question as the comment 8 from reviewer#1.) As we mentioned in the main text **P14 (highlighted in red, bottom)**, MoS_2 properties were characterized on 2×2 cm^2 sapphire wafers, while devices were fabricated using 2-inch wafers. The 2-inch wafers require ~50 °C higher substrate temperatures due to different MOCVD susceptors. Therefore, as shown below, the growth temperature of 1000 °C is too high for 2×2 cm^2 sapphire substrates. So, their surface became rough, resulting in the degradation of MoS_2 grown at 1000 °C. This can be seen as broad tail structure at the 0° peak in in-plane XRD. In case of 2-inch wafer growth, this surface roughness of sapphire substrates appeared at 1050 °C. These explanations were also added in **the figure caption of Fig. S13**.

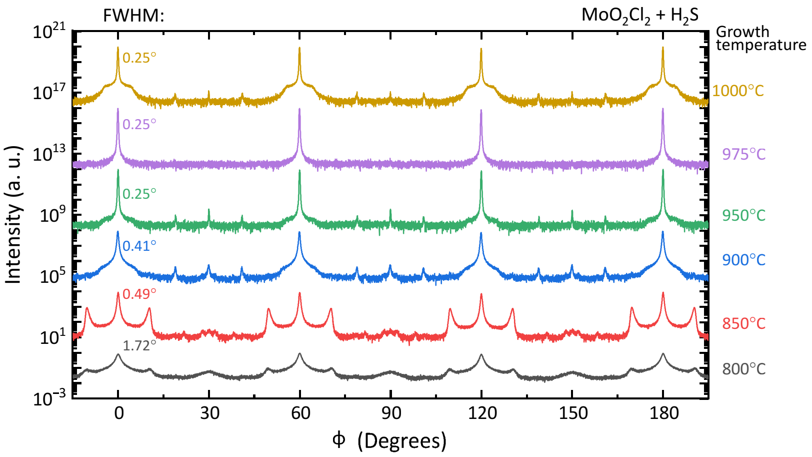

Comment 12: *The manuscript would benefit from additional language polishing and restructuring for clarity*

and flow.
Reply: We have revised our manuscript through the language editing service.

Reply to third reviewer

The words and sentences corrected are **highlighted in green** in the manuscript.

Reviewer #3: *The growth of single-crystalline monolayer TMDC films is highly desired for their lab-to-fab transition. Focusing on this objective, the manuscript "Self-aligned and self-limiting van der Waals epitaxy of monolayer MoS_2 to annihilate grain boundaries for scalable 2D electronics" reported an MOCVD growth approach for achieving large-area monolayer MoS_2 single crystals, based on the self-alignment of initially-misoriented domains. The growth of MoS_2 also occurs layer-by-layer, leading to good spatial uniformity and high electronic performance.*
In my opinion, the growth mechanism revealed in this work is very interesting and is fundamentally different from previous efforts. It might be even more robust/reproducible, if the growth process can be fully understood and controlled, since there is less stringent requirement on the orientation of the initial MoS_2 nuclei. With that being said, I think there are several important questions to be addressed to better elucidate the growth mechanism and understand why this particular growth process is governed by a totally different mechanism from previous ones:
Reply: We sincerely appreciate your time and effort in reviewing our manuscript. We are grateful for your positive evaluation. Your comments are highly encouraging to us.

Comment 1: Sapphire substrate requirements: *By comparing the LEED patterns of MoS_2 grown on c-sapphire with opposite miscut angles (Fig. 2c), the authors concluded that the alignment of MoS_2 is governed by the terrace instead of the step edges. Does this mean that this growth approach is also compatible with conventional c-sapphire or different miscut directions? Further discussion or experimental evidence would be helpful. Additionally, the authors are suggested to perform AFM characterization on the MoS_2 flakes at an initial growth stage to examine whether the nucleation mainly occurs at step edges or terraces.*
Reply: Thank you for your comment. We note that this concern is similar to **Comment 4 from Reviewer #2**. Please refer to our detailed response there.
In addition, to directly address the reviewer's suggestion, we performed AFM observations at the initial growth stage to identify the preferred nucleation sites. The AFM images show that MoS_2 nuclei can form at various locations—such as the center of terraces, along step edges, or near the upper edges of steps—without any consistent positional preference. Furthermore, in contrast to some previous report [nature nanotechnol. 2023, 18, 1289.], we observed that 0° and 60° domains can coexist within terraces of the same height. These results clearly indicate that orientation control in our growth process is not governed by step edges. These discussion was added in **Note of Fig. S3**.

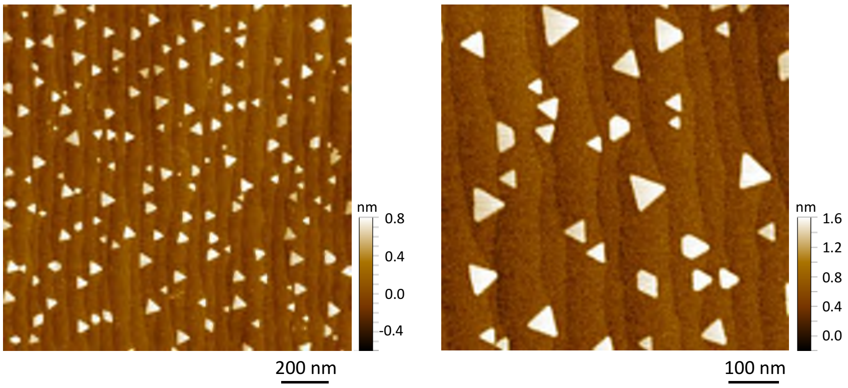

Comment 2: Formation of isolated domains of twisted MoS_2: *In Fig. 1c and S4, in-plane XRD observes the preferential twist angles of isolated MoS_2 domains (e.g., in the 20 min growth sample), and the angles match with MoS_2/sapphire lattice model. However, the formation energy of these twisted domains should be significantly higher than that of 0-degree and 60-degree domains (as shown in DFT calculation results in Fig. 2e and S15). Why do the twisted domains form in this particular growth approach? It would be necessary to discuss the mechanism and also compare it with existing work.*

Reply: Thank you for your comment. This is consistent with **First(1)** question in **comment1 from reviewer #1**. Therefore, please refer to it.

Comment 3: MoS_2 domain alignment mechanism: *Two possible mechanisms for domain alignments are compared, including the rotation model and grain boundary migration model. The authors identified that the grain boundary migration is a more plausible mechanism. However, why does the grain rotation not happen at the early nucleation/growth stage? DFT calculations are desired to compare the activation energies for domain rotation versus GB migration. Moreover, due to the similar free energies of 0-degree and 60-degree domains, I think the conversion from 60-degree to 0-degree domain requires not only a large activation energy, but also a precise control of growth thermodynamics, which should be very challenging. The authors are suggested to discuss further how this growth approach achieves good controllability.*

Reply: In the early growth stage of MoS_2, particularly at an extremely small size of around 1 nm, domain rotation may occur. However, as the domain size increases, such rotation will be suppressed. This is because, in large triangular domains, atoms near the vertices would have to travel an exceptionally long distance to accommodate rotation. Regarding DFT calculations, evaluating the energetics of domain rotation in MoS_2 would require explicitly defining the edge structures of triangular domains. In practice, there are numerous possible edge terminations and reconstructions, each potentially altering the activation energy. Considering this large variability, it is currently difficult to conduct a meaningful and comprehensive comparison between domain rotation and grain boundary migration based on DFT. For this reason, we have not performed such calculations in the present revision.

Comment 4: Temperature-dependence of self-aligned coalescence: *The authors identified that the domain alignment works for growth temperatures above 900 C. However, I think it is also possible that the growth rate and grain boundary migration rate are lower at low temperatures, so that additional growth time is needed for the full merge of MoS_2 domains and for the self-alignment.*

Reply: Thank you for your insightful comment. The following figures are DF-TEM images for samples grown for 60 min at (a) 850 °C and (b) 975 °C, respectively. Since self-aligned coalescence did not finish before fully covered growth at 850 °C, polycrystalline feature was observed. As reviewer suggested, even at a relatively low temperature of approximately 850 °C, self-aligned growth could potentially occur if the growth duration is extended beyond 60 min. However, our DF-TEM observations tracking the conditions under which self-alignment proceeds suggest that the plausible growth mechanism is the sweeping of grain boundaries driven by diffusion when two domains come into contact. Based on this mechanism, self-aligned growth at lower temperatures would require impractically long growth durations.

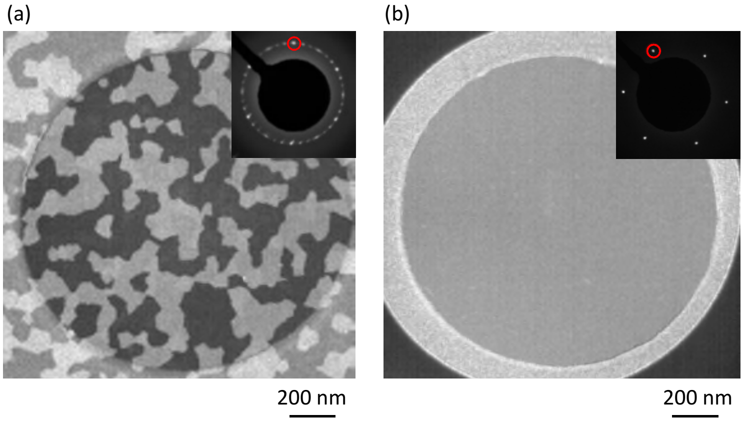

Comment 5: Mobility extraction of the MoS_2 device: *The devices used for measuring the electron mobility have quite long channels (tens of micrometers), which are not comparable to the practical application scenario of 2D nanoelectronics. For example, Ref. 48 uses a short-channel device (200 nm), which can lead to lower mobility values but is closer to the practical application. The authors are suggested to fabricate short-channel devices to better estimate and benchmark the mobility.*

Reply: Thank you for your comment. We have studied short-channel devices with different channel lengths from 2 μm to 51 nm fabricated by EB lithography (transfer length method (TLM)), as shown below. It is clear that drain currents increased with reducing the channel lengths. Based on the TLM, the contact resistance was estimated as approximately 150 kΩμm. Consequently, the mobilities of the short-channel devices, while measured to be on the order of a few cm^2/V·s, primarily reflect the influence of contact resistance rather than limitations in channel quality. These discussions were added in **Page 14 and Fig. S26**.

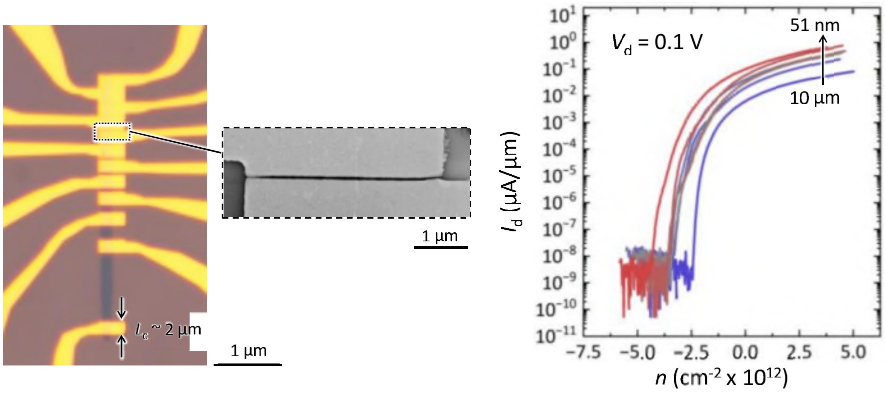

Comment 6: Defect density measurement: *The authors discussed the MoS_2 device performance and the crossover from thermally activated to phonon-limited transport in MoS_2 grown at higher temperatures (Fig. 4d). It would be helpful to directly measure the defect density by using STM, conductive AFM, or STEM, etc., to better correlate with electrical data and compare with previous work.*

Reply: Thank you for your insightful comment. We fully agree that quantifying the defect density in monolayer MoS_2 is critical for a comprehensive understanding of the carrier transport mechanism. Indeed, previous studies, such as the work by the J. Hone group on WSe_2 grown by the flux method [ACS Nano, 2023, 17, 16587], have demonstrated a clear correlation between defect density and carrier mobility. However, on our current study, the primary focus is on eliminating grain boundaries through self-aligned epitaxial growth, and establishing a clear link between macroscopic uniformity and phonon-limited transport. Defect analyses are currently underway in our laboratory and will be reported in future work to further elucidate the microscopic origin of the observed transport behavior.

Comment 7: Batch-to-batch reproducibility of the self-aligned coalescence phenomenon: *On page 7 of the manuscript, the authors mentioned that the time-dependent domain orientation evolution is highly reproducible in MOCVD but inconsistent in powder-source CVD. It is needed to include the data from different growth batches to support this conclusion. For example, error bars for the inset of Fig. 1c are required.*

Reply: We appreciate the reviewer's comment regarding batch-to-batch reproducibility. To demonstrate the high reproducibility of the self-aligned coalescence process in our MOCVD system, we conducted three consecutive sets of MOCVD runs with four different growth durations: 30 min (just before starting partial coalescence), 50 min (during self-aligned coalescence), 70 min (after completing coalescence, #1), and 90 min (after completing coalescence, #2) as shown in the following Fig. a. In addition, to confirm the quantitative accuracy of the XRD peak intensity, another independent run of 20 min and 120 min were performed, and the integrated intensity of the 0° peak of all 14 samples was evaluated to investigate the self-limiting nature of MoS_2 growth. The raw in-plane XRD data from each run show very similar peak shapes and intensities, confirming high reproducibility at the qualitative level. For quantitative comparison, we calculated the integrated intensity of the 0° peak from each dataset (Fig. b). As a measure of variability, we used the coefficient of variation (CV = standard deviation / mean × 100%), as summarized in the accompanying table. The CV values are sufficiently small throughout the growth series, and become even smaller after the coalescence is complete (70 min and 90 min), reflecting improved uniformity. Moreover, the self-limiting behavior can be seen in the following figure b, suggesting the high quantitative accuracy of in-plane XRD. These additional reproducible results are added in **Fig. S9**.

Regarding the inset of Fig. 1c, this plot presents data from a single representative growth batch; therefore, error bars for batch-to-batch variation cannot be meaningfully provided for this specific figure. Instead, we have supplied the additional multi-batch results above to support the reproducibility claim.

(a)

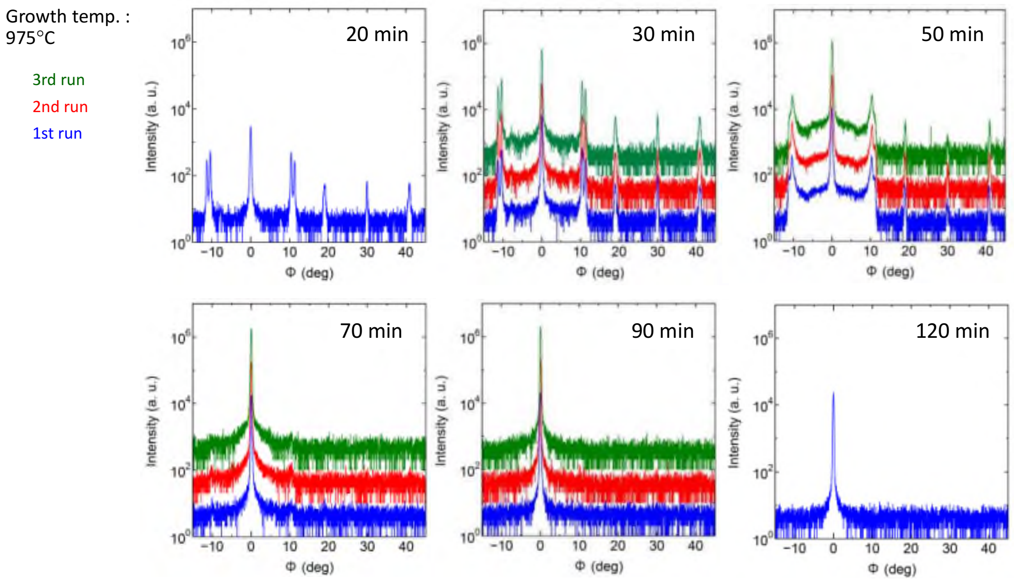

(b)

Growth time (min)	Runs	0° peak integral intensity (counts·deg)	Averaged integral intensity (counts·deg)	Standard deviation (counts·deg)	Coefficient of variation (%)
30	#1	1831	1814.66	94.20	**5.19**
	#2	1692			
	#3	1921			
50	#1	3570	3584.00	214.15	**5.98**
	#2	3329			
	#3	3853			
70	#1	4577	4729.67	108.15	**2.29**
	#2	4798			
	#3	4814			
90	#1	5139	5071.67	53.92	**1.06**
	#2	5007			
	#3	5069			

(c)

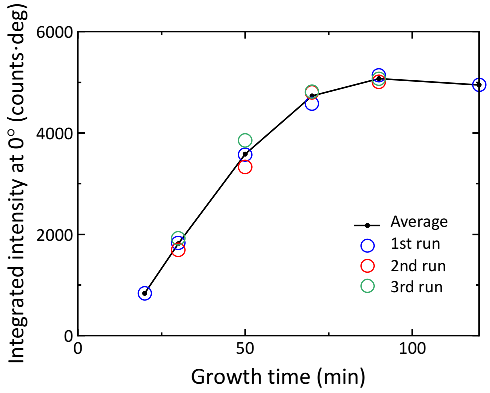

Reply to the Reviewers

We would like to express our sincere thanks to all the reviewers for these precious comments concerning our manuscript. These comments are all valuable for revising and improving the quality of the manuscript. We have carefully revised the manuscript according to the comments/suggestions of the reviewers. Please find a marked copy of the revised manuscript that shows changes made on the revision highlighted in color.

Reply to first reviewer

The words and sentences corrected are **highlighted in red** in the manuscript.

Reviewer #1: *In their response letter and revised manuscript, the authors have largely addressed the comments raised by me. However, for the claim "phonon limited electron mobility at room temperature", I still do not agree. Temperature-dependent measurements can only tell us that which factors dominate the changes in mobility, while they cannot tell us what specifically dominates the mobility at a given temperature. The mobility is typically limited by defect and phonon scattering at room temperature. Since defect scattering is not particularly sensitive to temperature, so the change in mobility with temperature appears to be phonon-dominated. In addition, the full name of word "vdW" is already provided in line 20, so it is unnecessary to repeat it in line 22.*

Reply: We would like to clarify our point in more detail. The following figure shows the temperature dependence of the mobility. As reported in Ref. [3], CVD-grown monolayer MoS_2 encapsulated by h-BN exhibits a clear transition of the dominant scattering mechanism — from phonon-limited in the range of 50–300 K to short-range (defect-related) scattering below 50 K (defect level B). This is the Matthiessen's rule of mobility. In our present case of MOCVD-grown monolayer MoS_2 on SiO_2/Si, a similar trend is observed, although the transition to short-range scattering is less evident due to the limited temperature range of our measurements. Recently, Hone *et al.* [1] reported an extremely high mobility (~5 × 10^4 cm^2/V·s) for monolayer WSe_2, achieved by drastically reducing the defect density (defect level A). When the defect level corresponds to level C, the maximum mobility even at 300 K becomes restricted by defect related scattering. In such high-defect regimes, the mobility exhibits thermally activated behavior with hopping mechanism, consistent with our data. Although the reviewer's comment is reasonable for samples with low to moderate defect densities (levels A and B), in our worse case (level C), hopping behavior of carrier dominates the mobility. Hence, the description "phonon-limited electron mobility at room temperature" remains valid in the context of our sample quality.

As for "vdW," we appreciate the reviewer's note and have removed the redundant full spelling in line 22 since it was already defined earlier. (**Page 2**).

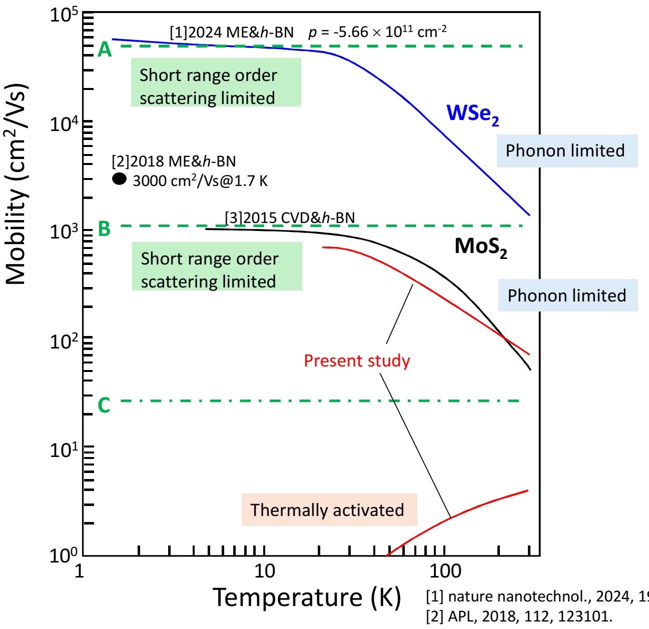

[1] nature nanotechnol., 2024, 19, 948.
[2] APL, 2018, 112, 123101.
[3] nature nanotechnol., 2015, 10, 534.

Reply to second reviewer

<u>The words and sentences corrected are **highlighted in blue** in the manuscript.</u>

Reviewer #2: *Before providing my detailed comments on the revised manuscript, I must note that the format of the author's response letter is difficult to read and follow. This has made the review process more time-consuming. I strongly recommend that the authors reply to each reviewer comment one by one, and clearly indicate the corresponding revisions (e.g., by quoting or pointing to the changed paragraph). In addition, please avoid asking the reviewer to cross-reference other reviewers' comments before fully addressing the current question. It is unreasonable to expect reviewers to repeatedly switch back and forth between different comments.*
In terms of content, I find the most interesting aspect of this work to be the proposal of a new mechanism for achieving single-crystal TMD films. However, in the response, the authors emphasized that "the main purpose of this manuscript is not to reveal the growth mechanisms." This statement is confusing and somewhat contradictory, since without a convincing mechanism the generality of the method remains questionable. There still several key questions remain unclear:
Reply: We sincerely apologize for the format of our previous response letter, which may have made it difficult to follow. Regarding the reviewer's concern about our statement on the growth mechanism, we appreciate the opportunity to clarify. Our intention was not to downplay the importance of understanding the mechanism, but rather to emphasize that a *"complete"* elucidation of the self-aligned and self-limiting growth process is beyond the scope of this first report. Given that this growth mode represents a newly discovered phenomenon, we believe that a comprehensive mechanistic study warrants a separate, intensive dedicated investigation. Nonetheless, in this manuscript, we have provided experimental evidence and discussion that support the proposed mechanism to the extent possible based on our current findings.

Comment 1: *Why is 0° considered the most stable orientation? Normally, lower adsorption energy corresponds to a more stable state. Please clarify your DFT calculation methodology and re-examine your results. (Suggested references: Small 2020, 16, 2000596; ACS Nano 2023, 17 (2), 1196–1205).*
Reply: Thank you for comments. In terms of suggested references, the sapphire surfaces are a-plane with different symmetry in small (2020) and c plane in ACS nano 2023. Therefore, small is excluded in this discussion. Let me clarify our DFT simulation (following left figure). In our DFT simulation, the adsorption energy, E_{ad} is defined as $E_{ad} = (E^{MoS2} + E^{sapphire} - E^{MoS2/sapphire})/S$, where E^{MoS2}, $E^{sapphire}$, and $E^{MoS2/sapphire}$ are the calculated total energies for MoS_2 monolayer, sapphire, and MoS_2/sapphire. S is the area of a superstructure. <u>In this definition, larger adsorption energy for MoS_2 on sapphire substrate indicates more stable situation</u>, that is, 0°. On the other hand, in ACS nano (following right figure), E_{ad} is oppositely defined as $E_{ad} = (E^{MoS2/sapphire} – (E^{MoS2} + E^{sapphire}))/S$. Therefore, as indicated in the following figures, larger negative adsorption energy is more stable. So, our experimental results, the coalescence to 0° domain, is consistent with both DFT simulations.

Our DFT simulation

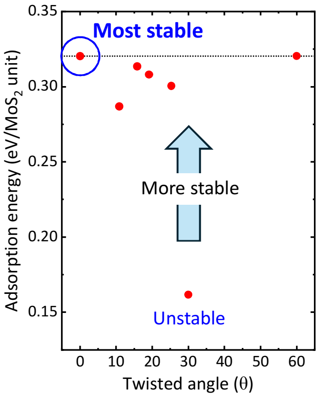

ACS nano, 2023, 17, 1196.

[REDACTED]

Comment 2: *Why does the mechanism occur only after contact or specifically at 40 minutes? In principle, the timing of this crystallinity transition should vary if the supply rates of MoO_2Cl_2 and H_2S are changed,*

since precursor supply affects the timing of contact. Additional experimental evidence is needed to substantiate this claim.

Reply: Thank you for your valuable comment. As pointed out, the onset time (40 min under our standard growth condition) is not a universal value. It is expected to vary depending on the supply rates of MoO_2Cl_2 and/or H_2S, since these parameters affect the lateral growth rate, *i.e.*, the timing of initial contact and onset of coalescence. However, even if the onset time shifts earlier or later than 40 min, such variation alone may not provide direct insight into the essential mechanism. To elucidate the underlying single-crystallization mechanism after coalescence, we examined the time evolution of the false-color maps at very short intervals at 975 °C, as shown in the following figure. This experiment was possible owing to the excellent reproducibility of our MOCVD process, as demonstrated in **Fig. S8**. In the samples for 45 min and 53 min, despite their high MoS_2 coverage, a considerable amount of low-angle twisted and antiparallel domains (red/blue) remain in partial contact with 0° domain. It should be noted that these remaining domains are always accompanied by ungrown regions (black) at their periphery. These results strongly imply that grain boundary (GB) migration might be interrupted at ungrown regions. In other words, sustained GB migration toward a unidirectional single crystal appears to proceed only when individual misoriented domains become fully enclosed by 0° domain, that is, when the GB network becomes continuous and no longer interrupted by ungrown regions.

This consideration, based on our observations, suggests that GB migration also contains tangential motion along the GB, causing the enclosed misoriented-domains to shrink and eventually vanish. This process begins locally as the coverage of MoS_2 on the sapphire increases, and then gradually expands to the entire surface. After 60 min, when MoS_2 fully covers the substrate, the self-aligned coalescence is completed across the entire wafer, resulting in wafer-scale single crystallization. This progression likely explains why full coverage and wafer-scale single crystallization occur nearly simultaneously at around 60 min. Therefore, under our growth conditions, the local surface coverage required to form fully enclosed regions is first achieved at approximately 40 min. This time point can be regarded as the apparent "threshold" for observing the onset of self-aligned coalescence in our experimental condition. This new data was added in **Figure 2c** and the above discussion was added in **Page 11-12**.

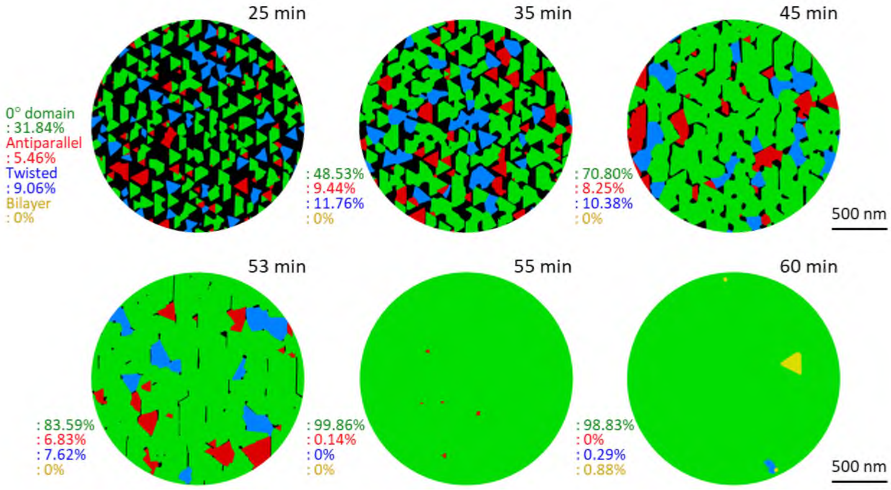

To further support the proposed growth mechanism, we conducted an additional experiment in which the growth was interrupted at 50 min by terminating the MoO_2Cl_2 flow while the H_2S annealing was continued. As shown in **Fig. (A)**, even when the H_2S annealing time was extended up to 70 min after the 50 min growth, further coalescence and annihilation of small-angle twisted domains into 0° domain was not detected by in-plane XRD. In contrast, when the growth continued without terminating the MoO_2Cl_2 flow until the end of growth time (up to 90 min), as shown in **Fig. (B)**, the self-aligned growth was clearly observed after 60 min. These results indicate that partial contact between domains alone is insufficient to drive GB migration by curvature-driven grain growth mechanism; continuous GB migration occurs only after the precursor gases are continuously supplied and low-angle twisted/antiparallel domains become completely enclosed by neighboring 0° domain.

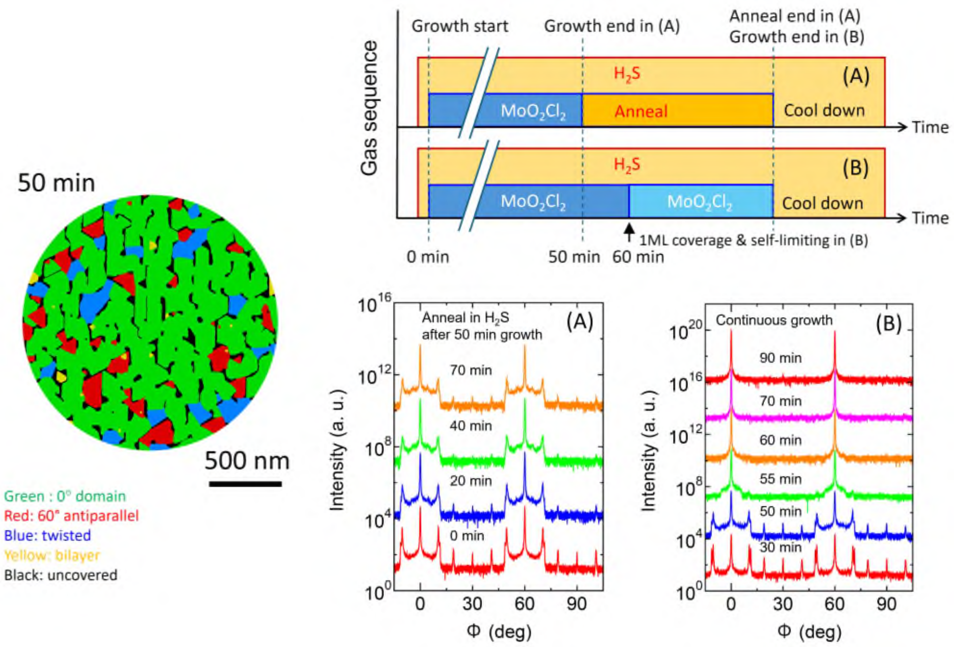

Here, a key characteristic of GB migration during the above-mentioned self-aligned single-crystallization process is that the GB moves not only in the normal direction but also along its tangential direction. Recently, the importance of GB migration in grain growth and recrystallization processes has also been recognized in two-dimensional materials, and both experimental and simulation studies have been actively conducted in the following references. It has been pointed out that a simple curvature-driven mechanism—i.e., mass transport solely based on grain curvature—is insufficient to explain many observed phenomena. Moreover, GB migration has been reported to involve coupled normal motion (perpendicular to the boundary) and tangential motion (parallel to the boundary), described as a shear-coupled mechanism. Nevertheless, GB migration is an extremely complex phenomenon; its dynamics and atomistic details are still far from being fully understood, and substantial further research is required.
This new data and the above discussion were added in **Supplementally Fig. S18**.

References

- Ren, X. and Jin, C. Grain boundary motion in two-dimensional hexagonal boron nitride, ACS Nano 14, 13512 (2020).
- Waters, B. and Huang, Z. F. Grain rotation and coupled grain boundary motion in two-dimensional binary hexagonal materials, Acta Materialia 225, 117583 (2022).
- Taha, D., Mkhonta, S. K., Elder, K. R. and Huang, Z. F. Grain boundary structures and collective dynamics of inversion domains in binary two-dimensional materials, Phys. Rev. Lett. 118, 255501 (2017).
- Azizi, A. et al. Dislocation motion and grain boundary migration in two-dimensional tungsten disulfide, Nat Commun 5, 4867 (2014).

Comment 3: *How is this process related to the substrate? The authors suggest that higher growth temperatures (e.g., 1000 °C), which are believed to cause surface roughening, lead to degraded crystallinity. Could direct evidence for this roughening be provided, and could the authors further explain how surface roughness impacts crystallinity? In this context, it would be highly valuable to demonstrate results on A-plane or M-plane sapphire. This would help clarify whether substrate orientation guides the growth or whether the observed behavior is truly a self-aligned phenomenon. More experimental support is required to strengthen this point.*

Reply: We appreciate the reviewer's valuable comments. Based on optical microscopy observations after MOCVD growth at 1000 °C, we found that the surface morphology exhibited slight changes compared to that observed at lower temperatures. However, these subtle variations were not readily discernible in optical images (difficult to show). To address this, we have included atomic force microscopy (AFM) image of MoS_2 grown at 1000 °C for 60 min on sapphire substrates. This image clearly shows that monolayer MoS_2

did not fully cover the substrate surface under these high-temperature conditions, indicating incomplete self-aligned growth. This is consistent with the in-plane XRD results (**Fig. S13**), which reveal degraded crystallinity. This AFM image was added in **Fig. S12**.

Regarding the substrate orientation, we appreciate the reviewer's suggestion to investigate growth on a-plane and m-plane sapphire substrates. However, unlike the c-plane, these two types of surfaces have two-fold symmetry [Appl. Surf. Sci., 2025, 696, 16292.]. Therefore, achieving unidirectional growth of MoS_2 single crystals on these substrates may not be easy. Clarifying the differences in the growth mechanism from the c-plane would require considerable time and is beyond the scope of this paper, so we believe it would be appropriate to address this as a separate research topic.

@1000 °C, 60 min

AFM

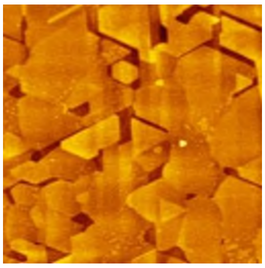

200 nm

Comment 4: *Please avoid overclaiming. For example, the statement: "It is worth noting that the temperature dependence of mobility in MOCVD-grown MoS_2 films has, to the best of our knowledge, not yet been reported." is inaccurate. First, there are already published studies reporting temperature-dependent electrical measurements of MOCVD-grown MoS_2. (Nature Electronics volume 7, pages356–364 (2024)) Second, even if this were not the case, such a statement does not add substantive novelty to this manuscript.*

Reply: The suggested nature electronics paper discussed the temperature dependence of the drain current to evaluate Schottky barrier height at the MoS_2/metal contact, rather than the mobility. While the Redwing and Das groups at Penn State have reported many device characteristics of MOCVD-grown MoS_2, the temperature dependence of mobility had not been reported. However, during the course of this submission, the following paper was published from Redwing and Jariwala group[ACS nano, 2025, 19, 8985.]. Therefore, as suggested, the sentence was changed to "the report on the temperature dependence of mobility in MOCVD-grown MoS_2 films has been considerably limited, compared with MoS_2 synthesized via powder-based CVD methods". **(Page 15)**

Nature electronics, 2024, 7, 356. In Fig. S7

[REDACTED]

Our mobility – Temp data

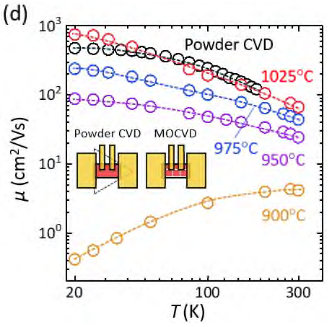

ACS nano, 2025, 19, 8985. In Fig. 4e

[REDACTED]

Reply to third reviewer

The words and sentences corrected are **highlighted in green** in the manuscript.

Reviewer #3: *I appreciate the author's significant effort in responding to my questions and revising the manuscript. Upon reading the revised version, I am more convinced by the observed domain self-alignment phenomenon and the proposed grain boundary annihilation mechanism. The device results presented in the revised manuscript are also helpful to demonstrate the electrical performance of the MOCVD-synthesized film. Nevertheless, there are still several points from my previous comments that I feel the authors should address more adequately. I would like to clarify my questions further, and look forward to the authors' response and revision:*

Reply: We sincerely appreciate your time and effort in reviewing our manuscript. We are grateful for your positive evaluation. Your comments are highly encouraging to us.

Comment 1: *The authors partially addressed my Comments 2-3 by suggesting that the probability of twisted domain formation is governed by Maxwell-Boltzmann distribution, and that the domain rotation is suppressed as domain size increases. However, in my original Comments 2-3, I was also concerned about why the twisted domain percentage in this work (prior to coalescence) is higher than existing reports, and why the self-alignment of 60-degree and 0-degree domains was unobserved in previous growths (e.g., ACS Nano, 2015, 9, 4611). To address these questions and to provide guidance for future efforts on the growth of single-crystalline TMD monolayers, I think it is necessary to provide more discussions on the growth mechanism and clearly compare with existing growth approaches.*

Reply: Thank you for your valuable comment. We consider the following reasons why the fraction of twisted domains (prior to coalescence) in this study appears higher than in previous reports. Accurate evaluation of rotational domains requires in-plane XRD measurements performed "under high-resolution and high-intensity conditions". To the best of our knowledge, no prior study has assessed rotational domains with sensitivity comparable to ours; therefore, earlier works may not have quantitatively captured either the presence or the true areal fraction of twisted domains. For example, M. Chubarov et al. (Nanotechnology 29, 055706 (2018). & ACS Nano 15, 2532–2541 (2021). refs. 5 and 7 in Supplementary) reported small sub-peaks at ~11°, 19°, 28°, and 32° in addition to the main 0° peak using a PANalytical XRD system with a quasi in-plane geometry, as shown in the following **figure A**. However, their X-ray power was 1.8 kW, and the quasi in-plane optical configuration likely yielded insufficient diffracted intensity. In contrast, our measurements were conducted at 9 kW using an optimized in-plane geometry, and the scan speed (typical condition with step = 0.02 deg, scan speed = 0.3 deg/min) was significantly reduced to improve the signal-to-noise ratio (**total time for 180° scan is 10 hours**). As a result, we obtained diffraction intensities of several tens of thousands of counts for the main 0° peak and ~100 counts even for the sub-peaks. We believe that securing this level of intensity is essential for quantitative analysis. A similar observation of rotational domains at around ±11° was also reported by D. Docenovic et al. (2D Mater 10, 045024 (2023). ref. 8 in Supplementary), as shown in the following **figure B**. They evaluated the in-plane rotation-angle distribution of WSe_2 domains grown on c-plane sapphire by MBE using 4D-STEM, and identified rotational domains in addition to the 0° domains. However, because their histogram was derived from a limited local area, the quantitative reliability of the areal fraction remains insufficient.

Taken together, twisted domains are not unique to our samples; they are likely present in MOCVD- and MBE-grown films from other groups as well. The main difference is that high-sensitivity characterization has rarely been performed. We expect that our results will motivate further systematic studies on this issue. These discussions were simply added in **Page 6 & 12**.

***Nanotechnology* 29, 055706 (2018).** ***ACS Nano* 15, 2532–2541 (2021).**

A [REDACTED] [REDACTED]

***2D Mater* 10, 045024 (2023).**

B [REDACTED]

We also consider the following reasons why self-alignment between 60° and 0° domains was not observed in earlier growth studies (e.g., Dumcenco et al., ACS Nano 2015, 9, 4611). Notably, many powder-CVD reports present AFM images showing relatively large domains (~10–100 μm), without resolving the early nucleation regime. To date, no study has systematically and quantitatively traced the full evolutionary pathway—from the emergence and coalescence of smaller, rotated and/or antiparallel early-stage nuclei to the ultimate seamless stitching into large single-crystal domain—through a combined analysis using in-plane XRD, low-magnification DF-TEM, and 4D-STEM. This gap stems in part from the experimental difficulty of conducting time-resolved evolution studies in powder-CVD. Consequently, the existing evidence remains inadequate to support a definitive conclusion.

These discussions were added in the main text (**Page 12**).

Comment 2: *In my previous Comment 4, my intent was to suggest the authors characterize the domain alignment in coalesced MoS_2 grown at lower temperatures (e.g., 850 C). Indeed, the coalescence would take a longer time due to reduced growth rates, but it is still practical in my opinion. I believe this experiment would be meaningful because it could reveal whether the domain alignment is solely constrained by growth time or also by the temperature-dependent grain boundary migration rate. In case if a lower temperature, longer growth time also result in domain self-alignment, the authors should clarify it in the manuscript.*

Reply: We appreciate the reviewer's insightful suggestion. We agree with your idea ideally. However, as the kinetic rate constant follows an Arrhenius-type dependence ($\propto \exp(-Q/kT)$), significantly longer growth times are required at lower temperatures to complete self-aligned growth. The monolayer MoS_2 was grown at 870 °C at different growth times up to 150 min. Then, in-plane XRD was carried out to extract the time evolution of self-aligned growth at the low temperature, as shown in the following Figure (a). It is clear that single crystallization did not occur even at 150 min. Moreover, the XRD total integral intensity between -15° and 45° indicates that full coverage was reached at 60 min and the clear self-limiting behavior after 60 min. Ideally, we can expect single crystallization through self-aligned growth, experimentally, it is not realistic. This data was added in **Page 8&9 and Supplementally Fig. S14**.

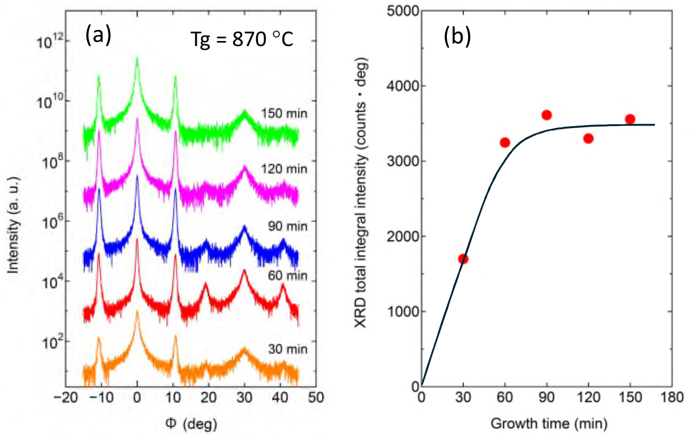

Comment 3: *In the response to my Comment 4, samples grown at 850 C were non-uniform and polycrystalline, but why did the authors present a uniform A1g Raman mapping for the 850 C sample in Fig. 3e? This discrepancy needs to be clarified.*

Reply: Thank you for your comment. Wafer-scale uniformity was achieved owing to the self-limiting behavior. This self-limiting nature was initially verified by absorbance and Raman measurements for monolayer MoS_2 grown at 850 °C (previous **Fig. 3a,e**). Therefore, we initially presented the data at 850 °C. However, as pointed out by the reviewer, 850 °C is relatively lower growth temperature for achieving self-aligned growth, and the film at this temperature is indeed polycrystalline. Accordingly, we have replaced the Raman mapping data in **Fig. 4e** with that obtained at 1025 °C, while the Raman data at 850 °C is now provided in **Fig. S24**.

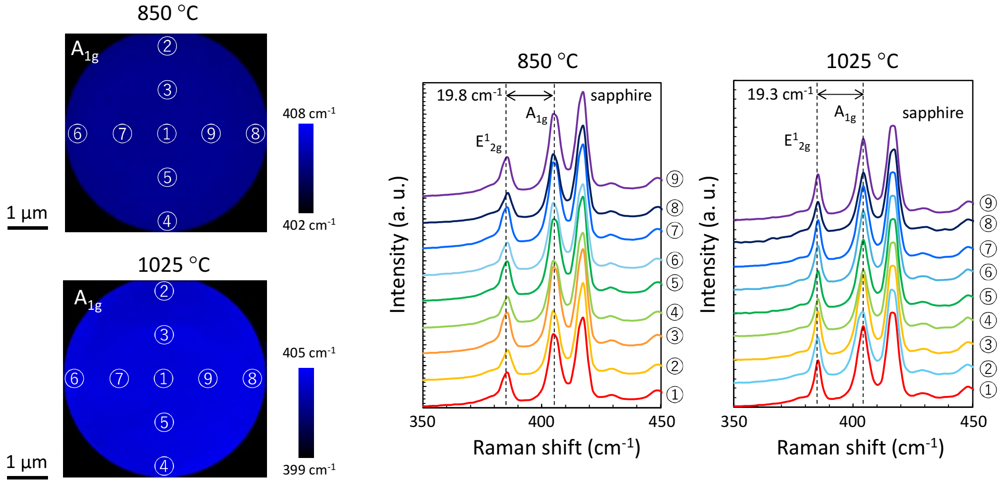

Reviewr#1

I cannot agree the claim "phonon limited electron mobility at room temperature". In the absence of defect scattering or other impurity scattering mechanisms, where only phonon scattering is operative, the measured mobility of monolayer MoS_2 should approach its intrinsic theoretical limit (according to the Matthiessen's rule of mobility 1/μ=1/μ_imp +1/μ_ph). This should represent the highest mobility achievable experimentally in monolayer MoS_2 devices and therefore will outperform all prior reports. Obviously, this is not the case as claimed by the authors. The as-achieved mobility of 66 cm^2/Vs at room temperature is significantly worse than the previous state-of-the-art results [for example, 160 cm^2/Vs in Science 390, eaea0849 (2025)]. Given that mobility is purportedly limited solely by phonon scattering, I request a detailed comment on why the measured mobility not only fails to approach the intrinsic theoretical limit but also underperforms relative to prior reports.

Reply: Regarding the phonon-limited electron mobility, we have discussed this issue with Reviewer #1 three times, including in the present revision. Reviewer #1 has considered the relevant phonons as the intrinsic phonons of MoS_2. However, in the first revision, we mentioned that the phonons in question are not intrinsic phonons of MoS_2, but rather surface optical phonons of SiO_2, as shown in Supplementary Fig. S28. In this case, the experimentally measured mobility is not expected to reach the theoretical mobility limit of MoS_2 at room temperature. In the second revision, our revisions were focused solely on responding to the reviewers' comments, and as a result, we did not sufficiently emphasize that the phonons originate from the surface optical phonons of SiO_2, which led to this discrepancy. Since the primary focus of this manuscript is MOCVD growth, the discussion of transport properties is necessarily limited. Indeed, in the main text, the effect of surface optical phonons of SiO_2 is not discussed and is only addressed in Supplementary Information. Therefore, to avoid further ambiguity, we follow the editor's suggestion: the term "phonon-limited" has been removed from abstract, subsection, main text, and title of Fig. 5. Instead, the temperature dependence of mobility is explained in terms of a typical power-law behavior. **Highlighted in red (Page 4, 15, 16, & 24)**

Reviewr#2

This revised manuscript has largely addressed my previous questions. With the new experimental data, the proposed "self-aligned" mechanism appears much more reliable and convincing. However, before publication, I have one minor suggestion: the authors should correct their adsorption-energy definition to $E_{ad} = (E_{MoS2/sapphire} - (E_{MoS2} + E_{sapphire}))/S$.

Reply: We revised the definition of Ead according to the reviewer's suggestion. **Highlighted in red (Page 26)**

The manuscript authored by *Yoshiki Sakuma* et al. reports the epitaxy of wafer-scale single-crystalline MoS_2 monolayers on c-plane sapphire via a self-aligned coalescence mechanism. The authors claim that although the initial nucleation produces domains of various orientations, these rotationally misoriented domains are deterministically self-aligned and merged into energetically favorable 0° domain during coalescence, yielding a continuous, unidirectional single-crystal. The mechanism elucidated by the authors is interesting and fundamentally differs from previous results. Although the present work provides novel insights into the synthesis of wafer-scale single-crystalline TMDCs films, the central point (i.e., the underlying mechanism) lacks strong evidence to support it. Therefore, I cannot recommend its publication in *Nature Communications*, at least for the present.

Below are my detailed comments:

1) The authors claim that although the initial nucleation produces domains of various orientations, the low-angle twisted and antiparallel 60° domains, upon impingement of the domains in lateral, started to merge into the energetically favorable 0° domain to form a single crystal, driven by the adsorption energy difference. There are several key issues here that need to addressed in detail by the authors. First, given the adsorption energy difference, why does the initial nucleation not yield the most energetically stable domains, but instead generate domains with various orientations? This result contradicts most of the previous results. Second, why does the adsorption energy difference only come into effect when domains with different orientations come into contact? Third, c-plane sapphire comprises six repeated slabs with ABABAB stacking in a bulk unit cell along the *z* axis [Figure below, adopted from Nature Nanotechnology. 18, 1289–1294 (2023)]. Is the sapphire step a mono-step height (~0.2 nm) corresponding to alternating slabs of A and B? If this is the case, the 0° orientation is the most energetically stable on slab A, while the 60° orientation is the most stable on slab B. Therefore, even if the authors' argument is correct, it is impossible to obtain a single-crystalline MoS_2 wafer under such circumstances because of the coexistence of slabs A and B (i.e., 0° and 60° orientations).

[*REDACTED*]

2) Fig. 1d is the core evidence supporting the authors' argument, but it is difficult to validate the conclusion that the wafers are single-crystal because the TEM characterization is limited to extremely small local areas. The authors should perform wafer-level characterization, with at least 10 or more different locations randomly selected. In addition, I'm a little skeptical of the percentage data for different orientations in Fig.1d. At 35 min, the 0° domain is 48.5%, while the 60° domain is 9.44%. This is very confusing and contradicts the results of Fig. 1b. The authors should give more than 10 AFM data like Fig. 1b, which can enable us to clearly obtain the percentage of different orientations and cross-verify with the TEM data in Fig.1d.

3) From Fig. S6, although diffraction spot intensities associated with the reciprocal lattice vectors k_a are different from those corresponding to k_b, the difference is quite small. Could such

a small difference be masked by experimental error, leading to erroneous conclusions such as the percentage of 0° and 60° domains?

4) For the sentence "About ~55% of domains exhibited 0° and the remainder was 60°. Upon closer inspection, however, ~11° and ~49° domains were also discerned, as shown by blue dotted lines", the preceding and following parts contradict each other. For the preceding part, there are only two orientations, i.e., 0° and 60°. While the following part highlights that there are other orientations. The authors should do a statistic to point out the percentage of different orientations.

5) For sentence "the subpeak with 10.89° twisted angle was resolved into a doublet at 10.4° and 11.3° (average: 10.85°)", how is the angle value of 10.89° extracted? The angle value of 10.89° is not the specific angle of these two peaks, nor is it the average value. In fact, the authors also used the angle value of 11° prior to this sentence. The inconsistent angular values are extremely confusing.

6) From Fig. 1b, it can be known that the terrace width of the sapphire surface increased significantly. For Fig. 1b, the terrace width after growing at 950° is over 200 nm. What is the terrace width after growing at 975° or higher?

7) In page 7, the authors write "upon impingement of the domains in lateral at approximately 40 min, the low-angle twisted domains, followed by the antiparallel 60° domains", what data does the authors have to support that the coalescence 60° domains occurred after the low-angle twisted domains?

8) For Fig. S9, it seems that only the curve growing at 975° has no twisted domains. For the curve growing at 1000°, it is quite same with that at 950° and 900°, showing a broad tail structure at the foot of the 0° peak and also twisted domains. This does not seem to be the case, as the authors claim, that higher temperatures favor single-crystal formation. This also seems inconsistent with the mobility results in Figure 4.

9) For SHG mapping in Fig. 1f, the intensity is quite non-uniform. In addition, the scale is too large, spanning five orders of magnitude (from 0 to 10^5). The authors must tune the scale to within an order of magnitude. Any sample is homogeneous as long as the scale is large enough. For right panel of Fig. 1f, how many SHG curves at different locations are superimposed?

10) In Fig. 2b and Fig. S11, why are there some irregular mosaic backgrounds in the LEED images in addition to the diffraction spots?

11) To support the self-limiting growth, the authors should show more AFM images. For each growth time (over four different growth times), the authors should present AFM images of more than 10 random positions on the wafer.

12) For the sentence "High-resolution in-plane XRD ϕ scans at five different points in the wafer, as shown in Figure 3d, macroscopically revealed the formation of uniform film without low-angle and antiparallel domains throughout the wafer", the XRD data cannot demonstrate that there are no antiparallel domains.

13) For transfer curves (Fig. 4b) and output characteristics (Fig. 4c), why do the authors use samples grown at two different temperatures? They should standardize the samples and present data from a unified sample.

14) In page 14, the authors state: "mobilities achieved via MOCVD are comparatively lower than those obtained using powder CVD, rendering small comparisons of μ values at room

temperature less meaningful". I cannot agree with the authors' view. Mobility is an intrinsic property of a sample, which is not determined by the growth method. If the mobility of samples obtained by a growth method is lower than that of other methods, this method has no application value and should be eliminated. The authors should explain why the mobility of MOCVD samples is comparatively lower than those obtained using powder CVD—whether it is due to too many defects in the samples or other reasons.

15) The authors claim the phonon limited electron mobility, I also do not agree with this because we cannot simply draw this conclusion directly from the temperature evolution of mobility. The temperature evolution can only indicate whether phonons come into play. In addition, the obtained mobility is lower than the phonon limit value.

16) Regarding "attains a record-high mobility, approximately 66 cm^2 /Vs", I suggest the authors remove the claim of "record-high".

17) Regarding "approaches values similar to those achieved previously with powder CVD[46]", I suggest the authors cite the most recent literature. Ref. 46 cited by the authors is already 8 years old and does not represent state-of-the-art results.

18) For the second paragraph in the main text "Since c-plane of bulk sapphire exhibits……and other techniques", the logic of the writing is very confusing. First, the authors state in the first sentence that based on basic symmetry guideline it should be possible to realize MoS_2 single crystals on sapphire. However, in the second sentence immediately following, the authors go on to say that simply on the basis of basic symmetry guideline, the experiment did not realize MoS_2 single-crystal. Are the authors trying to illustrate the basic symmetry guideline in the first sentence, or are they trying to show by the second sentence that the basic symmetry guideline is wrong now? Subsequently, the authors highlight that two major strategies known as step-edge-guided and symmetry-guided epitaxy have been proposed. In fact, both strategies are within the framework of basic symmetry guideline (i.e., the first sentence).

19) For the third paragraph in the main text, it is also very confusing. This is because the authors raised many key scientific problems in this passage, but then the authors don't address any of them.

20) In line 21, the authors write about "quasi-van der Waals (vdW) epitaxy". What does "quasi" mean? I am confusing for the use of word "quasi".

21) For "LEED intensity - voltage", there are two redundant spaces.

22) Regarding Raman mapping of Fig. 3e, why does it resemble a scatter plot?
